# Active surveillance for adverse events in patients on longer treatment regimens for multidrug-resistant tuberculosis in Viet Nam

**DOI:** 10.1371/journal.pone.0255357

**Published:** 2021-09-07

**Authors:** Nguyen Bao Ngoc, Hoa Vu Dinh, Nguyen Thi Thuy, Duong Van Quang, Cao Thi Thu Huyen, Nguyen Mai Hoa, Nguyen Hoang Anh, Phan Thuong Dat, Nguyen Binh Hoa, Edine Tiemersma, Nguyen Viet Nhung

**Affiliations:** 1 National Tuberculosis Programme, Hanoi, Viet Nam; 2 Department of Pharmacy, National Lung Hospital, Hanoi, Viet Nam; 3 National Drug Information and Adverse Drug Reaction Monitoring Centre, Hanoi University of Pharmacy, Hanoi, Viet Nam; 4 KNCV Tuberculosis Foundation, Den Haag, The Netherlands; Harvard Medical School, UNITED STATES

## Abstract

**Objective:**

Management of multidrug-resistant tuberculosis (MDR-TB) is a significant challenge to the global healthcare system due to the complexity and long duration of the MDR-TB treatment. This study analyzed the safety of patients on longer injectable-based MDR-TB treatment regimens using active pharmacovigilance data.

**Method:**

We conducted an observational, prospective study based on active pharmacovigilance within the national TB program. A total of 659 MDR-TB patients were enrolled and followed up at 9 TB- hospitals in 9 provinces of all 3 regions in Vietnam between 2014 and 2016. Patients received a treatment regimen (standardized or individualized) based on their drug susceptibility test result and their treatment history. Baseline and follow-up information was collected at the start and during treatment. Adverse events (AE) were defined and classified as serious adverse events (SAEs) or otherwise. Multivariate Cox regression following the Iterative Bayesian Model Averaging algorithm was performed to identify factors associated with AE occurrence.

**Results:**

Out of 659 patients assessed, 71.3% experienced at least one AE, and 17.5% suffered at least one SAE. The most common AEs were gastrointestinal disorders (38.5%), arthralgia (34.7%), and psychiatric disorders (30.0%). The proportion of patients with nephrotoxicity and hearing loss or vestibular disorders were 7.4% and 15.2%, respectively. 13.1% of patients required modifications or interruption of one or more drugs. In 77.7% of patients, treatment was completed successfully, while 9.3% lost to follow-up, in 3.0% treatment failed, and 7.4% died. Some significant risk factors for nephrotoxicity included diabetes mellitus (HR = 8.46 [1.91–37.42]), renal dysfunction (HR = 8.46 [1.91–37.42]), alcoholism (HR = 13.28 [5.04–34.99]), and a higher average daily dose of injectable drugs (HR = 1.28 [1.14–1.43]).

**Conclusion:**

While a majority of patients on the longer injectable-based regimens experienced non-serious AEs during MDR-TB treatment, one in six patients experienced at least an SAE. Active TB drug-safety monitoring is useful to understand the safety of MDR-TB treatment and explore the risk factors for toxicity. All-oral, shorter MDR-TB regimens might be able to reduce the inconvenience, discomfort, and toxicity of such regimens and increase adherence and likelihood of successful completion.

## Introduction

Multidrug-resistant tuberculosis (MDR-TB) is a significant threat to global healthcare systems and TB control. According to the statistics of the World Health Organization (WHO) in 2020, there were close to 500 000 new cases with resistance to rifampicin (RR-TB), most of whom also had isoniazid resistance (multidrug-resistant TB; MDR-TB) [1]. However, the global treatment success rates for MDR-TB and extensively drug-resistant TB (XDR-TB) were low at 57% and 26%, respectively [1, 2]. Vietnam is among 30 high-burden MDR-TB countries with an estimate of 8 400 new MDR-TB cases among a total of around 170 000 new TB cases per year [1]. MDR-TB treatment is complicated, expensive, requires a combination of multiple anti-TB drugs, of which some are highly toxic, and some regimens last 20 months or more [2]. However, data on the safety profile of these regimens was mainly reported from crossectional surveys or single sites [3–6]. To have a comprehensive picture of adverse events (AEs) that MDR-TB patients sustained during the whole period of treatment, cohort event monitoring (CEM) was set up for the National Tuberculosis Programme (NTP). CEM is a prospective, observational, cohort study of adverse events associated with one or more medicines [7]. In our study, CEM was used to actively detect AEs during MDR-TB treatment, assess causality and improve the clinical management of the patients.

AEs associated with treatment of MDR-TB can be significant and need to be monitored and managed properly and timely, thus being a critical component of the programmatic management of drug-resistant TB [8]. Among the most consequential AEs, nephrotoxicity and ototoxicity associated with injectable drugs (kanamycin, amikacin, or capreomycin) require adequate monitoring to avoid permanent disability or serious consequences for patients. Even though WHO has recommended restricted use of the injectable agents in the most recent guidelines, some individualized regimens in current use may still include these drugs [9, 10]. It is essential to understand factors related to the toxicity of injectable agents as it helps build a strategy to minimize the risk. Nephrotoxicity of injectable drugs in clinical studies is well established; however, there is a lack of studies about the association of these drugs in MDR-TB treatment with nephrotoxicity. Therefore, this study aims to describe patient characteristics, detect AEs and evaluate nephrotoxicity of injectable drugs in MDR-TB treatment.

## Methods

### Patient population

An observational, prospective study using a CEM approach [7] was conducted with 9 NTP MDR-TB treatment sites, which were hospitals in 9 provinces across all three regions (North, Middle, and South) of Vietnam. Patients were enrolled in this cohort if they: (1) had been diagnosed with MDR/RR-TB based on drug susceptibility testing; (2) were 16 years or older; (3) had never been exposed to MDR-TB treatment before, and (4) were initiated on MDR-TB treatment in one of the selected NTP sites. AE monitoring and management were implemented for the whole duration of treatment for all patients. The 9 hospitals involved in this study were Pham Ngoc Thach Hospital (Ho Chi Minh City), Hanoi Lung Hospital, Central Hospital Number 74, Thanh Hoa Lung Hospital, Pham Ngoc Thach Hospital (Quang Nam province), and 4 provincial Hospitals of Tuberculosis and Lung Disease of Binh Dinh, Can Tho, Nam Dinh and Binh Thuan.

### Treatment

According to the NTP guideline for MDR-TB treatment, patients receive a standardized regimen based on drug susceptibility test results and their treatment history. In 2014, the standardized regimen consisted of an initial phase of 8 months with six drugs, including an injectable agent [kanamycin (Km) or capreomycin (Cm)], levofloxacin (Lfx), prothionamide (Pto), cycloserine (Cs) (or *p*-aminosalicylic acid/PAS), pyrazinamide (PZA) and ethambutol (Emb), followed by a continuation phase of up to 18 months in which the same drugs were provided, except the injectable drug agent. Km and Cs were replaced by Cm and PAS, respectively, if patients did not tolerate these drugs. Phenotypic drug susceptibility tests were conducted to assess if a strain was resistant to anti-TB drugs. An individualized regimen was used if there was resistance to any drugs in the standardized regimen (e.g. injectable agents, fluoroquinolones) or drug intolerance. The timeline for MDR-TB patient management followed the Guideline for TB diagnosis, treatment, and prevention adopted by Ministry of Health of Vietnam (S1 Table).

### Data collection

All MDR-TB physicians in the selected sites were trained on data collection using standardized forms and procedures. Prior to the start of MDR-TB treatment, baseline patient information was collected by MDR-TB clinicians in a paper-based form, which was then sent to the National Drug Information and Adverse Drug Monitoring Center of Vietnam. During the follow-up, trained physicians monitored and reported AEs according to a standard procedure developed by NTP. Patients were hospitalized in the first 2 weeks or up to 2 months depending on their clinical condition and subsequently continued treatment as outpatients with monthly visits to the treatment center. AEs were detected based on symptoms or abnormal laboratory results registered on the so-called AE form. This form collected information about AE, including AE description, time of occurrence, severity, respective medical intervention, and information about current MDR-TB regimen and the concomitant drugs. For outpatients, AE monitoring was done during the routine monthly visits plus any additional visits for other reasons. Pre-designed data collection forms were provided to all participating sites (S1 File). The reported AEs were evaluated and classified using a version of the WHO guideline [7] adapted to the situation of the treatment system in Vietnam. Criteria to identify AEs are summarized in S2 Table.

Serious adverse events (SAEs) were identified if those events led to any of the following consequences: hospitalization, prolongation of hospitalization, a persistent significant disability, a congenital anomaly, a life-threatening condition, or death [7].

### Data management and analysis

A Microsoft Access database was designed for data entry and management. The quality of data entry was checked quarterly by randomly double-checking 20% of records by a member of the research team not involved in the data entry. A systematic cleaning and recoding process was applied using the Syntax tool of SPSS 22.0 [11]. Missing data were handled using the Multivariate Imputation by Chained Equations (MICE) approach available in the mice 3.3.0 package on R 3.5.1 [12, 13]. Multivariate Cox regression following the Iterative Bayesian Model Averaging (BMA) algorithm was performed to identify factors associated with AE occurrence [14, 15]. The proportional hazard assumption was assessed by Schoenfeld global test [16]. Frequencies were derived using overall percentages and medians and interquartile ranges (IQR) for continuous variables. Hazard ratios with 95% confidence limits were used to express risk. Associations with a P value less than 0.05 were considered to be statistically significant.

### Ethics statement

The proposal of this study was given scientific and ethical approval by the Committee of Hanoi University of Pharmacy, under approval letter number 155a/QD-DHN on 24 March 2014. Due to the nature of the observational study, the informed consent was waived. The reporting of the study adheres to the Strengthening the Reporting of Observational Studies in Epidemiology (STROBE) guidelines for observational studies [17] (S3 Table).

## Results

Of 659 MDR/RR-TB patients enrolled in the cohort, most patients (95.8%) received a standardized regimen containing kanamycin. There were 6 patients received individualized regimens, of which 2 patients were treated with regimens containing amikacin. The median treatment duration was 19.3 (Interquartile range [IQR], 17.7–20.2) months. The characteristics of patients in the cohort are presented in Table 1.

**Table 1:** Clinical characteristics of patients.

Information	n (%) or Median (IQR^a^) (N = 659)
Male sex	517 (78.5)
Age in years	41 (31–53)
Weight in kg	47 (42–54)
Duration of treatment in months	19.3 (17.7–20.2)
Outcome of previous treatment	
Completed	278 (42.2)
Treatment failure	259 (39.3)
Lost to follow-up	22 (3.3)
No history of TB treatment	50 (7.6)
No information	50 (7.6)
Pre-existing comorbidities	
Diabetes mellitus	104 (15.8)
HIV infection	57 (8.6)
Hepatic disorders	33 (5.0)
Gastrointestinal disorders	12 (1.8)
Hypertension	12 (1.8)
Hearing loss	11 (1.7)
Arthralgia	7 (1.1)
Renal dysfunction	5 (0.8)
Pre-existing patient conditions	
Fatigue	88 (13.4)
Alcohol dependence	16 (2.4)
Initial MDR-TB Treatment Regimens	
Standardized regimen 1^b^ *(kanamycin based)*	631 (95.8)
Standardized regimen 2^c^ *(capreomycin based)*	22 (3.3)
Individualized^d^	6 (0.9)

IQR^a^: interquartile range.

*Drug abbreviations*: kanamycin (Km) or capreomycin (Cm), levofloxacin (Lfx), prothionamide (Pto), cycloserine (Cs), p-aminosalicylic acid (PAS), pyrazinamide (PZA) and ethambutol (Emb). The composition at the start of treatment of the regimens was as follows

*Regimen 1*^*b*^: PZA, Emb, Km, Lfx, Pto, Cs (PAS).

*Regimen 2*^*c*^: PZA, Emb, Cm, Lfx, Pto, Cs (PAS).

*Individualized*^*d*^: Pza Emb Am Mfx Pto Cs PAS (n = 2); PZA Emb Km Lfx Pto (n = 2); Emb Km Lfx Cs PZA (n = 1); PZA Lfx Pto Cs Km (n = 1).

If patients did not tolerate Km and/or Cs, Cm and/or PAS would be the substitutions, respectively.

During treatment, 470 (71.3%) patients experienced at least one AE, and 115 (17.5%) of patients suffered at least one SAE during the treatment in Table 2. The most common AEs were gastrointestinal disorders (38.5%), arthralgia (34.7%), and psychiatric disorders (30.0%). Of all SAEs, hearing loss or vestibular disorders, and visual impairment were the most common, in 3.6% and 3.3% of the patients, respectively.

**Table 2:** Adverse events occurring during MDR-TB treatment.

Adverse event	AE^a^ n (%) (N = 659)	SAE^b^ n (%) (N = 659)
***At least one adverse event***	470 (71.3)	115 (17.5)
Gastrointestinal disorders	254 (38.5)	18 (2.7)
Arthralgia	229 (34.7)	5 (0.8)
Psychiatric disorders	222 (33.7)	18 (2.7)
Central nervous system disorders	198 (30.0)	18 (2.7)
Hyperuricemia	193 (29.3)	4 (0.6)
Dermatologic reactions	119 (18.1)	4 (0.6)
Hearing loss or vestibular disorders	100 (15.2)	24 (3.6)
Visual impairment	69 (10.5)	22 (3.3)
Hypokalaemia	60 (9.1)	3 (0.5)
Peripheral neuropathy	52 (7.9)	1 (0.2)
Nephrotoxicity	49 (7.4)	10 (1.5)
Glucose metabolism disorders	42 (6.4)	18 (2.7)
Hepatoxicity	38 (5.8)	12 (1.8)
Hematologic disorders	23 (3.5)	4 (0.6)
Anaphylactic reactions	4 (0.6)	2 (0.3)
Hypothyroidism (n = 263)^c^	3 (1.1)	0 (0)

AE^a^: Adverse Event.

SAE^b^: Serious Adverse Event.

Hypothyroidism (n = 263)^c^: 263 patients had thyroid-stimulating hormone results at baseline and at least one measurement during the treatment.

Out of 115 patients with at least one SAE, 95 patients had to be hospitalized or had their hospitalization prolonged (Table 3). Visual impairment, hearing loss or vestibular disorders, and glucose metabolism disorders were the most common AEs leading to hospitalization or prolonging hospitalization, with 3.0%, 2.7%, and 2.7%, respectively. Hepatoxicity (6/659), psychiatric disorders (5/659), and nephrotoxicity (5/659) were the most frequent life-threatening AEs. Out of 8 patients (1.2%) experiencing permanent disability, seven patients had hearing loss, and one patient had visual impairment. PAS, Km, and Cs were the 3 drugs most often withdrawn and replaced, for 0.8%, 0.5% and 0.5% of patients, respectively. Dose reduction mainly occurred in patients treated with Km (2.4%) and PZA (1.8%). The anti-TB drugs that were most often discontinued permanently were PZA (3.9%), injectable drugs (2.9%), and Pto (2.7%). By the end of follow-up, 512 (77.7%) patients were reported to have been cured or completed their treatment. Unfavorable outcomes were reported for 130 patients (19.7%).

**Table 3:** Consequences of the adverse events and follow up outcomes of MDR-TB treatment.

**SAE** ^**a**^	**n (%) (N = 659)**
Hospitalization or prolongation of hospitalization	95 (14.4)
Life-threatening	24 (3.6)
Permanent disability	8 (1.2)
**AE** ^**b**^ **management**	**n (%) (N = 659)**
TB therapeutic intervention	86 (13.1)
*Withdrawal and replacement*	16 (2.4)
*Dose reduction*	29 (4.4)
*Permanent discontinuation*	53 (8.0)
Other medical intervention	338 (51.3)
**Follow-up outcomes**	**n (%) (N = 659)**
Cure/completion	512 (77.7)
Lost to follow-up	61 (9.3)
Failure	20 (3.0)
All-cause death	49 (7.4)
Not evaluated	17 (2.6)

SAE^a^: Serious Adverse Event.

AE^b^: Adverse Event.

**Fig 1:**
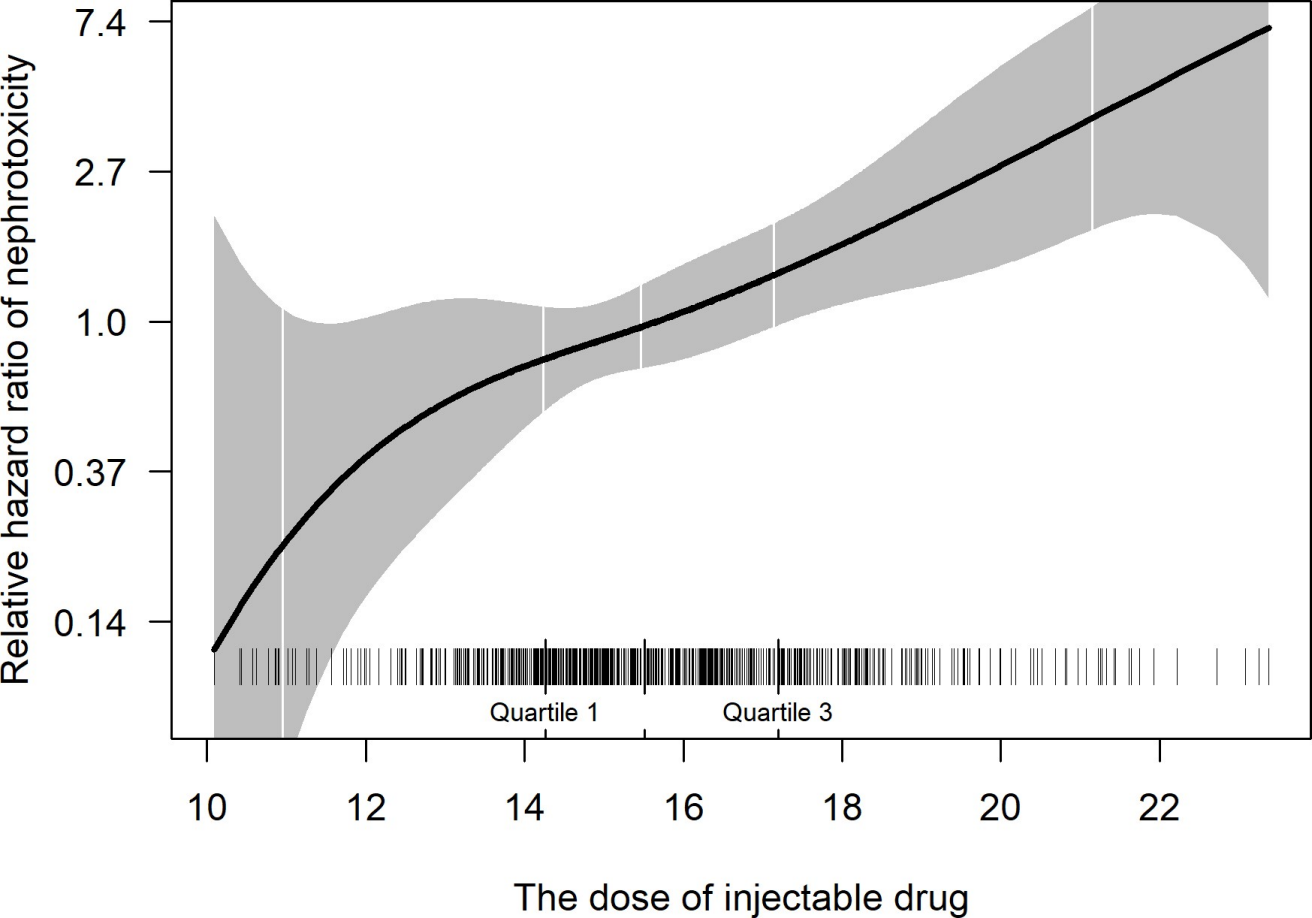
The relationship between the average daily dose of injectable drugs and nephrotoxicity. Relative hazard ratio (solid line) and 95% confidence intervals (shading) are estimated from the Cox model with injectable drug dose as a spline function. The model was adjusted for age, gender, body mass index, alcohol dependence, diabetes, and pre-existing renal disease. Patient distributions by dose are noted as small vertical ticks.

Diabetes mellitus, existing renal dysfunction, alcohol dependence, and a higher average daily dose of injectable drugs were identified to be associated with nephrotoxicity (Table 4). Each increment of 1mg/kg/day was associated with a 28% increase in the risk of nephrotoxicity, a positive relationship that was nearly linear over much of the range of observed values (Fig 1).

**Table 4:** Multivariate analysis for risk factors associated with the occurrence of nephrotoxicity.

Variables *(With AE*^*a*^*/all subjects)*	HR^b^ (95%CI^c^)	p-value
**Diabetes mellitus**		
No *(36/555)*	1 (Reference)	-
Yes *(13/104)*	2.03 (1.03–4.02)	0.042
**Existing renal dysfunction**		
No *(47/654)*	1 (Reference)	-
Yes *(2/5)*	8.46 (1.91–37.42)	0.005
**Alcohol dependence**		
No *(43/643)*	1 (Reference)	-
Yes *(6/16)*	13.28 (5.04–34.99)	<0.001
**Increments of 1mg/kg/day of injectable drugs** ^**d**^	1.28 (1.14–1.43)	<0.001

AE^a^: Adverse event.

HR^b^: Hazard ratio are adjusted for age, gender, BMI.

CI^c^: Confidence interval.

Injectable drugs^d^: including amikacin or kanamycin or capreomycin.

## Discussion

The emergence of MDR-TB is a serious global threat to TB control, and treatment of MDR-TB is challenging because it usually requires longer regimens using more toxic second-line TB medicines than drug-susceptible TB. Active pharmacovigilance is a useful approach to detect AEs in MDR-TB treatment. In this study, 71.3% of patients experienced at least one AE, and 17.5% suffered at least one SAE during the treatment. Out of 659 patients, 86 (13.1%) needed any change to their regimens, including change of drugs (2.4%), dose reduction (4.4%), and drug discontinuation (8.0%). On the other hand, we also recorded 512 (77.7%) patients with favorable treatment outcomes (cured and treatment completed). Diabetes mellitus, excessive alcohol use, and pre-existing renal dysfunction had a statistically significant association with nephrotoxicity. We also observed a nearly linear relationship between the dose of the injectable and the hazard ratio of nephrotoxicity.

The frequency of AEs reported for patients on MDR-TB treatment varies between studies [6, 18, 19]. A systematic review and meta-analysis reported 57.3% of MDR-TB patients having at least one AE compared with 71.3% in our study [20]. Another study on MDR-TB patients treated with long regimens with injectable agents reported 807/1027 cases (79%) experienced at least 1 AE [21]. These differences between studies are likely due to differences in patient characteristics, treatment regimens, and AE monitoring approaches. In this study, AEs were monitored prospectively following the CEM approach. Nearly all patients were treated with the standardized MDR-TB regimen instead of an individualized regimen.

Gastrointestinal disorders and arthralgia were the most common AEs, with 38.5% and 34.7%, respectively, in this study. These results were consistent with the previous study (35.8%) in Viet Nam in 2015 [4] however, higher than reported in other studies (18–24%) [6, 18]. Nearly all patients in this study were treated with Pto in their regimens. This could lead to an increase in gastrointestinal disorders. Generally speaking, AEs related to clinical symptoms are usually detected easily and have high frequencies. Psychiatric disorders, central nervous system disorders developed in 33.7%, respectively 30.0% of patients in our study, while other studies showed lower proportions [5, 19, 20]. Possible reasons are that most of the patients in our study had Cs and Lfx in their regimens, and differences in definitions of AEs. These drugs were associated with AEs of psychiatric nature and central nervous system disorders. Central nervous system disorders usually occur in 0.9–11% of adults treated with fluoroquinolones [22]. And 20–30% of patients treated with Cs in other studies have had psychiatric disorders [22]. The percentage of patients having hepatotoxicity was 5.8%, which was consistent with results reported by Yang et al. [6] and the meta-analysis of Wu et al. [20]. Visual impairment was quite common at 10.5% in this study, compared to 2.5% in others [5, 19]. This AE usually occurs in persons receiving Emb [23, 24]. It is possible that patients in our study used a higher range of Emb doses. Moreover, the criteria to detect visual disorders might be different between studies. In a multi-center observational study like this, visual impairment was mainly reported when there were complaints about symptoms. The late-stage diagnosis of this AE showed a shortage of resources and trained staff.

In our study, ototoxicity, including hearing loss or vestibular disorders, was mainly detected by physical signs and symptoms instead of audiometry. Some cases could be diagnosed by audiometry but not reported in the end because patients were transferred to specialized hospitals/clinics, which were out of our monitoring. Although many studies, which used this method for evaluating ototoxicity, reported similar proportions of this AE with our study (15.2%) [25, 26], these results could be underestimated in comparison with the proportion of 20–30% in studies using audiogram to confirm ototoxicity [27, 28].

In this study, patients with nephrotoxicity were higher than in other studies [5, 6, 19], likely because AEs were actively monitored by measuring serum creatinine levels in all patients at baseline and each monthly visit. The mechanisms of aminoglycoside-induced ototoxicity and nephrotoxicity are well understood [29, 30]. We also observed that diabetes mellitus, existing renal dysfunction, alcohol dependency, and a higher average daily dose of injectable drugs were associated with a higher risk of nephrotoxicity. Previous studies showed that diabetes mellitus increased the odds of MDR-TB and the odds of developing AEs such as nephrotoxicity and hypothyroidism [31, 32]. Patients with alcohol dependency have a higher risk of nephrotoxicity than other patients. Alcohol and its metabolites could directly affect the kidneys and indirectly damage other organs (especially the liver) [33]. Higher daily doses of injectable drugs are related to a higher risk of nephrotoxicity [34, 35]. Additionally, patients treated with injectable medications usually experience more AEs or SAEs and poorer outcomes [24]. Therefore, except amikacin, these drugs are not recommended in the most recent WHO guideline for the treatment of MDR-TB [24, 36, 37]. In case these drugs are required for treatment, an adequate dose adjustment and frequent monitoring of potential hearing loss and nephrotoxicity need to be done. Our results suggested that we can develop a prognostic model to predict AEs and propose recommendations such as clarifying the risk of AEs at baseline, screening actively and frequently AEs each outpatient visit, managing properly AEs to minimize consequences of AEs during TB treatment.

In this study, 86/659 (13.1%) patients required TB regimen changes, and 338/659 (51.3%) patients needed additional medications or medical intervention(s) to manage AEs. The proportion of patients requiring a change in MDR-TB regimens in our study was lower than reported in other studies, ranging between 20.1% and 55.2% [19, 20, 38]. In a recent meta-analysis, 23.5% of patients had at least one drug stopped because of AEs, showing a higher rate than in our study [37]. This has its roots in differences in the patient population and underlying resistance patterns or a higher “tolerability” for AEs as well as more conservatism in changing the regimens. All studies emphasized the significance of managing patients closely and monitoring AEs. Clinical practitioners should be trained to recognize AEs at an early stage, which helps avoid serious consequences of AEs and reduce interruption or changes to MDR-TB therapy. Close monitoring and timely management may be a reason for the high treatment success rate in our study (77.7%) compared to approximately 60% reported elsewhere [2]. No follow-up for relapse over the 12 months after successful end of treatment was carried out as our study was conducted under routine programmatic conditions. With 49 (7.4%) deaths, the mortality rate in our study was comparable to that reported from previous studies [19, 39, 40].

The findings of studies such as ours reinforce the WHO position adopted in recent years to recommend all-oral treatment regimens as the first choice for MDR-TB patients [24]. Global policy was updated when data from MDR-TB patients treated with regimens without injectable agents–which have been standard regimen components for many years–showed them to be effective, less toxic and feasible under programmatic settings. Moreover, since then, all-oral regimens much shorter than 18 months have also been shown to be effective and are being recommended [24]. Improved access to potent agents like later generation fluoroquinolones, bedaquiline and linezolid have made these regimens increasingly possible in most high TB burden settings.

There are several limitations to our study. First, AEs that are clinically determined may present a challenge to standardization. Although all researchers and clinicians were trained on how to record and report AEs, finding AEs in MDR-TB treatment depends on many factors, including experience, local cultural habits, and knowledge of physicians. Second, there was no regular monitoring possible for some laboratory tests (thyroid stimulating hormone, amylase) at some study sites due to lack of funding and facilities. Besides, ototoxicity and visual impairment in our study were mainly evaluated through clinical symptoms instead of medical equipment. This may result in underestimating the incidence of AEs and difficulty in managing consequences of AEs. Third, we did not evaluate the causal relationship between all AEs and anti-TB drugs because the initial aim was to build a model for the recording and reporting of AEs in MDR-TB treatment in Viet Nam rather than conducting pharmacovigilance. Last, the safety information in this study is from patients treated by longer regimens containing injectable drugs, which is not recommended by the most recent guideline of WHO. However, this study still has its merit because, in clinical practice, there are still some patient populations who need to be treated with regimens including injectable drugs due to intolerance of newer anti-TB drugs such as bedaquiline and delamanid. Further studies could use findings from this study as a control group or a comparator about safety between longer treatment regimens and new therapies. Experience obtained from the implementation of active pharmacovigilance during the treatment of MDR-TB helped develop national guidelines on the management of AEs in the treatment of MDR-TB, aligned to the principles of active tuberculosis drug-safety monitoring and management (aDSM) proposed by WHO [41]. This emphasizes the role of active pharmacovigilance to detect and treat AEs promptly, especially when patients are treated with new drugs and new regimens.

From our experience, CEM study should only be conducted when researchers expect to measure detailed information on AEs (incidence rate per exposure, onset time, progression, severity, seriousness, characteristics, risk factors, concomitant diseases). It is important to stress that the proper conduct of this approach requires investment in terms of financial resources and human effort due to the long duration of TB treatment, the high frequency of AEs, and the complexity of anti-TB regimens. We recommend that AEs of interest, including ototoxicity and visual impairment, need to be detected through medical equipment such as audiograms or specialized tests in each outpatient visit when high-risk drugs are used. In many cases, it might be too late for AE management if diagnoses of hearing loss and visual impairment only depend on physical signs and symptoms because these AEs could be irreversible and have detrimental effects on quality of life. CEM programs must be well designed, including collaborations among different specialists such as cardiology, hematology, ophthalmology, and audiology. All physicians and practitioners need to be updated and trained to recognize, manage, and report AEs. More importantly, safety surveillance programs of anti-TB treatments require regular supervision and monitoring. Under limited resources, alternative pharmacovigilance approaches should be used in national TB programs, such as those focused on SAEs or AEs of interest based on aDSM [41] or preferably including sensitization of clinicians to get a full safety profile.

## Conclusion

AEs are common during the MDR-TB treatment using injectable-based longer regimens. While the majority of patients on the longer injectable-based regimens experienced non-serious AEs during MDR-TB treatment, one in six patients experienced at least one SAE. It is crucial to monitor and manage AEs, especially serious ones, to provide proper clinical decisions and improve patient care. Whenever injectable drugs are used, which are not recommended nowadays, these should be prescribed at the lowest possible dose to avoid serious toxicity, while extra caution is needed in patients with existing renal dysfunction, diabetes mellitus, and alcohol dependency. Active TB drug-safety monitoring is useful in improving the monitoring and management of AEs among patients on MDR-TB treatment. The findings from our study have documented further why MDR-TB patients need safer choices of therapy to improve medication adherence and treatment outcomes.

## Supporting information

S1 Table Timeline for MDR-TB treatment. (DOCX)

S2 Table Definitions of adverse events. (DOCX)

S3 Table STROBE statement—checklist of items that should be included in reports of *cohort studies*. (DOCX)

S1 File Form for collection initiation information of MDR-TB patient. (PDF)

S2 File Data. (XLSX)

S3 File (ZIP)

S4 File R code for data analysis. (DOCX)

## References

[1] World Health Organization. Global tuberculosis report 2020. WHO Press. 2020. https://www.who.int/tb/publications/global_report/en/.

[2] Bastos ML, Lan Z, Menzies D. An updated systematic review and meta-analysis for treatment of multidrug-resistant tuberculosis. The European respiratory journal. 2017;49(3). doi: 10.1183/13993003.00803-2016 28331031

[3] Gualano G, Mencarini P, Musso M, Mosti S, Santangelo L, Murachelli S, et al. Putting in harm to cure: Drug related adverse events do not affect outcome of patients receiving treatment for multidrug-resistant Tuberculosis. Experience from a tertiary hospital in Italy. PloS one. 2019;14(2):e0212948. doi: 10.1371/journal.pone.0212948 30817779 PMC6394924

[4] Hoa NB, Nhung NV, Khanh PH, Hai NV, Quyen BT. Adverse events in the treatment of MDR-TB patients within and outside the NTP in Pham Ngoc Thach hospital, Ho Chi Minh City, Vietnam. BMC research notes. 2015;8:809. doi: 10.1186/s13104-015-1806-4 26695761 PMC4687360

[5] Prasad R, Singh A, Srivastava R, Hosmane GB, Kushwaha RA, Jain A. Frequency of adverse events observed with second-line drugs among patients treated for multidrug-resistant tuberculosis. The Indian journal of tuberculosis. 2016;63(2):106–14. doi: 10.1016/j.ijtb.2016.01.031 27451820

[6] Yang TW, Park HO, Jang HN, Yang JH, Kim SH, Moon SH, et al. Side effects associated with the treatment of multidrug-resistant tuberculosis at a tuberculosis referral hospital in South Korea: A retrospective study. Medicine. 2017;96(28):e7482. doi: 10.1097/MD.0000000000007482 28700490 PMC5515762

[7] World Health Organization. A practical handbook on the pharmacovigilance of antituberculosis medicines. WHO Press. 2012. https://www.who.int/tb/publications/tb-pharmacovigilance-handbook/en/.

[8] World Health Organization. Companion handbook to the WHO guidelines for the programmatic management of drug-resistant tuberculosis. WHO Press. 2014. https://www.who.int/tb/publications/pmdt_companionhandbook/en/. 25320836

[9] Cohen KA, Stott KE, Munsamy V, Manson AL, Earl AM, Pym AS. Evidence for Expanding the Role of Streptomycin in the Management of Drug-Resistant Mycobacterium tuberculosis. Antimicrobial agents and chemotherapy. 2020;64(9). doi: 10.1128/AAC.00860-20 32540971 PMC7449167

[10] Semuto Ngabonziza JC, Van Deun A, Migambi P, Belamo NE, Théogène D, Habimana YM, et al. Case Report: Dynamics of Acquired Fluoroquinolone Resistance under Standardized Short-Course Treatment of Multidrug-Resistant Tuberculosis. 2020. doi: 10.4269/ajtmh.20-0201 32618257 PMC7543851

[11] IBM Corp. IBM SPSS Statistics for Windows, Version 22.0. Armonk, NY: IBM Corp. 2013. https://www.ibm.com/analytics/spss-statistics-software.

[12] Buuren S, Groothuis-Oudshoorn C. MICE: Multivariate Imputation by Chained Equations in R. Journal of Statistical Software. 2011;45. doi: 10.18637/jss.v045.i01 22289957 PMC3267837

[13] R Core Team. R: A language and environment for statistical computing. R Foundation for Statistical Computing, Vienna, Austria. 2018. https://www.R-project.org/.

[14] Genell A, Nemes S, Steineck G, Dickman PW. Model selection in Medical Research: A simulation study comparing Bayesian Model Averaging and Stepwise Regression. BMC Medical Research Methodology. 2010;10(1):108. doi: 10.1186/1471-2288-10-108 21134252 PMC3017523

[15] Hoeting JA, Madigan D, Raftery AE, Volinsky CT. Bayesian Model Averaging: A Tutorial. Statistical Science. 1999;14(4):382–401. doi: 10.1214/ss/1009212519

[16] Xue X, Xie X, Gunter M, Rohan TE, Wassertheil-Smoller S, Ho GYF, et al. Testing the proportional hazards assumption in case-cohort analysis. BMC Medical Research Methodology. 2013;13(1):88. doi: 10.1186/1471-2288-13-88 23834739 PMC3710085

[17] von Elm E, Altman DG, Egger M, Pocock SJ, Gøtzsche PC, Vandenbroucke JP. Strengthening the Reporting of Observational Studies in Epidemiology (STROBE) statement: guidelines for reporting observational studies. BMJ (Clinical research ed). 2007;335(7624):806–8. doi: 10.1136/bmj.39335.541782.AD 17947786 PMC2034723

[18] Ahmad N, Javaid A, Syed Sulaiman SA, Afridi AK, Zainab, Khan AH. Occurrence, Management, and Risk Factors for Adverse Drug Reactions in Multidrug Resistant Tuberculosis Patients. American journal of therapeutics. 2018;25(5):e533–e40. doi: 10.1097/MJT.0000000000000421 26938643

[19] Zhang Y, Wu S, Xia Y, Wang N, Zhou L, Wang J, et al. Adverse Events Associated with Treatment of Multidrug-Resistant Tuberculosis in China: An Ambispective Cohort Study. Medical science monitor: international medical journal of experimental and clinical research. 2017;23:2348–56. doi: 10.12659/msm.904682 28520704 PMC5444822

[20] Wu S, Zhang Y, Sun F, Chen M, Zhou L, Wang N, et al. Adverse Events Associated With the Treatment of Multidrug-Resistant Tuberculosis: A Systematic Review and Meta-analysis. American journal of therapeutics. 2016;23(2):e521–30. doi: 10.1097/01.mjt.0000433951.09030.5a 24284652

[21] Bloss E, Kuksa L, Holtz TH, Riekstina V, Skripconoka V, Kammerer S, et al. Adverse events related to multidrug-resistant tuberculosis treatment, Latvia, 2000–2004. The international journal of tuberculosis and lung disease: the official journal of the International Union against Tuberculosis and Lung Disease. 2010;14(3):275–81. https://pubmed.ncbi.nlm.nih.gov/20132617/. 20132617

[22] Ramachandran G, Swaminathan S. Safety and tolerability profile of second-line anti-tuberculosis medications. Drug safety. 2015;38(3):253–69. doi: 10.1007/s40264-015-0267-y 25676682

[23] Ezer N, Benedetti A, Darvish-Zargar M, Menzies D. Incidence of ethambutol-related visual impairment during treatment of active tuberculosis. The international journal of tuberculosis and lung disease: the official journal of the International Union against Tuberculosis and Lung Disease. 2013;17(4):447–55. doi: 10.5588/ijtld.11.0766 23394767

[24] World Health Organization. WHO consolidated guidelines on drug-resistant tuberculosis treatment. WHO Press. 2019. https://www.who.int/tb/publications/2019/consolidated-guidelines-drug-resistant-TB-treatment/en/. 30946559

[25] Keshavjee S, Gelmanova IY, Farmer PE, Mishustin SP, Strelis AK, Andreev YG, et al. Treatment of extensively drug-resistant tuberculosis in Tomsk, Russia: a retrospective cohort study. Lancet (London, England). 2008;372(9647):1403–9. doi: 10.1016/S0140-6736(08)61204-0 18723218

[26] Shin SS, Pasechnikov AD, Gelmanova IY, Peremitin GG, Strelis AK, Mishustin S, et al. Adverse reactions among patients being treated for MDR-TB in Tomsk, Russia. The international journal of tuberculosis and lung disease: the official journal of the International Union against Tuberculosis and Lung Disease. 2007;11(12):1314–20. 18034952

[27] Modongo C, Sobota RS, Kesenogile B, Ncube R, Sirugo G, Williams SM, et al. Successful MDR-TB treatment regimens including Amikacin are associated with high rates of hearing loss. BMC Infectious Diseases. 2014;14(1):542. doi: 10.1186/1471-2334-14-542 10.1186/1471-2334-14-542. 25300708 PMC4287509

[28] Seddon JA, Godfrey-Faussett P, Jacobs K, Ebrahim A, Hesseling AC, Schaaf HS. Hearing loss in patients on treatment for drug-resistant tuberculosis. The European respiratory journal. 2012;40(5):1277–86. doi: 10.1183/09031936.00044812 22700838

[29] Perazella MA. Drug-induced acute kidney injury: diverse mechanisms of tubular injury. Current opinion in critical care. 2019;25(6):550–7. doi: 10.1097/MCC.0000000000000653 31483318

[30] Selimoglu E. Aminoglycoside-induced ototoxicity. Current pharmaceutical design. 2007;13(1):119–26. doi: 10.2174/138161207779313731 17266591

[31] Muñoz-Torrico M, Caminero-Luna J, Migliori GB, D’Ambrosio L, Carrillo-Alduenda JL, Villareal-Velarde H, et al. Diabetes is Associated with Severe Adverse Events in Multidrug-Resistant Tuberculosis. Arch Bronconeumol. 2017;53(5):245–50. doi: 10.1016/j.arbres.2016.10.021 28089216

[32] Tegegne BS, Mengesha MM, Teferra AA, Awoke MA, Habtewold TD. Association between diabetes mellitus and multi-drug-resistant tuberculosis: evidence from a systematic review and meta-analysis. Systematic reviews. 2018;7(1):161. doi: 10.1186/s13643-018-0828-0 30322409 PMC6190557

[33] Varga ZV, Matyas C, Paloczi J, Pacher P. Alcohol Misuse and Kidney Injury: Epidemiological Evidence and Potential Mechanisms. Alcohol research: current reviews. 2017;38(2):283–8. https://www.ncbi.nlm.nih.gov/pmc/articles/PMC5513691/. 28988579 10.35946/arcr.v38.2.10 PMC5513691

[34] Mingeot-Leclercq MP, Tulkens PM. Aminoglycosides: nephrotoxicity. Antimicrobial agents and chemotherapy. 1999;43(5):1003–12. doi: 10.1128/AAC.43.5.1003 10223907 PMC89104

[35] Shibeshi W, Sheth AN, Admasu A, Berha AB, Negash Z, Yimer G. Nephrotoxicity and ototoxic symptoms of injectable second-line anti-tubercular drugs among patients treated for MDR-TB in Ethiopia: a retrospective cohort study. BMC Pharmacology and Toxicology. 2019;20(1):31. doi: 10.1186/s40360-019-0313-y 31122273 PMC6533713

[36] Borisov S, Danila E, Maryandyshev A, Dalcolmo M, Miliauskas S, Kuksa L, et al. Surveillance of adverse events in the treatment of drug-resistant tuberculosis: first global report. The European respiratory journal. 2019;54(6). doi: 10.1183/13993003.01522-2019 31601711

[37] Lan Z, Ahmad N, Baghaei P, Barkane L, Benedetti A, Brode SK, et al. Drug-associated adverse events in the treatment of multidrug-resistant tuberculosis: an individual patient data meta-analysis. The Lancet Respiratory medicine. 2020;8(4):383–94. doi: 10.1016/S2213-2600(20)30047-3 32192585 PMC7384398

[38] Baghaei P, Tabarsi P, Dorriz D, Marjani M, Shamaei M, Pooramiri MV, et al. Adverse effects of multidrug-resistant tuberculosis treatment with a standardized regimen: a report from Iran. American journal of therapeutics. 2011;18(2):e29–34. doi: 10.1097/MJT.0b013e3181c0806d 20019591

[39] Huerga H, Bastard M, Kamene M, Wanjala S, Arnold A, Oucho N, et al. Outcomes from the first multidrug-resistant tuberculosis programme in Kenya. The international journal of tuberculosis and lung disease: the official journal of the International Union against Tuberculosis and Lung Disease. 2017;21(3):314–9. doi: 10.5588/ijtld.16.0661 28225342

[40] Shean K, Streicher E, Pieterson E, Symons G, van Zyl Smit R, Theron G, et al. Drug-associated adverse events and their relationship with outcomes in patients receiving treatment for extensively drug-resistant tuberculosis in South Africa. PloS one. 2013;8(5):e63057. doi: 10.1371/journal.pone.0063057 23667572 PMC3646906

[41] World Health Organization. Active tuberculosis drug-safety monitoring and management (aDSM): Framework for implementation. WHO Press. 2015. https://www.who.int/tb/publications/aDSM/en/.

