# Supplementary material for: Active surveillance for adverse events in patients on longer treatment regimens for multidrug-resistant tuberculosis in Viet Nam
Source: PLoS One. 2021 Sep 7;16(9):e0255357. doi: 10.1371/journal.pone.0255357 (PMC8423256; doi:10.1371/journal.pone.0255357)
Supplement: S1 Table — (DOCX) [file pone.0255357.s001.docx]

| **Month** | **X-ray** | **AFB^a^** | **Culture** | **DST^b^** | **AST^c^/ALT/^d^**  **Bilirubin** | **Creatinine, ure, electrolytes** | **HIV** | **TSH^d^** | **Vision** | **Hearing** | **pregnancy test** | **CBC^e^** |
| --- | --- | --- | --- | --- | --- | --- | --- | --- | --- | --- | --- | --- |
| 0 | x | x | x | x | x | x | x | x | x | x | x | x |
| 1 |  | x | x |  | x | x | Do it again if it is necessary | If TSH is abnormal, T3/T4 will be examined | When it is neccessary | When it is neccessary | When it is neccessary |  |
| 2 |  | x | x |  | x | x |  |  |  |  |  | x |
| 3 |  | x | x |  | x | x |  |  |  |  |  |  |
| 4 | x | x | x | ± if sputum culture is positive | x | x |  |  |  |  |  | x |
| 5 |  | x | x |  | x | x |  |  |  |  |  |  |
| 6 |  | x | x |  | x | x |  |  |  |  |  | x |
| 7 |  | x | x |  | x | x |  |  |  |  |  |  |
| 8 | x | x | x |  | x | x |  | x |  |  |  | x |
| 9 |  | x |  |  |  |  |  |  |  |  |  |  |
| 10 |  | x |  |  |  |  |  |  |  |  |  |  |
| 11 |  | x | x |  | x | x |  |  |  |  |  | x |
| 12 |  | x |  |  |  |  |  |  |  |  |  |  |
| 13 |  | x |  |  |  |  |  |  |  |  |  |  |
| 14 | x | x | x |  | x | x |  | x |  |  |  | x |
| 15 |  | x |  |  |  |  |  |  |  |  |  |  |
| 16 |  | x |  |  |  |  |  |  |  |  |  |  |
| 17 |  | x | x |  | x | x |  |  |  |  |  | x |
| 18 |  | x |  |  |  |  |  |  |  |  |  |  |
| 19 |  | x |  |  |  |  |  |  |  |  |  |  |
| 20 | x | x | x |  | x | x |  | x |  |  |  | x |

**S1 Table. Timeline for MDR-TB treatment**

AFB^a^: Acid-fast bacillus; DST^b^: Drug Susceptibility Testing; AST^c^: Aspartate Aminotransferase; ALT^d^: Alanine Aminotransferase; TSH^d^: Thyroid Stimulating Hormone: CBC^e^: Complete Blood Count
