# Supplementary material for: Active surveillance for adverse events in patients on longer treatment regimens for multidrug-resistant tuberculosis in Viet Nam
Source: PLoS One. 2021 Sep 7;16(9):e0255357. doi: 10.1371/journal.pone.0255357 (PMC8423256; doi:10.1371/journal.pone.0255357)
Supplement: S2 Table — (DOCX) [file pone.0255357.s002.docx]

**S2 Table. Definitions of adverse events**

| **Adverse event** | **Symptoms associated in the literature with those events** |
| --- | --- |
| Gastrointestinal disorders | Any documentation of anorexia, nausea, vomiting, abdominal pain, diarrhea, and gastritis by a clinician |
| Central nervous system disorders | Headache, dizziness, or seizure activity of any type documented by the clinician |
| Psychiatric disorders | Presence of anxiety, depression, or psychosis diagnosed by clinician or psychologist |
| Peripheral neuropathy | Symptoms consistent with neuropathy such as pain, tingling, burning, or numbness of the extremities, as diagnosed by a clinician |
| Hearing loss or vestibular disorders | Tinnitus or hearing loss confirmed by audiometry or diagnosed by a clinician |
| Hepatic disorders | At least 1 elevated value of either serum transaminase or bilirubin ≥ 5 times above the normal upper limit without symptoms or ≥ 3 times above normal in the presence of symptoms |
| Dermatologic reactions | Any skin rash, pruritus, or photosensitivity reaction documented by clinician felt to be induced by anti-TB regimens |
| Nephrotoxicity | At least 1 evaluated value of serum creatinine ≥ 1.5 times the baseline, or creatinine clearance decreased by 25% of the baseline level |
| Hyperuricemia | At least 1 serum level of uric acid > 420 µmol/L (70 mg/dL) in men, > 360 µmol/L (60 mg/dL) in women |
| Visual impairment | Any documentation of visual changes including vision loss, eyesight decrease, or difficulty in distinguishing colors |
| Hematologic disorders | Any of hemoglobin < 12 g/dl in male or < 13 g/dl in female; Leukocytes < 3000×10^9^/l; platelets < 100×10^9^/l. |
| Hypokalemia | At least 1 serum potassium value of < 3.5 mEq/L |
| Hypothyroidism | Serum thyroid-stimulating hormone (TSH) > 5 IU/dl, serum level of total T4 < 64 mmol/L, with symptoms such as fatigue, cold intolerance, constipation, excessive menstrual bleeding, weight gain, dry skin, dry hair, distraction, loss of appetite |
| Arthralgia | Presence of pain, swelling, or stiffness in the joints as reported by clinician |
| Glucose metabolism disorders | Glucose < 3.55 mmol/l or > 6.11 mmol/l |
| Anaphylaxis reactions | a serious, life-threatening generalized or systemic hypersensitivity reaction |

*Note: ECG for QT interval prolongation was not done in this study due to lack of resources*
