# Supplementary material for: Active surveillance for adverse events in patients on longer treatment regimens for multidrug-resistant tuberculosis in Viet Nam
Source: PLoS One. 2021 Sep 7;16(9):e0255357. doi: 10.1371/journal.pone.0255357 (PMC8423256; doi:10.1371/journal.pone.0255357)
Supplement: S1 File — (PDF) [file pone.0255357.s004.pdf]

Patient's code:

FORM FOR COLLECTION INITIATION INFORMATION OF MDR-TB PATIENT

PATIENT DETAILS					
**Full name:**	**Sex:** ☐ M ☐ F	**Date of birth:** ____ /____ / ______	**Address:**	**Height:** cm	**Weight:** kg
**Register N ° MDR-TB:**	**ID No:**	**Occupation:**	**Local TB facility:**		

**Patient classification:**<br>☐ New<br>☐ Recurrence<br>☐ Failure I<br>☐ Failure II<br>☐ Treatment after losing to follow-up<br>☐ Transfer from another facility<br>☐ Other____________________________	**Patient situation:**<br>☐ Pregnant<br>☐ Breastfeeding an infant<br>☐ Alcohol abuse<br>☐ Drug abuse<br>☐ Malnitrition<br>☐ Other________________________<br>☐ None	**Other disease:**<br>☐ Epilepsy<br>☐ Psychosis<br>☐ Anaemia<br>☐ Diabetes<br>☐ Renal failure<br>☐ Liver failure<br>☐ Digestive disease<br>☐ Eye disease<br>☐ Auditory disease<br>☐ Musculoskeletal disease<br>☐ Thyroid gland disease<br>☐ Autoimmune Diseases<br>☐ Other___________________<br>☐ None<br>**HIV test**<br>☐ HIV(+) using ARV drugs<br>☐ HIV(+) without treatment<br>☐ HIV(-)<br>☐ No infomation	**History of drug allergy** ☐Yes ☐ No<br>If yes, please specify:<br><br>**Resistance TB- drugs (excluding isoniazid and rifampicin):**<br>☐ Streptomycin<br>☐ Ethambutol<br>☐ Pyrazinamid<br>☐ Amikacin<br>☐ Kanamycin<br>☐ Capreomycin<br>☐ Ofloxacin<br>☐ Other

HISTORY OF DRUG USE (within 30 days before treatment start)			EVENTS (within 30 days before treatment start)					
**Drug**	**Dosage**	**Frequency**	**Test**	**Date**	**Result**	**Test**	**Date**	**Result**
			RBC(G/l)			Kali (μmol/l)		
			Hb (g/dl)			Magie (μmol/l)		
			WBC (G/l)			Acid uric (μmol/l)		
			PLT(G/l)			Creatinin (μmol/l)		
			ALT (SGPT) (IU/l)			Ure (mmol/l)		
			AST (SGOT) (IU/l)			TSH (μU/l)		
			Total bilirubin (μmol/l)			Other		
			Unconjugated bilirubin					

MDR-TB TREATMENT INFORMATION

**Starting date:**		**Regimen:** ☐ IVA ☐ IVB ☐ Other			
MDR TB drugs	**Dosage**	**Frequency**	**Other drugs**	**Dosage**	**Frequency**
FOLLOW-UP					
FOLLOW-UP					
FOLLOW-UP					
FOLLOW-UP					
FOLLOW-UP					
FOLLOW-UP					
FOLLOW-UP					
**Reporting date:**		**Reporter:**		**Phone number:**	

FORM 2 Patient's code:

:

FORM FOR COLLECTION SUSPECTED ADVERSE DRUG REACTION (ADR) INFORMATION

MDR-TBfacility:_____________________ District :____

Patient's name:____________________________________<br>Sex: ☐ Male ☐ Female Weight:________kg<br>Date of birth:_____/_____/________<br>Register number:_______________________________<br>ID number:____	Month:____<br>Date: _____/_______/20___________<br>Patient situation:<br>☐ Inpatient<br>☐ Monthly re-examination<br>☐ Unsheduled re-examination
Monthly re-examination:	☐ No ADR<br>☐ ADR happened

1. Treatment change

1.1 Change the regimen: ☐ Yes ☐ No

(If No, go to 1.4)

1.2. If Yes:

Changed drug	New drugs
1.	1.
2.	2.
3.	3.
4.	4.

1.3. Reasons:

☐ Having ADE ☐ Poor compliance ☐ Run out of drug

☐ Treatment failure ☐ Prenancy ☐ Serious diseases

☐ Other

____

1.4. Using other drugs: ☐ Yes ☐ No

If Yes:____

____ ______________________

2. ADE

***Conventional codes** (Check the codes and fill the blanks)*

Seriousness	ADR treatment	
0 = Not serious<br>1 = Death<br>2 = Life threatening<br>3 = Requires or prolongs hospitalization<br>4 = Disability or permanent damage<br>5 = Congenital anomaly/ birth defect<br>6 = No information	0= No treatment<br>1 = Reducing dose<br>2 = Stopping drug<br>3 = Changing regimen<br>4 = Drug to treat ADR *(please specify)* :<br>*41 = Histamin antagonist (please specify)*<br>*42 = Coticoid (please specify)*<br>*43 = Adrenalin*<br>*44* = Other, please specify	5 Renal dialysis<br>6 Transfer from another facility<br>7 No information<br>99 Other _________________<br>______________________

kop[=

	ADE	Appearance	Date	Seriousness	Treatment
**1**	**Nausea, vomiting**	□			
**2**	***Diarrhoea***	□			
**3**	***Abdominal pain***	□			
**4**	***Anorexia***	□			
**5**	***Gastritis*** *(Multichoice)*	□			
	1. Heartburn	□			
	2. Epigastric pain	□			
	3. Results gastroscopy (specify results)	□			
**6**	***Hepatotoxicity*** *(Multichoice)*	□			
	1. Jaundice, yellow eyes	□			
	2. Dark urine	□			
	3. Pale stools	□			
	4. The right upper quadrant abdominal pain	□			
	5. Itchy	□			
	6. ASAT, ALAT increasing<br>ASAT =_________________(U/l)<br>ALAT =	□			
	7. Bilirubin increasing<br>Total Bilirubin = (µmol/l)<br>Unconjugated Bilirubin = ________(µmol/l)	□			
**7**	***Headache***	□			
**8**	***Dizziness, vertigo***	□			
**9**	***Seizures, epilepsy***	□			
**10**	***Psychosis*** *(Multichoice)*	□			
	1. Hallucinations, auditory hallucinations	□			
	2. Confusing thoughts	□			
	3. Odd behavior	□			
	4. Depression	□			
	5. Insomnia	□			
	6. Losing focus	□			
	7. Having suicidal	□			
**11**	***Peripheral neuropathy***	□			
	Finger and toe numbness	□			
**12**	***Arthralgia***	□			
**13**	***Allergic reaction*** *(Multichoice)*	□			
	1. Rash	□			
	2. Itchy	□			
	3. Hypersensitive skin with light	□			

	Biến cố bất lợi	Appearance	Date	Serious	Treatment
**14**	***Nephrotoxic*** *(Multichoice)*	☐			
	1. Retention of urine	☐			
	2. Oedema	☐			
	3. Creatinine increasing (specific result)<br>Creatinine = (µmol/l)	☐			
	4. Urea increasing<br>Urea = _(mmol/l)	☐			
**15**	***Vestibular - auditory disorders*** *(Multichoice)*	☐			
	1. Blurred hearing/ deaf	☐			
	2. Vestibular Disorders	☐			
**16**	***Visual disturbances*** *(Multichoice)*	☐			
	3. Blurred vision	☐			
	4. Dificulty in distinguishing colors	☐			
**17**	***Hypothyroidism*** *(Multichoice)*	☐			
	1. Thyroid hypertrophy	☐			
	2. Constipation	☐			
	3. Weight gain	☐			
	4. Dry skin, dry hair	☐			
	5. Intolerant of cold	☐			
	6. Husky speaking	☐			
	7. Slow pulse	☐			
	8. Menstrual Disorders (in women), sexual disorders (in men)	☐			
	9. TSH increasing (*specific result*)<br>TSH = _(µU/l)	☐			
	10. T3, T4 decreasing (*specific result*)<br>T3 = _____________(nmol/l)<br>T4 = ___________________(nmol/l)	☐			
**18**	***Potassium decreasing*** *(specific result)*<br>K+ = _(mmol/l)	☐			
**19**	***Uric acid increasing*** *(specific result)*<br>Uric Acid = ________________________(µmol/l)	☐			
**20**	***Rối loạn huyết học*** *(Multichoice)*	☐			
	1. Anemia (*specific result)*<br>Hb = _(g/dl)	☐			
	2. Neutropenia (*specific result)*<br>WBC = (x 10^9/l)	☐			
	3. Thrombocytopenia (*specific result)*<br>PLT = __________ (x 10^9/l)	☐			
**21**	***Endocrine disorders*** *(Multichoice)*	☐			

	Biến cố bất lợi	Appearance	Date	Serious	Treatment
	1. Breast enlargement (in males)	□			
	2. Physiological dysfunction (in men)	□			
	3. Menstrual Disorders (in women)	□			
**22**	***Reactions at the injection site*** *(Multichoice)*	□			
	1. Pain	□			
	2. Swollen	□			
	3. Callosity	□			
	4. Itchy	□			
**23**	***Anaphylaxis*** *(Multichoice)*	□			
	1. On skin *(rash,itchy and vasodilation...)*	□			
	2. On répiratory *(Dyspnea, tracheal constriction...)*	□			
	3. Hypotension	□			
	4. On gastrointestinal (stomach cramps, diarrhea	□			
**99**	***Other***	□			

**Date:**<br><br>____/______/ 20__________	**Signature:**	**Phone number:**
