# Supplementary material for: Active surveillance for adverse events in patients on longer treatment regimens for multidrug-resistant tuberculosis in Viet Nam
Source: PLoS One. 2021 Sep 7;16(9):e0255357. doi: 10.1371/journal.pone.0255357 (PMC8423256; doi:10.1371/journal.pone.0255357)
Supplement: S3 File — (ZIP) [file pone.0255357.s006.zip › Additional File/Syntax SPSS.docx]

* Encoding: UTF-8.

***Chuyen dau phay thanh dau cham neu nhap lieu bi loi***

compute hongcau0 = replace ( hongcau0 ,",",".").

EXECUTE.

compute hmb0 = replace ( hmb0 ,",",".").

EXECUTE.

compute bc0 = replace ( bc0 ,",",".").

EXECUTE.

compute tc0 = replace ( tc0 ,",",".").

EXECUTE.

compute alt0 = replace ( alt0 ,",",".").

EXECUTE.

compute ast0 = replace ( ast0 ,",",".").

EXECUTE.

compute biltp0 = replace ( biltp0 ,",",".").

EXECUTE.

compute biltd0 = replace ( biltd0 ,",",".").

EXECUTE.

compute kali0 = replace ( kali0 ,",",".").

EXECUTE.

compute mg0 = replace ( mg0 ,",",".").

EXECUTE.

compute uric0 = replace ( uric0 ,",",".").

EXECUTE.

compute cr0 = replace ( cr0 ,",",".").

EXECUTE.

compute ure0 = replace ( ure0 ,",",".").

EXECUTE.

compute tsh0 = replace ( tsh0 ,",",".").

EXECUTE.

*** chuyen string thanh numeric***

ALTER TYPE hongcau0 (f5) .

ALTER TYPE hmb0 (f5) .

ALTER TYPE bc0 (f5) .

ALTER TYPE tc0 (f5) .

ALTER TYPE alt0 (f5) .

ALTER TYPE ast0 (f5) .

ALTER TYPE biltp0 (f5) .

ALTER TYPE biltd0 (f5) .

ALTER TYPE kali0 (f5) .

ALTER TYPE mg0 (f5) .

ALTER TYPE uric0 (f5) .

ALTER TYPE cr0 (f5) .

ALTER TYPE ure0 (f5) .

ALTER TYPE tsh0 (f5) .

****biến đổi các mã dac diem benh nhan trong nghien cuu****

Recode gioi (CONVERT) ('1=nam'=1) ('2=nu'=0) (else=99) (MISSING=sysmis) INTO gioia.

execute.

RECODE gioia(MISSING=99).

Value labels gioia

1 'Nam' 0 'Nu' 99 'missing'.

execute.

Recode donvi (CONVERT) ('Bình Định'=1) ('Bình ĐỊnh'=1) ('Bình Thuận'=2) ('BV 74'=3) ('BV 74 trung ương'=3) ('BV 74 Trung ương'=3) ('BV 74 Trung Ương'=3) ('BV 74 TW'=3)

('Bv lao phổi BT'=2) ('BV lao phổi BT'=2) ('Bv lao QN'=4) ('BV lao QN'=4) ('Bv phổi TH'=5) ('BV phổi TH'=5) ('BV phổi Thanh Hóa'=5) ('BV Phổi Thanh Hóa'=5) ('BV74 Trung ương'=3)

('Cần Thơ'=6) ('ha noi'=7) ('HN'=7) ('hoc mon, TPHCM'=8) ('Nam Định'=9) ('QN'=4) ('Quảng Nam'=4) ('TH'=5) ('Thanh Hóa'=5) ('Tp HCM'=8) ('TP HCM'=8)

('TPHCM'=8) (else=99) (missing=sysmis) INTO donvia.

execute.

RECODE donvia(MISSING=99).

Value labels donvia

1 'Binh Dinh' 2 'Binh Thuan' 3 '74 Trung Uong' 4 'Quang Nam' 5 'Thanh Hoa' 6 'Can Tho' 7 'Ha Noi' 8 'TPHCM' 9 'Nam Dinh' 99 'missing'.

execute.

Recode loaibn (CONVERT) ('1=moi'=1) ('2=tai phat'=2) ('3=that bai I'=3) ('4=that bai II'=4) ('5=sau bo tri'=5) ('6=chuyen den'=6) ('99='=99) ('99= sau dieu tri'=99) ('99=BN tiếp xúc BN lao kháng thuốc AFB+'=99) ('99=chưa àm hóa PĐ II sau 3 tháng'=99)

('99=chưa âm hóa phác đồ I'=99) ('99=ĐIỀU TRỊ 2 LẦN TẠI TRẠI GIAM, KHÔNG BIẾN CỐ'=99) ('99=khac'=99) ('99=khác'=99) ('99=lao cot song'=99) ('99=nghi lao có tiền sử điều trị >1 tháng'=99)

('99=Nghi lao có tiền sử điều trị trên 1 tháng'=99) ('99=nghi lao có tiền sử điều trị>1 thnags'=99) ('99=nghi lao có tiền sử> 1 tháng'=99) ('99=nghi lao có tiền sử> 1thang'=99) ('99=nghi lao có tiền sử>1 tháng'=99)

('99=nghi ngờ lao phổi âm tính'=99) ('99=người nghi lao có tiền sử điều trị> 1 tháng'=99) ('99=người ngi lao có tiền sử đt>1tháng'=99) ('99=T/s dieu tri thuoc H2'=99) (else=99) (MISSING=sysmis) INTO loaibna.

execute.

recode loaibna(MISSING=99).

Value labels loaibna

1 'moi' 2 'taiphat' 3 'that bai I' 4 'that bai II' 5 'sau bo tri' 6 'chuyen den' 99 'missing'.

execute.

do if (loaibna=2 or loaibna=5).

compute dieutrilao=1.

else.

compute dieutrilao=0.

end if.

Recode vitri (CONVERT) ('1=phoi'=1) ('2=ngoai phoi'=2) ('3=ca hai'=3) (else=99) (missing=sysmis) INTO vitria.

execute.

recode vitria(MISSING=99).

Value labels vitria

1 'phoi' 2 'ngoai phoi' 3 'ca hai' 99 'missing'.

execute.

recode nang(MISSING=0).

create leadnang=lead(nang,1).

Do IF ($CASENUM=1).

compute nanga=nang.

else if nang<>0.

compute nanga=nang.

else if (Table1x.ID = LAG(Table1x.ID)) AND nang=0.

compute nanga=lag(nanga).

else if (Table1x.ID <> LAG(Table1x.ID)) AND nang=0.

compute nanga=leadnang.

end if.

***Bien doi benh mac kem***

Recode mackem1 (CONVERT) ('0=khong co BMK'=0) ('10=benh co xuong khop '=10) ('2=roi loan tam than'=2) ('3=benh mau'=3) ('4=dai thao duong'=4) ('5=suy than'=5) ('6=benh gan'=6) ('7=benh tieu hoa'=7) ('8=benh thi giac'=8)

('9=benh thinh giac'=9) ('11=benh he mien dich'=11) ('99= benh basedow'=2) ('99= Cắt 1/2 phổi trái'=12) ('99= COPD'=12) ('99= gian phe quan'=12) ('99= hen phe quan'=12) ('99= hen suyen'=12)

('99= lao hạch, cột sống'=12) ('99= tim mach'=13) ('99= tim mạch'=13) ('99= u thanh quan'=99) ('99=AIDS'=11) ('99=benh phoi tac nghen man tinh'=12) ('99=benh suyen'=12)

('99=bội nhiễm phổi '=12) ('99=Bội nhiễm phổi'=12) ('99=buou lanh tien liet tuyen,nhiem trung tieu'=99)

('99=cao huyet ap'=14) ('99=ECG dây nhĩ phải'=13) ('99=gian phe quan'=12) ('99=gout'=99) ('99=Gout'=99) ('99=hep khi quan'=12) ('99=hoi chung than hu cach day 8 nam'=5) ('99=huyet khoi tinh mach'=13)

('99=LMN'=99) ('99=phong'=99) ('99=tac dong mach chi duoi'=99) ('99=tang huyet ap'=14) ('99=than u nuoc do II'=5) ('99=theo dõi phổi'=12) ('99=thieu mau'=3) ('99=Thiếu máu cơ tim'=13) ('99=tran khi mang phoi'=12)

('99=viem tai giua ben tai trai'=9) ('HIV'=11) ('không có BMk'=0) ('không có BMK'=0) ('Không có BMK'=0) (else=99) (MISSING=sysmis) INTO mackem1a.

execute.

recode mackem1a (missing=99).

Value labels mackem1a

0 'khong co BMK' 10 'benh co xuong khop' 2 'roi loan tam than' 3 'benh mau' 4 'dai thao duong' 5 'suy than' 6 'benh gan' 7 'benh tieu hoa' 8 'benh thi giac' 9 'benh thinh giac'

11 'benh he mien dich' 12 'benh ho hap' 13 'benh tim mach' 14 'tang huyet ap' 99 'missing'.

execute.

recode mackem2 (convert) ('5=suy than'=5) ('6=benh gan'=6) ('9=benh thinh giac'=9) ('99='=99) ('99= benh phoi tac nghen man tinh'=12)

('99= huyet ap'=14) ('99= huyet ap cao'=14) ('99= parkinson'=2) ('99= tang huyet ap'=14) ('99=benh tim mach'=13) ('99=cao huyet ap'=14) ('99=Cao huyết áp'=14)

('99=HBsAg dương tính'=6) ('99=huyet ap'=14) ('99=tang huyet ap'=14) ('99=TKMP'=12) ('99=ung thư amidal'=99) (else=99) (missing=sysmis) into mackem2a.

EXECUTE.

recode mackem2a(missing=99).

EXECUTE.

VALUE LABELS mackem2a

0 'khong co BMK' 10 'benh co xuong khop' 2 'roi loan tam than' 3 'benh mau' 4 'dai thao duong' 5 'suy than' 6 'benh gan' 7 'benh tieu hoa' 8 'benh thi giac' 9 'benh thinh giac'

11 'benh he mien dich' 12 'benh ho hap' 13 'benh tim mach' 14 'tanghuyetap' 99 'missing'.

execute.

recode mackem3 (convert) ('99=cao huyet ap'=14) ('99=Cao huyết áp'=14) ('99=teo nao nguoi gia'=99) ('99=than u nuoc do III'=5) (else=99) (missing=sysmis) into mackem3a.

EXECUTE.

recode mackem3a(missing=99).

EXECUTE.

VALUE LABELS mackem3a

0 'khong co BMK' 10 'benh co xuong khop' 2 'roi loan tam than' 3 'benh mau' 4 'dai thao duong' 5 'suy than' 6 'benh gan' 7 'benh tieu hoa' 8 'benh thi giac' 9 'benh thinh giac'

11 'benh he mien dich' 12 'benh ho hap' 13 'benh tim mach' 14 'tanghuyetap' 99 'missing'.

execute.

do if (mackem1a=8 or mackem2a=8 or mackem3a=8).

compute mackemthigiac=1.

else.

compute mackemthigiac=0.

end if.

do if (mackem1a=13 or mackem2a=13 or mackem3a=13).

compute mackemtm=1.

else.

compute mackemtm=0.

end if.

do if (mackem1a=12 or mackem2a=12 or mackem3a=12).

compute mackemhh=1.

else.

compute mackemhh=0.

end if.

do if (mackem1a=14 or mackem2a=14 or mackem3a=14).

compute mackemtha=1.

else.

compute mackemtha=0.

end if.

do if (mackem1a=4 or mackem2a=4 or mackem3a=4).

compute mackemdaithaoduong=1.

else.

compute mackemdaithaoduong=0.

end if.

do if (mackem1a=6 or mackem2a=6 or mackem3a=6).

compute mackembenhgan=1.

else.

compute mackembenhgan=0.

end if.

do if (mackem1a=7 or mackem2a=7 or mackem3a=7).

compute mackembenhduongtieuhoa=1.

else.

compute mackembenhduongtieuhoa=0.

end if.

do if (mackem1a=9 or mackem2a=9 or mackem3a=9).

compute mackembenhthinhgiac=1.

else.

compute mackembenhthinhgiac=0.

end if.

do if (mackem1a=10 or mackem2a=10 or mackem3a=10).

compute mackembenhcoxuongkhop=1.

else.

compute mackembenhcoxuongkhop=0.

end if.

do if (mackem1a=5 or mackem2a=5 or mackem3a=5).

compute mackembenhsuythan=1.

else.

compute mackembenhsuythan=0.

end if.

do if (mackem1a=11 or mackem2a=11 or mackem3a=11).

compute mackembenhhemiendich=1.

else.

compute mackembenhhemiendich=0.

end if.

do if (mackem1a=2 or mackem2a=2 or mackem3a=2).

compute mackembenhroiloantamthan=1.

else.

compute mackembenhroiloantamthan=0.

end if.

do if (mackem1a=3 or mackem2a=3 or mackem3a=3).

compute mackembenhmau=1.

else.

compute mackembenhmau=0.

end if.

***Bien doi tinh trang benh nhan***

recode tinhtrang1 (convert) ('2=cho con bu'=2) ('3=nghien ruou'=3) ('4=nghien ma tuy'=4) ('5=suy kiet'=5) ('99='=99) ('99= ho ra máu'=99) ('99=co rut'=99)

('99=dan luu mu mang phoi phai'=99) ('99=không có tình trạng bất thường'=99) ('99=yeu nua nguoi'=99) (else=99) (missing=sysmis) into tinhtrang1a.

EXECUTE.

recode tinhtrang1a (missing=99).

EXECUTE.

VALUE LABELS tinhtrang1a

2 'cho con bu' 3 'nghien ruou' 4 'nghien ma tuy' 5 'suy kiet' 99 'missing'.

EXECUTE.

recode tinhtrang2 (convert) ('5=suy kiet'=5) ('99= suy ho hap'=99) ('99=hau san mo'=99) ('99=suy ho hap'=99) (else=99) (missing=sysmis) into tinhtrang2a.

EXECUTE.

recode tinhtrang2a (missing=99).

EXECUTE.

VALUE LABELS tinhtrang2a

2 'cho con bu' 3 'nghien ruou' 4 'nghien ma tuy' 5 'suy kiet' 99 'missing'.

EXECUTE.

do if (tinhtrang1a=5 or tinhtrang2a=5).

compute tinhtrangsuykiet=1.

else.

compute tinhtrangsuykiet=0.

end if.

do if (tinhtrang1a=4 or tinhtrang2a=4).

compute tinhtrangnghienmatuy=1.

else.

compute tinhtrangnghienmatuy=0.

end if.

do if (tinhtrang1a=3 or tinhtrang2a=3).

compute tinhtrangnghienruou=1.

else.

compute tinhtrangnghienruou=0.

end if.

do if (tinhtrang1a=2 or tinhtrang2a=2).

compute tinhtrangchoconbu=1.

else.

compute tinhtrangchoconbu=0.

end if.

***Bien doi tinh trang HIV***

Recode hiv (CONVERT) ('1=HIV + dieu tri ARV'=1) ('2=HIV chua dieu tri'=2) ('3=HIV am tinh'=3) ('4=khong ro'=4) ('âm tính'=3) (else=99) (missing=sysmis) INTO hiva.

execute.

recode hiva (missing=99).

Value labels hiva

1 'HIV + dieu tri ARV' 2 'HIV chua dieu tri' 3 'HIV am tinh' 4 'khong ro' 99 'missing'.

execute.

do if (hiva=1 or hiva=2).

compute tinhtrangnhiemhiv=1.

else.

compute tinhtrangnhiemhiv=0.

end if.

***Bien doi phac do***

Recode phacdo (CONVERT) ('1=phac do Iva'=1) ('1=phac do IVa'=1) ('2=phac do Ivb'=2) ('2=phac do IVb'=2) ('99=phac do ca nhan hoa'=3) (ELSE=99) (missing=sysmis) INTO phacdoa.

execute.

RECODE phacdoa (missing=99).

EXECUTE.

Value labels phacdoa

1 'Phac do IVa' 2 'Phac do IVb' 3 'Phac do ca nhan hoa' 99 'missing'.

execute.

*** Tính tuoi benh nhan***

compute tuoi=xdate.year(ngaydau) - namsinh.

ALTER TYPE tuoi(f5).

execute.

***Bien doi thoi gian theo doi cua benh nhan***

SORT CASES BY Table1x.ID(A) ngay(A).

COMPUTE tte=(ngay-ngaydau)/86400.

EXECUTE.

FILTER OFF.

USE ALL.

SELECT IF (tte>=0 OR tte<1000).

EXECUTE.

do if (Table1x.ID=lag(Table1x.ID)).

compute interval=tte-lag(tte).

else.

compute interval=0.

end if.

***Bien doi tinh trang ket thuc dieu tri MDR-TB cua benh nhan***

recode ma1 (CONVERT) ('1 = dang dieu tri'=1) ('1= dang dieu tri'=1) ('2 = ngung thuoc'=2) ('3 = hoan thanh'=3) ('4= tu vong'=4) ('5 = chuyen di'=5) ('6= bo tri'=6)

('99=that bai'=7) ('99=that bai dieu tri'=7) (else=99) (missing=sysmis) INTO ma1a.

execute.

recode ma1a (missing=99).

EXECUTE.

value labels ma1a

1 'Dang dieu tri' 2 'Ngung thuoc' 3 'Hoan thanh' 4 'Tu vong' 5 'Chuyen di' 6 'Bo tri' 7 'That bai dieu tri' 99 'missing'.

execute.

***So luot BN va thoi gian tu vong***

do if (ma1a=4).

compute BNtuvong=1.

else.

compute BNtuvong=0.

end if.

Do if ((Table1x.ID = LAG(Table1x.ID)) AND BNtuvong=1).

compute BNtuvongkp=BNtuvong.

ELSE IF (Table1x.ID=lag(Table1x.ID) AND BNtuvong=0).

COMPUTE BNtuvongkp=lag(BNtuvongkp).

ELSE.

Compute BNtuvongkp=BNtuvong.

END IF.

do if (sysmis(BNtuvongkp)).

compute BNtuvongkp=0.

else.

compute BNtuvongkp=BNtuvongkp.

end if.

Do if ((Table1x.ID=lag(Table1x.ID)) and (BNtuvongkp=0)).

compute ttuvong=tte.

else if (Table1x.ID=lag(Table1x.ID) and BNtuvongkp=1 and lag(BNtuvongkp)=0).

compute ttuvong=tte.

else if (Table1x.ID=lag(Table1x.ID) and BNtuvongkp=1 and lag(BNtuvongkp)=1).

compute ttuvong=lag(ttuvong).

ELSE.

compute ttuvong=tte.

end if.

do if (sysmis(ttuvong)).

compute ttuvong=tte.

else.

compute ttuvong=ttuvong.

end if.

***So luot BN va thoi gian chuyen di***

do if (ma1a=5).

compute BNchuyendi=1.

else.

compute BNchuyendi=0.

end if.

Do if ((Table1x.ID = LAG(Table1x.ID)) AND BNchuyendi=1).

compute BNchuyendikp=BNchuyendi.

ELSE IF (Table1x.ID=lag(Table1x.ID) AND BNchuyendi=0).

COMPUTE BNchuyendikp=lag(BNchuyendikp).

ELSE.

Compute BNchuyendikp=BNchuyendi.

END IF.

do if (sysmis(BNchuyendikp)).

compute BNchuyendikp=0.

else.

compute BNchuyendikp=BNchuyendikp.

end if.

Do if ((Table1x.ID=lag(Table1x.ID)) and (BNchuyendikp=0)).

compute tchuyendi=tte.

else if (Table1x.ID=lag(Table1x.ID) and BNchuyendikp=1 and lag(BNchuyendikp)=0).

compute tchuyendi=tte.

else if (Table1x.ID=lag(Table1x.ID) and BNchuyendikp=1 and lag(BNchuyendikp)=1).

compute tchuyendi=lag(tchuyendi).

ELSE.

compute tchuyendi=tte.

end if.

do if (sysmis(tchuyendi)).

compute tchuyendi=tte.

else.

compute tchuyendi=tchuyendi.

end if.

***So luot BN va thoi gian bo tri***

Do if (ma1a=6).

compute BNbotri=1.

ELSE.

Compute BNbotri=0.

END IF.

Do if ((Table1x.ID = LAG(Table1x.ID)) AND BNbotri=1).

compute BNbotrikp=BNbotri.

ELSE IF (Table1x.ID=lag(Table1x.ID) AND BNbotri=0).

COMPUTE BNbotrikp=lag(BNbotrikp).

ELSE.

Compute BNbotrikp=BNbotri.

END IF.

do if (sysmis(BNbotrikp)).

compute BNbotrikp=0.

else.

compute BNbotrikp=BNbotrikp.

end if.

Do if ((Table1x.ID=lag(Table1x.ID)) and (BNbotrikp=0)).

compute tbotri=tte.

else if (Table1x.ID=lag(Table1x.ID) and BNbotrikp=1 and lag(BNbotrikp)=0).

compute tbotri=tte.

else if (Table1x.ID=lag(Table1x.ID) and BNbotrikp=1 and lag(BNbotrikp)=1).

compute tbotri=lag(tbotri).

ELSE.

compute tbotri=tte.

end if.

do if (sysmis(tbotri)).

compute tbotri=tte.

else.

compute tbotri=tbotri.

end if.

***So luot BN va thoi gian that bai dieu tri***

Do if (ma1a=7).

compute BNthatbai=1.

ELSE.

Compute BNthatbai=0.

END IF.

Do if ((Table1x.ID = LAG(Table1x.ID)) AND BNthatbai=1).

compute BNthatbaikp=BNthatbai.

ELSE IF (Table1x.ID=lag(Table1x.ID) AND BNthatbai=0).

COMPUTE BNthatbaikp=lag(BNthatbaikp).

ELSE.

Compute BNthatbaikp=BNthatbai.

END IF.

do if (sysmis(BNthatbaikp)).

compute BNthatbaikp=0.

else.

compute BNthatbaikp=BNthatbaikp.

end if.

Do if ((Table1x.ID=lag(Table1x.ID)) and (BNthatbaikp=0)).

compute tthatbai=tte.

else if (Table1x.ID=lag(Table1x.ID) and BNthatbaikp=1 and lag(BNthatbaikp)=0).

compute tthatbai=tte.

else if (Table1x.ID=lag(Table1x.ID) and BNthatbaikp=1 and lag(BNthatbaikp)=1).

compute tthatbai=lag(tthatbai).

ELSE.

compute tthatbai=tte.

end if.

do if (sysmis(tthatbai)).

compute tthatbai=tte.

else.

compute tthatbai=tthatbai.

end if.

***So luot BN va thoi gian hoan thanh dieu tri***

Do if (ma1a=3).

compute BNhoanthanh=1.

ELSE.

Compute BNhoanthanh=0.

END IF.

Do if ((Table1x.ID = LAG(Table1x.ID)) AND BNhoanthanh=1).

compute BNhoanthanhkp=BNhoanthanh.

ELSE IF (Table1x.ID=lag(Table1x.ID) AND BNhoanthanh=0).

COMPUTE BNhoanthanhkp=lag(BNhoanthanhkp).

ELSE.

Compute BNhoanthanhkp=BNhoanthanh.

END IF.

do if (sysmis(BNhoanthanhkp)).

compute BNhoanthanhkp=0.

else.

compute BNhoanthanhkp=BNhoanthanhkp.

end if.

Do if ((Table1x.ID=lag(Table1x.ID)) and (BNhoanthanhkp=0)).

compute thoanthanh=tte.

else if (Table1x.ID=lag(Table1x.ID) and BNhoanthanhkp=1 and lag(BNhoanthanhkp)=0).

compute thoanthanh=tte.

else if (Table1x.ID=lag(Table1x.ID) and BNhoanthanhkp=1 and lag(BNhoanthanhkp)=1).

compute thoanthanh=lag(thoanthanh).

ELSE.

compute thoanthanh=tte.

end if.

do if (sysmis(thoanthanh)).

compute thoanthanh=tte.

else.

compute thoanthanh=thoanthanh.

end if.

*** Bien doi phac do dieu tri***

Recode thuoc1 (CONVERT) ('amikacin'=1) ('capreomycin'=2) ('ethambutol'=4) ('ethambutol 0,4mg'=4) ('ethambutol 400mg'=4) ('ethambutol 400'=4) ('ethambutol 400MG'=4) ('ethambutol 0,4g'=4)

('Ethambutol 0,4g'=4) ('ethambutol 0.4g'=4) ('kanamycin'=6) ('Kanamycin'=6) ('kanamycin ( tiem cach ngay)'=6) ('kanamycin (tiem cach ngay)'=6) ('kanamycin 1g'=6) ('Kanamycin 1g'=6)

('kanamycin( tiem cach ngay)'=6) ('kanamycin(giam lieu)'=6) ('kanamycin1g'=6) ('levofloxacin'=7) ('levofloxacin 0,25g'=7) ('Levofloxacin 0,25g'=7) ('levofloxacin 0.25g'=7) ('Levofloxacin 250 mg'=7)

('levofloxacin 250mg'=7) ('Levofloxacin 250mg'=7) ('levofloxacin 250 mg'=7) ('levofloxacin 50mg'=7) ('prothionamid'=10) ('prothionamid 0.25g'=10) ('prothionamid 250 mg'=10) ('prothionamid 250mg'=10)

('Prothionamid 250mg'=10) ('Prothionamid 0,25g'=10) ('prothionamid 0,25g'=10) ('Prothionamid'=10) ('prothionamid250mg'=10) ('pyrazinamid'=11) ('Pyrazinamid 0,5g'=11) ('pyrazinamid 0,5g'=11)

('Pyrazinamid'=11) ('pyrazinamid 500 mg'=11) ('pyrazinamid 500mg'=11) ('pyrazinamid 0.5g'=11) ('pyrazinamid 0.5gg'=11) ('pyrazinamid 0,4g'=11) ('pyrazinamid ( giam lieu)'=11) ('pyrazinamid (giam lieu)'=11)

('pyrazinamid 0,5 g'=11) ('linezolid'=13) ('PAS 4g'=9) ('PAS'=9) ('PAP'=9) ('clofazimin'=14) ('cycloserin 250mg'=3) ('Cycloserin 250mg'=3) ('Cycloserin 250 mg'=3) ('cycloserin 0.25g'=3) ('cycloserin 250'=3)

('cycloserin 0,25g'=3) ('Cycloserin 0,25g'=3) ('cycloserin 0.25h'=3) ('Cycloserin'=3) ('cycloserin'=3) ('cycloserin 200mg'=3) ('bedaquiline'=15) ('rifampicin'=12) ('moxifloxacin'=8) ('moxifloxacin'=8) ('isoniazid'=5)

('Ethambutol 0,4 g'=4) (missing=sysmis) INTO thuoc1a.

execute.

recode thuoc1a (MISSING=99).

Execute.

Recode thuoc2 (CONVERT) ('amikacin'=1) ('capreomycin'=2) ('ethambutol'=4) ('ethambutol 0,4mg'=4) ('ethambutol 400mg'=4) ('ethambutol 400'=4) ('ethambutol 400MG'=4) ('ethambutol 0,4g'=4)

('Ethambutol 0,4g'=4) ('ethambutol 0.4g'=4) ('kanamycin'=6) ('Kanamycin'=6) ('kanamycin ( tiem cach ngay)'=6) ('kanamycin (tiem cach ngay)'=6) ('kanamycin 1g'=6) ('Kanamycin 1g'=6)

('kanamycin( tiem cach ngay)'=6) ('kanamycin(giam lieu)'=6) ('kanamycin1g'=6) ('levofloxacin'=7) ('levofloxacin 0,25g'=7) ('Levofloxacin 0,25g'=7) ('levofloxacin 0.25g'=7) ('Levofloxacin 250 mg'=7)

('levofloxacin 250mg'=7) ('Levofloxacin 250mg'=7) ('levofloxacin 250 mg'=7) ('levofloxacin 50mg'=7) ('prothionamid'=10) ('prothionamid 0.25g'=10) ('prothionamid 250 mg'=10) ('prothionamid 250mg'=10)

('Prothionamid 250mg'=10) ('Prothionamid 0,25g'=10) ('prothionamid 0,25g'=10) ('Prothionamid'=10) ('prothionamid250mg'=10) ('pyrazinamid'=11) ('Pyrazinamid 0,5g'=11) ('pyrazinamid 0,5g'=11)

('Pyrazinamid'=11) ('pyrazinamid 500 mg'=11) ('pyrazinamid 500mg'=11) ('pyrazinamid 0.5g'=11) ('pyrazinamid 0.5gg'=11) ('pyrazinamid 0,4g'=11) ('pyrazinamid ( giam lieu)'=11) ('pyrazinamid (giam lieu)'=11)

('pyrazinamid 0,5 g'=11) ('linezolid'=13) ('PAS 4g'=9) ('PAS'=9) ('PAP'=9) ('clofazimin'=14) ('cycloserin 250mg'=3) ('Cycloserin 250mg'=3) ('Cycloserin 250 mg'=3) ('cycloserin 0.25g'=3) ('cycloserin 250'=3)

('cycloserin 0,25g'=3) ('Cycloserin 0,25g'=3) ('cycloserin 0.25h'=3) ('Cycloserin'=3) ('cycloserin'=3) ('cycloserin 200mg'=3) ('bedaquiline'=15) ('rifampicin'=12) ('moxifloxacin'=8) ('moxifloxacin'=8) ('isoniazid'=5)

('Ethambutol 0,4 g'=4) (else=99) (missing=sysmis) INTO thuoc2a.

execute.

recode thuoc2a (MISSING=99).

Execute.

Recode thuoc2 (CONVERT) ('amikacin'=1) ('capreomycin'=2) ('ethambutol'=4) ('ethambutol 0,4mg'=4) ('ethambutol 400mg'=4) ('ethambutol 400'=4) ('ethambutol 400MG'=4) ('ethambutol 0,4g'=4)

('Ethambutol 0,4g'=4) ('ethambutol 0.4g'=4) ('kanamycin'=6) ('Kanamycin'=6) ('kanamycin ( tiem cach ngay)'=6) ('kanamycin (tiem cach ngay)'=6) ('kanamycin 1g'=6) ('Kanamycin 1g'=6)

('kanamycin( tiem cach ngay)'=6) ('kanamycin(giam lieu)'=6) ('kanamycin1g'=6) ('levofloxacin'=7) ('levofloxacin 0,25g'=7) ('Levofloxacin 0,25g'=7) ('levofloxacin 0.25g'=7) ('Levofloxacin 250 mg'=7)

('levofloxacin 250mg'=7) ('Levofloxacin 250mg'=7) ('levofloxacin 250 mg'=7) ('levofloxacin 50mg'=7) ('prothionamid'=10) ('prothionamid 0.25g'=10) ('prothionamid 250 mg'=10) ('prothionamid 250mg'=10)

('Prothionamid 250mg'=10) ('Prothionamid 0,25g'=10) ('prothionamid 0,25g'=10) ('Prothionamid'=10) ('prothionamid250mg'=10) ('pyrazinamid'=11) ('Pyrazinamid 0,5g'=11) ('pyrazinamid 0,5g'=11)

('Pyrazinamid'=11) ('pyrazinamid 500 mg'=11) ('pyrazinamid 500mg'=11) ('pyrazinamid 0.5g'=11) ('pyrazinamid 0.5gg'=11) ('pyrazinamid 0,4g'=11) ('pyrazinamid ( giam lieu)'=11) ('pyrazinamid (giam lieu)'=11)

('pyrazinamid 0,5 g'=11) ('linezolid'=13) ('PAS 4g'=9) ('PAS'=9) ('PAP'=9) ('clofazimin'=14) ('cycloserin 250mg'=3) ('Cycloserin 250mg'=3) ('Cycloserin 250 mg'=3) ('cycloserin 0.25g'=3) ('cycloserin 250'=3)

('cycloserin 0,25g'=3) ('Cycloserin 0,25g'=3) ('cycloserin 0.25h'=3) ('Cycloserin'=3) ('cycloserin'=3) ('cycloserin 200mg'=3) ('bedaquiline'=15) ('rifampicin'=12) ('moxifloxacin'=8) ('moxifloxacin'=8) ('isoniazid'=5)

('Ethambutol 0,4 g'=4) (missing=sysmis) INTO thuoc2a.

execute.

recode thuoc2a (MISSING=99).

Execute.

Recode thuoc3 (CONVERT) ('amikacin'=1) ('capreomycin'=2) ('ethambutol'=4) ('ethambutol 0,4mg'=4) ('ethambutol 400mg'=4) ('ethambutol 400'=4) ('ethambutol 400MG'=4) ('ethambutol 0,4g'=4)

('Ethambutol 0,4g'=4) ('ethambutol 0.4g'=4) ('kanamycin'=6) ('Kanamycin'=6) ('kanamycin ( tiem cach ngay)'=6) ('kanamycin (tiem cach ngay)'=6) ('kanamycin 1g'=6) ('Kanamycin 1g'=6)

('kanamycin( tiem cach ngay)'=6) ('kanamycin(giam lieu)'=6) ('kanamycin1g'=6) ('levofloxacin'=7) ('levofloxacin 0,25g'=7) ('Levofloxacin 0,25g'=7) ('levofloxacin 0.25g'=7) ('Levofloxacin 250 mg'=7)

('levofloxacin 250mg'=7) ('Levofloxacin 250mg'=7) ('levofloxacin 250 mg'=7) ('levofloxacin 50mg'=7) ('prothionamid'=10) ('prothionamid 0.25g'=10) ('prothionamid 250 mg'=10) ('prothionamid 250mg'=10)

('Prothionamid 250mg'=10) ('Prothionamid 0,25g'=10) ('prothionamid 0,25g'=10) ('Prothionamid'=10) ('prothionamid250mg'=10) ('pyrazinamid'=11) ('Pyrazinamid 0,5g'=11) ('pyrazinamid 0,5g'=11)

('Pyrazinamid'=11) ('pyrazinamid 500 mg'=11) ('pyrazinamid 500mg'=11) ('pyrazinamid 0.5g'=11) ('pyrazinamid 0.5gg'=11) ('pyrazinamid 0,4g'=11) ('pyrazinamid ( giam lieu)'=11) ('pyrazinamid (giam lieu)'=11)

('pyrazinamid 0,5 g'=11) ('linezolid'=13) ('PAS 4g'=9) ('PAS'=9) ('PAP'=9) ('clofazimin'=14) ('cycloserin 250mg'=3) ('Cycloserin 250mg'=3) ('Cycloserin 250 mg'=3) ('cycloserin 0.25g'=3) ('cycloserin 250'=3)

('cycloserin 0,25g'=3) ('Cycloserin 0,25g'=3) ('cycloserin 0.25h'=3) ('Cycloserin'=3) ('cycloserin'=3) ('cycloserin 200mg'=3) ('bedaquiline'=15) ('rifampicin'=12) ('moxifloxacin'=8) ('moxifloxacin'=8) ('isoniazid'=5)

('Ethambutol 0,4 g'=4) (else=99) (missing=sysmis) INTO thuoc3a.

execute.

recode thuoc3a (MISSING=99).

Execute.

Recode thuoc4 (CONVERT) ('amikacin'=1) ('capreomycin'=2) ('ethambutol'=4) ('ethambutol 0,4mg'=4) ('ethambutol 400mg'=4) ('ethambutol 400'=4) ('ethambutol 400MG'=4) ('ethambutol 0,4g'=4)

('Ethambutol 0,4g'=4) ('ethambutol 0.4g'=4) ('kanamycin'=6) ('Kanamycin'=6) ('kanamycin ( tiem cach ngay)'=6) ('kanamycin (tiem cach ngay)'=6) ('kanamycin 1g'=6) ('Kanamycin 1g'=6)

('kanamycin( tiem cach ngay)'=6) ('kanamycin(giam lieu)'=6) ('kanamycin1g'=6) ('levofloxacin'=7) ('levofloxacin 0,25g'=7) ('Levofloxacin 0,25g'=7) ('levofloxacin 0.25g'=7) ('Levofloxacin 250 mg'=7)

('levofloxacin 250mg'=7) ('Levofloxacin 250mg'=7) ('levofloxacin 250 mg'=7) ('levofloxacin 50mg'=7) ('prothionamid'=10) ('prothionamid 0.25g'=10) ('prothionamid 250 mg'=10) ('prothionamid 250mg'=10)

('Prothionamid 250mg'=10) ('Prothionamid 0,25g'=10) ('prothionamid 0,25g'=10) ('Prothionamid'=10) ('prothionamid250mg'=10) ('pyrazinamid'=11) ('Pyrazinamid 0,5g'=11) ('pyrazinamid 0,5g'=11)

('Pyrazinamid'=11) ('pyrazinamid 500 mg'=11) ('pyrazinamid 500mg'=11) ('pyrazinamid 0.5g'=11) ('pyrazinamid 0.5gg'=11) ('pyrazinamid 0,4g'=11) ('pyrazinamid ( giam lieu)'=11) ('pyrazinamid (giam lieu)'=11)

('pyrazinamid 0,5 g'=11) ('linezolid'=13) ('PAS 4g'=9) ('PAS'=9) ('PAP'=9) ('clofazimin'=14) ('cycloserin 250mg'=3) ('Cycloserin 250mg'=3) ('Cycloserin 250 mg'=3) ('cycloserin 0.25g'=3) ('cycloserin 250'=3)

('cycloserin 0,25g'=3) ('Cycloserin 0,25g'=3) ('cycloserin 0.25h'=3) ('Cycloserin'=3) ('cycloserin'=3) ('cycloserin 200mg'=3) ('bedaquiline'=15) ('rifampicin'=12) ('moxifloxacin'=8) ('moxifloxacin'=8) ('isoniazid'=5)

('Ethambutol 0,4 g'=4) (else=99) (missing=sysmis) INTO thuoc4a.

execute.

Recode thuoc5 (CONVERT) ('amikacin'=1) ('capreomycin'=2) ('ethambutol'=4) ('ethambutol 0,4mg'=4) ('ethambutol 400mg'=4) ('ethambutol 400'=4) ('ethambutol 400MG'=4) ('ethambutol 0,4g'=4)

('Ethambutol 0,4g'=4) ('ethambutol 0.4g'=4) ('kanamycin'=6) ('Kanamycin'=6) ('kanamycin ( tiem cach ngay)'=6) ('kanamycin (tiem cach ngay)'=6) ('kanamycin 1g'=6) ('Kanamycin 1g'=6)

('kanamycin( tiem cach ngay)'=6) ('kanamycin(giam lieu)'=6) ('kanamycin1g'=6) ('levofloxacin'=7) ('levofloxacin 0,25g'=7) ('Levofloxacin 0,25g'=7) ('levofloxacin 0.25g'=7) ('Levofloxacin 250 mg'=7)

('levofloxacin 250mg'=7) ('Levofloxacin 250mg'=7) ('levofloxacin 250 mg'=7) ('levofloxacin 50mg'=7) ('prothionamid'=10) ('prothionamid 0.25g'=10) ('prothionamid 250 mg'=10) ('prothionamid 250mg'=10)

('Prothionamid 250mg'=10) ('Prothionamid 0,25g'=10) ('prothionamid 0,25g'=10) ('Prothionamid'=10) ('prothionamid250mg'=10) ('pyrazinamid'=11) ('Pyrazinamid 0,5g'=11) ('pyrazinamid 0,5g'=11)

('Pyrazinamid'=11) ('pyrazinamid 500 mg'=11) ('pyrazinamid 500mg'=11) ('pyrazinamid 0.5g'=11) ('pyrazinamid 0.5gg'=11) ('pyrazinamid 0,4g'=11) ('pyrazinamid ( giam lieu)'=11) ('pyrazinamid (giam lieu)'=11)

('pyrazinamid 0,5 g'=11) ('linezolid'=13) ('PAS 4g'=9) ('PAS'=9) ('PAP'=9) ('clofazimin'=14) ('cycloserin 250mg'=3) ('Cycloserin 250mg'=3) ('Cycloserin 250 mg'=3) ('cycloserin 0.25g'=3) ('cycloserin 250'=3)

('cycloserin 0,25g'=3) ('Cycloserin 0,25g'=3) ('cycloserin 0.25h'=3) ('Cycloserin'=3) ('cycloserin'=3) ('cycloserin 200mg'=3) ('bedaquiline'=15) ('rifampicin'=12) ('moxifloxacin'=8) ('moxifloxacin'=8) ('isoniazid'=5)

('Ethambutol 0,4 g'=4) (missing=sysmis) INTO thuoc5a.

execute.

Recode thuoc6 (CONVERT) ('amikacin'=1) ('capreomycin'=2) ('ethambutol'=4) ('ethambutol 0,4mg'=4) ('ethambutol 400mg'=4) ('ethambutol 400'=4) ('ethambutol 400MG'=4) ('ethambutol 0,4g'=4)

('Ethambutol 0,4g'=4) ('ethambutol 0.4g'=4) ('kanamycin'=6) ('Kanamycin'=6) ('kanamycin ( tiem cach ngay)'=6) ('kanamycin (tiem cach ngay)'=6) ('kanamycin 1g'=6) ('Kanamycin 1g'=6)

('kanamycin( tiem cach ngay)'=6) ('kanamycin(giam lieu)'=6) ('kanamycin1g'=6) ('levofloxacin'=7) ('levofloxacin 0,25g'=7) ('Levofloxacin 0,25g'=7) ('levofloxacin 0.25g'=7) ('Levofloxacin 250 mg'=7)

('levofloxacin 250mg'=7) ('Levofloxacin 250mg'=7) ('levofloxacin 250 mg'=7) ('levofloxacin 50mg'=7) ('prothionamid'=10) ('prothionamid 0.25g'=10) ('prothionamid 250 mg'=10) ('prothionamid 250mg'=10)

('Prothionamid 250mg'=10) ('Prothionamid 0,25g'=10) ('prothionamid 0,25g'=10) ('Prothionamid'=10) ('prothionamid250mg'=10) ('pyrazinamid'=11) ('Pyrazinamid 0,5g'=11) ('pyrazinamid 0,5g'=11)

('Pyrazinamid'=11) ('pyrazinamid 500 mg'=11) ('pyrazinamid 500mg'=11) ('pyrazinamid 0.5g'=11) ('pyrazinamid 0.5gg'=11) ('pyrazinamid 0,4g'=11) ('pyrazinamid ( giam lieu)'=11) ('pyrazinamid (giam lieu)'=11)

('pyrazinamid 0,5 g'=11) ('linezolid'=13) ('PAS 4g'=9) ('PAS'=9) ('PAP'=9) ('clofazimin'=14) ('cycloserin 250mg'=3) ('Cycloserin 250mg'=3) ('Cycloserin 250 mg'=3) ('cycloserin 0.25g'=3) ('cycloserin 250'=3)

('cycloserin 0,25g'=3) ('Cycloserin 0,25g'=3) ('cycloserin 0.25h'=3) ('Cycloserin'=3) ('cycloserin'=3) ('cycloserin 200mg'=3) ('bedaquiline'=15) ('rifampicin'=12) ('moxifloxacin'=8) ('moxifloxacin'=8) ('isoniazid'=5)

('Ethambutol 0,4 g'=4) (else=99) (missing=sysmis) INTO thuoc6a.

execute.

Recode thuoc7 (CONVERT) ('amikacin'=1) ('capreomycin'=2) ('ethambutol'=4) ('ethambutol 0,4mg'=4) ('ethambutol 400mg'=4) ('ethambutol 400'=4) ('ethambutol 400MG'=4) ('ethambutol 0,4g'=4)

('Ethambutol 0,4g'=4) ('ethambutol 0.4g'=4) ('kanamycin'=6) ('Kanamycin'=6) ('kanamycin ( tiem cach ngay)'=6) ('kanamycin (tiem cach ngay)'=6) ('kanamycin 1g'=6) ('Kanamycin 1g'=6)

('kanamycin( tiem cach ngay)'=6) ('kanamycin(giam lieu)'=6) ('kanamycin1g'=6) ('levofloxacin'=7) ('levofloxacin 0,25g'=7) ('Levofloxacin 0,25g'=7) ('levofloxacin 0.25g'=7) ('Levofloxacin 250 mg'=7)

('levofloxacin 250mg'=7) ('Levofloxacin 250mg'=7) ('levofloxacin 250 mg'=7) ('levofloxacin 50mg'=7) ('prothionamid'=10) ('prothionamid 0.25g'=10) ('prothionamid 250 mg'=10) ('prothionamid 250mg'=10)

('Prothionamid 250mg'=10) ('Prothionamid 0,25g'=10) ('prothionamid 0,25g'=10) ('Prothionamid'=10) ('prothionamid250mg'=10) ('pyrazinamid'=11) ('Pyrazinamid 0,5g'=11) ('pyrazinamid 0,5g'=11)

('Pyrazinamid'=11) ('pyrazinamid 500 mg'=11) ('pyrazinamid 500mg'=11) ('pyrazinamid 0.5g'=11) ('pyrazinamid 0.5gg'=11) ('pyrazinamid 0,4g'=11) ('pyrazinamid ( giam lieu)'=11) ('pyrazinamid (giam lieu)'=11)

('pyrazinamid 0,5 g'=11) ('linezolid'=13) ('PAS 4g'=9) ('PAS'=9) ('PAP'=9) ('clofazimin'=14) ('cycloserin 250mg'=3) ('Cycloserin 250mg'=3) ('Cycloserin 250 mg'=3) ('cycloserin 0.25g'=3) ('cycloserin 250'=3)

('cycloserin 0,25g'=3) ('Cycloserin 0,25g'=3) ('cycloserin 0.25h'=3) ('Cycloserin'=3) ('cycloserin'=3) ('cycloserin 200mg'=3) ('bedaquiline'=15) ('rifampicin'=12) ('moxifloxacin'=8) ('moxifloxacin'=8) ('isoniazid'=5)

('Ethambutol 0,4 g'=4) (else=99) (missing=sysmis) INTO thuoc7a.

execute.

Recode thuoc8 (CONVERT) ('amikacin'=1) ('capreomycin'=2) ('ethambutol'=4) ('ethambutol 0,4mg'=4) ('ethambutol 400mg'=4) ('ethambutol 400'=4) ('ethambutol 400MG'=4) ('ethambutol 0,4g'=4)

('Ethambutol 0,4g'=4) ('ethambutol 0.4g'=4) ('kanamycin'=6) ('Kanamycin'=6) ('kanamycin ( tiem cach ngay)'=6) ('kanamycin (tiem cach ngay)'=6) ('kanamycin 1g'=6) ('Kanamycin 1g'=6)

('kanamycin( tiem cach ngay)'=6) ('kanamycin(giam lieu)'=6) ('kanamycin1g'=6) ('levofloxacin'=7) ('levofloxacin 0,25g'=7) ('Levofloxacin 0,25g'=7) ('levofloxacin 0.25g'=7) ('Levofloxacin 250 mg'=7)

('levofloxacin 250mg'=7) ('Levofloxacin 250mg'=7) ('levofloxacin 250 mg'=7) ('levofloxacin 50mg'=7) ('prothionamid'=10) ('prothionamid 0.25g'=10) ('prothionamid 250 mg'=10) ('prothionamid 250mg'=10)

('Prothionamid 250mg'=10) ('Prothionamid 0,25g'=10) ('prothionamid 0,25g'=10) ('Prothionamid'=10) ('prothionamid250mg'=10) ('pyrazinamid'=11) ('Pyrazinamid 0,5g'=11) ('pyrazinamid 0,5g'=11)

('Pyrazinamid'=11) ('pyrazinamid 500 mg'=11) ('pyrazinamid 500mg'=11) ('pyrazinamid 0.5g'=11) ('pyrazinamid 0.5gg'=11) ('pyrazinamid 0,4g'=11) ('pyrazinamid ( giam lieu)'=11) ('pyrazinamid (giam lieu)'=11)

('pyrazinamid 0,5 g'=11) ('linezolid'=13) ('PAS 4g'=9) ('PAS'=9) ('PAP'=9) ('clofazimin'=14) ('cycloserin 250mg'=3) ('Cycloserin 250mg'=3) ('Cycloserin 250 mg'=3) ('cycloserin 0.25g'=3) ('cycloserin 250'=3)

('cycloserin 0,25g'=3) ('Cycloserin 0,25g'=3) ('cycloserin 0.25h'=3) ('Cycloserin'=3) ('cycloserin'=3) ('cycloserin 200mg'=3) ('bedaquiline'=15) ('rifampicin'=12) ('moxifloxacin'=8) ('moxifloxacin'=8) ('isoniazid'=5)

('Ethambutol 0,4 g'=4) (else=99) (missing=sysmis) INTO thuoc8a.

execute.

recode thuoc1a (MISSING=99).

Execute.

recode thuoc2a (MISSING=99).

Execute.

recode thuoc3a (MISSING=99).

Execute.

recode thuoc4a (MISSING=99).

Execute.

recode thuoc5a (MISSING=99).

Execute.

recode thuoc6a (MISSING=99).

Execute.

recode thuoc7a (MISSING=99).

Execute.

recode thuoc8a (MISSING=99).

Execute.

*** Chuyen ma thuoc va lieu dung

String lanz (a20).

String lieuz (A20).

DO IF thuoc1a=11.

COMPUTE z=1.

compute lieuz=lieuthuoc1.

compute lanz=lanthuoc1.

ELSE IF thuoc2a=11.

compute z=1.

Compute lieuz=lieuthuoc2.

compute lanz=lanthuoc2.

ELSE IF thuoc3a=11.

compute z=1.

Compute lieuz=lieuthuoc3.

compute lanz=lanthuoc3.

ELSE IF thuoc4a=11.

compute z=1.

Compute lieuz=lieuthuoc4.

compute lanz=lanthuoc4.

ELSE IF thuoc5a=11.

compute z=1.

Compute lieuz=lieuthuoc5.

compute lanz=lanthuoc5.

ELSE IF thuoc6a=11.

compute z=1.

Compute lieuz=lieuthuoc6.

compute lanz=lanthuoc6.

ELSE IF thuoc7a=11.

compute z=1.

Compute lieuz=lieuthuoc7.

compute lanz=lanthuoc7.

ELSE IF thuoc8a=11.

compute z=1.

Compute lieuz=lieuthuoc8.

compute lanz=lanthuoc8.

ELSE.

compute z=0.

compute lieuz="0".

END IF.

ALTER TYPE lanz(f10).

ALTER TYPE lieuz(f10).

recode lanz (3=1) (11=1).

Compute lieuza = lanz*lieuz.

Execute.

recode lieuza (missing=0).

String lanam (a20).

String lieuam (a20).

DO IF thuoc1a=1.

COMPUTE am=1.

compute lieuam=lieuthuoc1.

compute lanam=lanthuoc1.

ELSE IF thuoc2a=1.

compute am=1.

Compute lieuam=lieuthuoc2.

compute lanam=lanthuoc2.

ELSE IF thuoc3a=1.

compute am=1.

Compute lieuam=lieuthuoc3.

compute lanam=lanthuoc3.

ELSE IF thuoc4a=1.

compute am=1.

Compute lieuam=lieuthuoc4.

compute lanam=lanthuoc4.

ELSE IF thuoc5a=1.

compute am=1.

Compute lieuam=lieuthuoc5.

compute lanam=lanthuoc5.

ELSE IF thuoc6a=1.

compute am=1.

Compute lieuam=lieuthuoc6.

compute lanam=lanthuoc6.

ELSE IF thuoc7a=1.

compute am=1.

Compute lieuam=lieuthuoc7.

compute lanam=lanthuoc7.

ELSE IF thuoc8a=1.

compute am=1.

Compute lieuam=lieuthuoc8.

compute lanam=lanthuoc8.

ELSE.

compute am=0.

compute lieuam="0".

END IF.

ALTER TYPE lanam(f10).

ALTER TYPE lieuam(f10).

Compute lieuama = lanam*lieuam.

Execute.

recode lieuama (missing=0).

String lancs (a20).

String lieucs (a20).

DO IF thuoc1a=3.

COMPUTE cs=1.

compute lieucs=lieuthuoc1.

compute lancs=lanthuoc1.

ELSE IF thuoc2a=3.

compute cs=1.

Compute lieucs=lieuthuoc2.

compute lancs=lanthuoc2.

ELSE IF thuoc3a=3.

compute cs=1.

Compute lieucs=lieuthuoc3.

compute lancs=lanthuoc3.

ELSE IF thuoc4a=3.

compute cs=1.

Compute lieucs=lieuthuoc4.

compute lancs=lanthuoc4.

ELSE IF thuoc5a=3.

compute cs=1.

Compute lieucs=lieuthuoc5.

compute lancs=lanthuoc5.

ELSE IF thuoc6a=3.

compute cs=1.

Compute lieucs=lieuthuoc6.

compute lancs=lanthuoc6.

ELSE IF thuoc7a=3.

compute cs=1.

Compute lieucs=lieuthuoc7.

compute lancs=lanthuoc7.

ELSE IF thuoc8a=3.

compute cs=1.

Compute lieucs=lieuthuoc8.

compute lancs=lanthuoc8.

ELSE.

compute cs=0.

compute lieucs="0".

END IF.

ALTER TYPE lancs(f10).

ALTER TYPE lieucs(f10).

Compute lieucsa = lancs*lieucs.

Execute.

recode lieucsa (missing=0).

String lane (a20).

String lieue (a20).

DO IF thuoc1a=4.

COMPUTE e=1.

compute lieue=lieuthuoc1.

compute lane=lanthuoc1.

ELSE IF thuoc2a=4.

compute e=1.

Compute lieue=lieuthuoc2.

compute lane=lanthuoc2.

ELSE IF thuoc3a=4.

compute e=1.

Compute lieue=lieuthuoc3.

compute lane=lanthuoc3.

ELSE IF thuoc4a=4.

compute e=1.

Compute lieue=lieuthuoc4.

compute lane=lanthuoc4.

ELSE IF thuoc5a=4.

compute e=1.

Compute lieue=lieuthuoc5.

compute lane=lanthuoc5.

ELSE IF thuoc6a=4.

compute e=1.

Compute lieue=lieuthuoc6.

compute lane=lanthuoc6.

ELSE IF thuoc7a=4.

compute e=1.

Compute lieue=lieuthuoc7.

compute lane=lanthuoc7.

ELSE IF thuoc8a=4.

compute e=1.

Compute lieue=lieuthuoc8.

compute lane=lanthuoc8.

ELSE.

compute e=0.

compute lieue="0".

END IF.

ALTER TYPE lane(f10).

ALTER TYPE lieue(f10).

recode lane (3=1).

Compute lieuetha = lane*lieue.

Execute.

recode lieuetha (missing=0).

DELETE VARIABLES lankm lieukm.

String lankm (a20).

String lieukm (a20).

DO IF thuoc1a=6.

COMPUTE km=1.

compute lieukm=lieuthuoc1.

compute lankm=lanthuoc1.

ELSE IF thuoc2a=6.

compute km=1.

Compute lieukm=lieuthuoc2.

compute lankm=lanthuoc2.

ELSE IF thuoc3a=6.

compute km=1.

Compute lieukm=lieuthuoc3.

compute lankm=lanthuoc3.

ELSE IF thuoc4a=6.

compute km=1.

Compute lieukm=lieuthuoc4.

compute lankm=lanthuoc4.

ELSE IF thuoc5a=6.

compute km=1.

Compute lieukm=lieuthuoc5.

compute lankm=lanthuoc5.

ELSE IF thuoc6a=6.

compute km=1.

Compute lieukm=lieuthuoc6.

compute lankm=lanthuoc6.

ELSE IF thuoc7a=6.

compute km=1.

Compute lieukm=lieuthuoc7.

compute lankm=lanthuoc7.

ELSE IF thuoc8a=6.

compute km=1.

Compute lieukm=lieuthuoc8.

compute lankm=lanthuoc8.

ELSE.

compute km=0.

compute lieukm="0".

END IF.

ALTER TYPE lankm(f10).

ALTER TYPE lieukm(f10).

Compute lieukma = lankm*lieukm.

Execute.

recode lieukma (missing=0).

DELETE VARIABLES lancm lieucm.

String lancm (a20).

String lieucm (a20).

DO IF thuoc1a=2.

COMPUTE cm=1.

compute lieucm=lieuthuoc1.

compute lancm=lanthuoc1.

ELSE IF thuoc2a=2.

compute cm=1.

Compute lieucm=lieuthuoc2.

compute lancm=lanthuoc2.

ELSE IF thuoc3a=2.

compute cm=1.

Compute lieucm=lieuthuoc3.

compute lancm=lanthuoc3.

ELSE IF thuoc4a=2.

compute cm=1.

Compute lieucm=lieuthuoc4.

compute lancm=lanthuoc4.

ELSE IF thuoc5a=2.

compute cm=1.

Compute lieucm=lieuthuoc5.

compute lancm=lanthuoc5.

ELSE IF thuoc6a=2.

compute cm=1.

Compute lieucm=lieuthuoc6.

compute lancm=lanthuoc6.

ELSE IF thuoc7a=2.

compute cm=1.

Compute lieucm=lieuthuoc7.

compute lancm=lanthuoc7.

ELSE IF thuoc8a=2.

compute cm=1.

Compute lieucm=lieuthuoc8.

compute lancm=lanthuoc8.

ELSE.

compute cm=0.

compute lieucm="0".

END IF.

ALTER TYPE lancm(f10).

ALTER TYPE lieucm(f10).

Compute lieucma = lancm*lieucm.

Execute.

recode lieucm (missing=0).

DO IF thuoc1a=7.

COMPUTE lfx=1.

compute lieulfx=lieuthuoc1.

compute lanlfx=lanthuoc1.

ELSE IF thuoc2a=7.

compute lfx=1.

Compute lieulfx=lieuthuoc2.

compute lanlfx=lanthuoc2.

ELSE IF thuoc3a=7.

compute lfx=1.

Compute lieulfx=lieuthuoc3.

compute lanlfx=lanthuoc3.

ELSE IF thuoc4a=7.

compute lfx=1.

Compute lieulfx=lieuthuoc4.

compute lanlfx=lanthuoc4.

ELSE IF thuoc5a=7.

compute lfx=1.

Compute lieulfx=lieuthuoc5.

compute lanlfx=lanthuoc5.

ELSE IF thuoc6a=7.

compute lfx=1.

Compute lieulfx=lieuthuoc6.

compute lanlfx=lanthuoc6.

ELSE IF thuoc7a=7.

compute lfx=1.

Compute lieulfx=lieuthuoc7.

compute lanlfx=lanthuoc7.

ELSE IF thuoc8a=7.

compute lfx=1.

Compute lieulfx=lieuthuoc8.

compute lanlfx=lanthuoc8.

ELSE.

compute lfx=0.

compute lieulfx=0.

END IF.

Compute lieulfxa = lanlfx*lieulfx.

Execute.

recode lieulfxa (missing=0).

DELETE VARIABLES lanmxf lieumxf.

String lanmxf (a20).

String lieumxf (A20).

DO IF thuoc1a=8.

COMPUTE mxf=1.

compute lieumxf=lieuthuoc1.

compute lanmxf=lanthuoc1.

ELSE IF thuoc2a=8.

compute mxf=1.

Compute lieumxf=lieuthuoc2.

compute lanmxf=lanthuoc2.

ELSE IF thuoc3a=8.

compute mxf=1.

Compute lieumxf=lieuthuoc3.

compute lanmxf=lanthuoc3.

ELSE IF thuoc4a=8.

compute mxf=1.

Compute lieumxf=lieuthuoc4.

compute lanmxf=lanthuoc4.

ELSE IF thuoc5a=8.

compute mxf=1.

Compute lieumxf=lieuthuoc5.

compute lanmxf=lanthuoc5.

ELSE IF thuoc6a=8.

compute mxf=1.

Compute lieumxf=lieuthuoc6.

compute lanmxf=lanthuoc6.

ELSE IF thuoc7a=8.

compute mxf=1.

Compute lieumxf=lieuthuoc7.

compute lanmxf=lanthuoc7.

ELSE IF thuoc8a=8.

compute mxf=1.

Compute lieumxf=lieuthuoc8.

compute lanmxf=lanthuoc8.

ELSE.

compute mxf=0.

compute lieumxf="0".

END IF.

ALTER TYPE lanmxf(f10).

ALTER TYPE lieumxf(f10).

Compute lieumxfa = lanmxf*lieumxf.

Execute.

recode lieumxfa (missing=0).

DELETE VARIABLES lieulfxn lanlfxn.

DELETE VARIABLES lanpas lieupas.

String lanpas (a20).

String lieupas (A20).

DO IF thuoc1a=9.

COMPUTE pas=1.

compute lieupas=lieuthuoc1.

compute lanpas=lanthuoc1.

ELSE IF thuoc2a=9.

compute pas=1.

Compute lieupas=lieuthuoc2.

compute lanpas=lanthuoc2.

ELSE IF thuoc3a=9.

compute pas=1.

Compute lieupas=lieuthuoc3.

compute lanpas=lanthuoc3.

ELSE IF thuoc4a=9.

compute pas=1.

Compute lieupas=lieuthuoc4.

compute lanpas=lanthuoc4.

ELSE IF thuoc5a=9.

compute pas=1.

Compute lieupas=lieuthuoc5.

compute lanpas=lanthuoc5.

ELSE IF thuoc6a=9.

compute pas=1.

Compute lieupas=lieuthuoc6.

compute lanpas=lanthuoc6.

ELSE IF thuoc7a=9.

compute pas=1.

Compute lieupas=lieuthuoc7.

compute lanpas=lanthuoc7.

ELSE IF thuoc8a=9.

compute pas=1.

Compute lieupas=lieuthuoc8.

compute lanpas=lanthuoc8.

ELSE.

compute pas=0.

compute lieupas="0".

END IF.

ALTER TYPE lanpas(f10).

ALTER TYPE lieupas(f10).

Compute lieupasa = lanpas*lieupas.

Execute.

recode lieupasa (missing=0).

DELETE VARIABLES lanpto lieupto.

String lanpto (a20).

String lieupto (A20).

DO IF thuoc1a=10.

COMPUTE pto=1.

compute lieupto=lieuthuoc1.

compute lanpto=lanthuoc1.

ELSE IF thuoc2a=10.

compute pto=1.

Compute lieupto=lieuthuoc2.

compute lanpto=lanthuoc2.

ELSE IF thuoc3a=10.

compute pto=1.

Compute lieupto=lieuthuoc3.

compute lanpto=lanthuoc3.

ELSE IF thuoc4a=10.

compute pto=1.

Compute lieupto=lieuthuoc4.

compute lanpto=lanthuoc4.

ELSE IF thuoc5a=10.

compute pto=1.

Compute lieupto=lieuthuoc5.

compute lanpto=lanthuoc5.

ELSE IF thuoc6a=10.

compute pto=1.

Compute lieupto=lieuthuoc6.

compute lanpto=lanthuoc6.

ELSE IF thuoc7a=10.

compute pto=1.

Compute lieupto=lieuthuoc7.

compute lanpto=lanthuoc7.

ELSE IF thuoc8a=10.

compute pto=1.

Compute lieupto=lieuthuoc8.

compute lanpto=lanthuoc8.

ELSE.

compute pto=0.

compute lieupto="0".

END IF.

ALTER TYPE lanpto(f10).

ALTER TYPE lieupto(f10).

Compute lieuptoa = lanpto*lieupto.

Execute.

recode lieuptoa (missing=0).

****DO THANH THAI CREATININ BAN DAU****

if gioia=0 crcr=(140-tuoi)*nangbd/(72*cr0)*0.85/0.011312217194570135.

if gioia=1 crcr=(140-tuoi)*nangbd/(72*cr0)/0.011312217194570135.

CREATE Table1x.IDlead=LEAD(Table1x.ID,1).

recode Table1x.IDlead (missing=0).

Do if (Table1x.ID = Table1x.IDlead) AND (BNchuyendikp=1).

compute check=1000000.

end if.

*** Bien doi bien co****

RECODE bc1 (CONVERT) ('0=khong co'=0) ('1=non'=1) ('10=rl tam than'=10) ('11=tk ngoai vi'=11) ('12=dau khop'=12) ('12=nhuc moi tay'=12) ('13=qua man'=13) ('14=doc than'=14)

('14=doc than nghi ngo'=14) ('142=doc than nghi ngo'=14) ('142=nghi ngo doc than'=14) ('15=tien dinh'=15) ('16=thi giac'=16) ('17=suy giap'=17) ('18=ha K mau'=18) ('19=tang uric'=19)

('2=tieu chay'=2) ('20=rl huyet hoc'=20) ('20=rl huyet hoc, giam tieu cau'=20) ('22=pu tai cho'=22) ('23=soc'=23) ('25=dien tien cua lao'=99) ('3=dau bung'=3) ('4=chan an'=4)

('51=da day xac dinh'=51) ('52=da day nghi ngo'=52) ('61=doc gan xac dinh'=61) ('62=doc gan nghi ngo'=62) ('7=dau dau'=7) ('7=đau dau'=7) ('8=hoa mat'=8) ('9=co giat'=9)

('99= benh ly duong ho hap'=99) ('99= dau dau vu'=99) ('99= ho khan'=99) ('99=benh ly duong ho hap'=99) ('99=benh nhan bi gay chan'=99) ('99=benh nhan khong tai kham'=99)

('99=benh nhan tre hen'=99) ('99=benh tim'=99) ('99=chay mau cam'=99) ('99=chuong bung'=99) ('99=chuyen vien dieu tri lao sieu khang'=99) ('99=co rut chan tay'=99)

('99=dai tien phan nhay mu'=99) ('99=dau co'=99) ('99=dau khap co the'=7) ('99=dau lung'=99) ('99=dau lung/dau nguoi'=99) ('99=dau nguoi, cac khop'=12) ('99=dau that lung'=99)

('99=đau tức ngực'=99) ('99=đau tức ngực nhẹ, khó thở nhẹ, ho nhiều'=99) ('99=đau tức ngực, khó thở vừa'=99) ('99=dien tien cua benh lao'=99) ('99=dien tien cua lao'=99)

('99=do dam'=99) ('99=ha duong huyet'=99) ('99=ha duong huyet va tang bach cau'=99) ('99=hat hoi chay mau'=99) ('99=hay quen'=99) ('99=ho'=99) ('99=ho it'=99) ('99=ho nhieu'=99)

('99=ho nhieu met ngu khong duoc'=99) ('99=ho ra mau'=99) ('99=ho ra máu'=99) ('99=ho, sot, an uong kem'=99) ('99=hong viem do'=99) ('99=kho mieng'=99) ('99=kho tho'=99)

('99=khong tai kham'=99) ('99=met'=27) ('99=met moi'=27) ('99=met, an uong kem, kho tieu'=27) ('99=met, kho tho'=27) ('99=met, nuot kho'=27) ('99=met, yeu'=27)

('99=met, yeu, di lai kho'=27) ('99=met,ho'=27) ('99=met,sam da'=27) ('99=met,say'=27) ('99=met,tieu it'=27) ('99=met/sut can'=99) ('99=mun mu ngoai da'=99)

('99=mun mu tay do'=99) ('99=mun nuoc vung dau mat co'=99) ('99=nhuc tay'=99) ('99=non ra mau'=99) ('99=nong bung sau uong thuoc'=99) ('99=phu ne ban tay phai'=99)

('99=roi loan nhip tim'=99) ('99=run'=99) ('99=rung toc'=99) ('99=sam da'=99) ('99=sam da, hay quen'=99) ('99=say,met'=27) ('99=so mui'=99) ('99=sot'=99) ('99=sot cao'=99)

('99=sot cao ve chieu'=99) ('99=sot nong/ho khan'=99) ('99=sot/ho'=99) ('99=sut can'=99) ('99=suy kiet'=99) ('99=tang bach cau'=99) ('99=tang duong huyet'=32)

('99=tang kali mau'=99) ('99=tao bon'=99) ('99=thay doi vi giac'=99) ('99=thieu dien giai'=99) ('99=tien trien cua benh lao'=99) ('99=tim bat thuong'=99) ('99=tran khi mang phoi'=99)

('99=tu vong'=99) ('99=tử vong'=99) ('99=tu y bo thuoc'=99) ('99=viem amidal nhieu'=99) ('99=viem tuy cap, xuat huyet'=99) ('99=xuat huyet da day - dai trang'=99) (ELSE=99) (MISSING=SYSMIS) INTO bc1a.

EXECUTE.

RECODE bc1a (MISSING=99).

EXECUTE.

Value labels bc1a

0 'khong co' 1 'non' 2 'tieu chay' 3 'dau bung' 4 'chan an' 51 'da day xac dinh' 52 'da day nghi ngo' 61 'doc gan xac dinh' 62 'doc gan nghi ngo' 7 'dau dau' 8 'hoa mat' 9 'co giat' 10 'rl tam than'

11 'tk ngoai vi' 12 'dau khop' 13 'qua man' 14 'doc than' 15 'tien dinh' 16 'thi giac' 17 'suy giap' 18 'ha K mau' 19 'tang uric' 20 'rl huyet hoc' 21 'rl noi tiet' 22 'pu tai cho' 23 'soc' 27 'met' 32 'tang duong huyet' 99 'missing'.

execute.

RECODE bc2 (CONVERT) ('0=khong co'=0) ('1=non'=1) ('10=rl tam than'=10) ('11=tk ngoai vi'=11) ('12=dau khop'=12) ('13=qua man'=13) ('14=doc than'=14) ('15=tien dinh'=15)

('16=thi giac'=16) ('17=suy giap'=17) ('18=ha K mau'=18) ('19=tang uric'=19) ('2=tieu chay'=2) ('20=rl huyet hoc'=20) ('21=rl noi tiet'=21) ('22=pu tai cho'=22) ('3=dau bung'=3)

('4=chan an'=4) ('51=da day xac dinh'=51) ('52=da day nghi ngo'=52) ('61=doc gan xac dinh'=61) ('62=doc gan nghi ngo'=62) ('7=đau dau'=7) ('8=hoa mat'=8) ('9=co giat'=9)

('99=benh ly duong ho hap'=99) ('99=dau co'=99) ('99=dau lung'=99) ('99=dien tien cua lao'=99) ('99=ha duong huyet'=32) ('99=ho dam'=99) ('99=ho khan'=99) ('99=ho nhieu'=99)

('99=ho nhieu, met, kho tho'=99) ('99=ho ra mau'=99) ('99=hoi hop, kho chiu, mat ngu'=99) ('99=khan giong'=99) ('99=kho tho'=99) ('99=met'=27) ('99=met,sut can (?suykiet)'=99)

('99=ngu kem'=99) ('99=ngua, do da vung khuyu tay, khop goi 2 ben'=13) ('99=nhuc moi nguoi'=99) ('99=nong tung con'=99) ('99=rung toc'=99) ('99=sam da'=99) ('99=sot'=99)

('99=sot cao'=99) ('99=sung chuoi hach co'=99) ('99=suy kiet'=99) ('99=tang bach cau'=99) ('99=tang CRP'=62) ('99=tang duong huyet'=32) ('99=tang PLT'=62)

('99=te bi tay chan'=99) ('99=thay doi mot so xet nghiem'=99) ('99=thieu dien giai'=99) (ELSE=99) (MISSING=SYSMIS) INTO bc2a.

EXECUTE.

RECODE bc2a (MISSING=99).

EXECUTE.

Value labels bc2a

0 'khong co' 1 'non' 2 'tieu chay' 3 'dau bung' 4 'chan an' 51 'da day xac dinh' 52 'da day nghi ngo' 61 'doc gan xac dinh' 62 'doc gan nghi ngo' 7 'dau dau' 8 'hoa mat' 9 'co giat' 10 'rl tam than'

11 'tk ngoai vi' 12 'dau khop' 13 'qua man' 14 'doc than' 15 'tien dinh' 16 'thi giac' 17 'suy giap' 18 'ha K mau' 19 'tang uric' 20 'rl huyet hoc' 21 'rl noi tiet' 22 'pu tai cho' 23 'soc' 27 'met' 32 'tang duong huyet' 99 'missing'.

execute.

RECODE bc3 (CONVERT) ('0=khong co'=0) ('1=non'=1) ('10=rl tam than'=10) ('11=tk ngoai vi'=11) ('12=dau khop'=12) ('13=qua man'=13) ('14=doc than'=14) ('142=doc than nghi ngo'=14)

('15=tien dinh'=15) ('16=thi giac'=16) ('17=suy giap'=17) ('18=ha K mau'=18) ('18=thieu dien giai-ha kali mau'=18) ('19=tang uric'=19) ('20=rl huyet hoc'=20) ('22=pu tai cho'=22)

('23=soc'=23) ('3=dau bung'=3) ('4=chan an'=4) ('52=da day nghi ngo'=52) ('61=doc gan xac dinh'=61) ('62=doc gan nghi ngo'=62) ('7=dau dau'=7) ('7=đau dau'=7) ('8=hoa mat'=8)

('9=co giat'=9) ('99=an uong kem'=99) ('99=dau nguoi'=99) ('99=dien tien cua lao'=99) ('99=ha duong huyet'=99) ('99=ho nhieu'=99) ('99=kho tho'=99) ('99=met'=27) ('99=met-roi loan tieu hoa'=27)

('99=mun nuoc noi rai rac'=99) ('99=ngu kem'=99) ('99=so thuoc'=99) ('99=sot'=99) ('99=tang bach cau'=99) ('99=tang duong huyet'=32) ('99=thay doi mot so xet nghiem'=99)

('99=thieu dien giai'=99) ('99=tu y bo thuoc'=99) ('99=xuat huyet vong mac mat'=99) (ELSE=99) (MISSING=SYSMIS) INTO bc3a.

EXECUTE.

RECODE bc3a (MISSING=99).

EXECUTE.

Value labels bc3a

0 'khong co' 1 'non' 2 'tieu chay' 3 'dau bung' 4 'chan an' 51 'da day xac dinh' 52 'da day nghi ngo' 61 'doc gan xac dinh' 62 'doc gan nghi ngo' 7 'dau dau' 8 'hoa mat' 9 'co giat' 10 'rl tam than'

11 'tk ngoai vi' 12 'dau khop' 13 'qua man' 14 'doc than' 15 'tien dinh' 16 'thi giac' 17 'suy giap' 18 'ha K mau' 19 'tang uric' 20 'rl huyet hoc' 21 'rl noi tiet' 22 'pu tai cho' 23 'soc' 27 'met' 32 'tang duong huyet' 99 'missing'.

execute.

RECODE bckhac (CONVERT) (' 62=doc gan nghi ngo'=62) ('1=buon non'=1) ('1=non'=1) ('10= rối loạn tâm thần'=10) ('10=rl tam than'=10) ('11=tk ngoai vi'=11) ('12=đau khớp'=12) ('13=qua man'=13)

('13=quá mẫn'=13) ('14=doc than'=14) ('14=doc than 14=doc than'=14) ('142=doc than nghi ngo'=14) ('15=rl tien dinh'=15) ('16=rl thi giac'=16) ('16=Rối loạn thị giác'=16)

('18= ha K mau'=18) ('18=ha K mau'=18) ('19=tang uric'=19) ('19=tang uric 19'=19) ('19=tang uric 19=tang uric'=19) ('20=rl huyet hoc'=20) ('20=rl huyet hoc 20=rl huyet hoc'=20)

('22=pu tai cho'=22) ('22=pu tai cho tiem 22=pu tai cho'=22) ('4=chan an'=4) ('52=da day nghi ngo'=52) ('61=doc gan xac dinh'=61) ('62=doc gan nghi ngo'=62) ('62=doc gan nghi ngo 62'=62)

('8=hoa mat'=8) ('8=hoa mat, chong mat 8'=8) ('99=bac toc, sam da 99='=99) ('99=benh ly duong ho hap'=99) ('99=dau dau-ho mat,chong mat'=7) ('99=dien tien cua lao'=99)

('99=ha duong huyet'=32) ('99=ho-sot'=99) ('99=met'=27) ('99=met 99='=27) ('99=met-an ngu kem'=27) ('99=met-cam thay nong nguoi'=27) ('99=met-dau dau'=27) ('99=met-ho'=27)

('99=met,ho nhieu'=27) ('99=nam phoi 99='=99) ('99=sot'=99) ('99=sot 99='=99) ('99=tang albumin'=99) ('99=tang bach cau'=99) ('99=tang duong huyet'=32) ('99=tang duong huyet glucose: 8.39'=32)

('99=thieu dien giai'=99) (ELSE=99) (MISSING=SYSMIS) INTO bckhaca.

EXECUTE.

recode bckhaca (MISSING=99).

EXECUTE.

Value labels bckhaca

0 'khong co' 1 'non' 2 'tieu chay' 3 'dau bung' 4 'chan an' 51 'da day xac dinh' 52 'da day nghi ngo' 61 'doc gan xac dinh' 62 'doc gan nghi ngo' 7 'dau dau' 8 'hoa mat' 9 'co giat' 10 'rl tam than'

11 'tk ngoai vi' 12 'dau khop' 13 'qua man' 14 'doc than' 15 'tien dinh' 16 'thi giac' 17 'suy giap' 18 'ha K mau' 19 'tang uric' 20 'rl huyet hoc' 21 'rl noi tiet' 22 'pu tai cho' 23 'soc' 27 'met' 32 'tang duong huyet' 99 'missing'.

execute.

**Bien doi muc do bien co***

recode mucdobc1 (convert) ('0=khong nghiem trong'=0) ('1=tu vong'=1) ('2=tinh mang'=2) ('3=am vien dai'=3) ('3=nam vien dai'=3) ('4=tan tat'=4) ('5=khac'=5) ('6=khong ro'=99)

('99=khác'=99) (else=99) into mucdobc1a.

execute.

value labels mucdobc1a

0 'khong nghiem trong' 1 'tu vong' 2 'tinh mang' 3 'nam vien dai' 4 'tan tat' 5 'nghiem trong khac' 99 'chua phan loai'.

execute.

recode mucdobc2 (convert) ('0=khong nghiem trong'=0) ('2=tinh mang'=2) ('3=nam vien dai'=3) ('4=tan tat'=4) ('5=khac'=5) ('5=nhap vien'=3) ('6=khong ro'=99) ('99=omeprazol'=99)

('99=uong nhieu nuoc'=99) (else=99) into mucdobc2a.

execute.

value labels mucdobc2a

0 'khong nghiem trong' 1 'tu vong' 2 'tinh mang' 3 'nam vien dai' 4 'tan tat' 5 'nghiem trong khac' 99 'chua phan loai'.

execute.

recode mucdobc3 (convert) ('0=khong nghiem trong'=0) ('2=tinh mang'=2) ('3=nam vien dai'=3) ('5=khac'=5) ('6=khong ro'=99) (else=99) into mucdobc3a.

execute.

value labels mucdobc3a

0 'khong nghiem trong' 1 'tu vong' 2 'tinh mang' 3 'nam vien dai' 4 'tan tat' 5 'nghiem trong khac' 99 'chua phan loai'.

execute.

recode mucdobckhac (convert) ('0=khong nghiem trong'=0) ('2=tinh mang'=2) ('3=nam vien dai'=3) ('5=khac'=5) ('6=khong ro'=99) (else=99) into mucdobckhaca.

execute.

value labels mucdobckhaca

0 'khong nghiem trong' 1 'tu vong' 2 'tinh mang' 3 'nam vien dai' 4 'tan tat' 5 'nghiem trong khac' 99 'chua phan loai'.

execute.

***Bien doi xu tri bien co****

recode xutribc1 (CONVERT) ('0=khong nghiem trong'=0) ('0=khong xu tri'=0) ('1=giam lieu'=1) ('1=giam lieu ( lieu kanamycin theo can nang)'=1) ('1=giam lieu kanamycin'=1) ('1=giam lieu kanamycin/99=tanakan'=1) ('1=giam lieu Km'=1)

('1=giam lieu LFX/99=omeprazol,attapulgit'=1) ('1=giam lieu PZA'=1) ('1=giam lieu PZA/99=allopurinol'=1) ('1=giam lieu PZA/99=meloxicam'=1) ('1=giam lieu PZA/99=uong nhieu nuoc'=1) ('1=giam lieu/99=VTM B6'=1)

('2=tam ngung'=2) ('2=tam ngung capreomycin'=2) ('2=tam ngung cycloserin 250mg'=2) ('2=tam ngung Emb'=2) ('2=tam ngung EMB'=2) ('2=tam ngung ethambutol, protionamid'=2) ('2=tam ngung kanamycin'=2) ('2=tam ngung kanamycin 1000mg'=2)

('2=tam ngung Km'=2) ('2=tam ngung PAS'=2) ('2=tam ngung Pto'=2) ('2=tam ngung Pto/99=thyroxin'=2) ('2=tam ngung pyrazinamid'=2) ('2=tam ngung PZA'=2) ('2=tam ngung PZA/44=truyen dich glucose 5%/99= L-ornithine-L-Aspartate'=2)

('2=tam ngung PZA/44=truyen dich glucose 5%/99=L-orthinin L-aspartate'=2) ('2=tam ngung PZA/99=uong nhieu nuoc'=2) ('2=tam ngung thuoc'=2) ('2=tam ngung toan bo thuoc'=2) ('3=doi thuoc'=3)

('4'=4) ('4=dungthuoc'=4) ('4=effelargan codein'=4) ('4=khang histamin'=4) ('41,42'=4) ('41; 42'=4) ('41=BAR,chlopheramin'=4) ('41=khang histamin'=4) ('41=khang histamin (phenergal)'=4) ('41=khang histamin (Promethazine)'=4)

('41=khang histamin + 42=corticoid'=4) ('41=khang histamin chlopheniramin'=4) ('41=khang histamin fexofenadine'=4) ('41=khang histamin fexofenadine chlorhydrate'=4) ('41=khang histamin fexofenadine/ 45 =thuoc boi da gentrisone'=4)

('41=khang histamin loratadin'=4) ('41=khang histamin,44'=4) ('41=khang histamin(cezil)'=4) ('41=khang histamin(elaria đặt hậu môn)'=4) ('41=khang histamin(elaua) đặt hậu môn'=4) ('41=khang histamin(meloxicam+vitamin B6)'=4)

('41=khang histamin/44=truyen dich ORS/99=VTM B1'=4) ('41=khang histamin/45=thuoc boi ngoai (betamethasone)'=4) ('41=khang histamin/99=silymarin'=4) ('41=khang histamin/paracetamol/riboflavin'=4) ('41=khang histamin+44=truyen dịch'=4)

('42=corticoid'=4) ('43=adrenalin'=4) ('44= glucose + 41=clopheniramin'=4) ('44= ringer lactat'=4) ('44=glucose + 99=l-ornithin l-aspartat'=4) ('44=glucose + 99=L-ornithin L-aspartat'=4)

('44=glucose 5%'=4) ('44=glucose 5% + 99= L-ornithin L-aspartat'=4)

('44=ringer lactat + 99=trofurid'=4) ('44=truyen dich'=4) ('44=truyen dich + 99=L-ornithin L-aspartat'=4) ('44=truyen dich Alvesin'=4)

('44=truyen dich gluciose 5%/99=L-ornithin L-aspartat'=4) ('44=truyen dich glucose 5%'=4)

('44=truyen dich glucose 5% /99= L-ornithine-L-Aspartate'=4) ('44=truyen dich glucose 5%/ 99=L-ornithine-L-Aspartate'=4)

('44=truyen dich glucose 5%/99= L-ornithine-L-Aspartate'=4) ('44=truyen dich glucose 5%/99= L-ornithine-L-Aspartate'=4)

('44=truyen dich glucose 5%/99=L-ornithin L-aspartat'=4) ('44=truyen dich glucose 5%/99=L-ornithin L-aspartate'=4)

('44=truyen dich glucose 5%/99=L-ornithine-L-Aspartate'=4) ('44=truyen dich glucose 55'=4) ('44=truyen dich KCl'=4) ('44=truyen dich lactat ringer'=4) ('44=truyen dich NaCl 0.9%,lactat ringer/99=potassium chlorid'=4)

('44=truyen dich NaCl 0.9%,ringerlactat'=4) ('44=truyen dich NaCl 0.9%/ glucose 5%'=4) ('44=truyen dich NaCl/99=clarythromycin/alpha chymotrypsin/VTM 3B/botecgan'=4)

('44=truyen dich NaCl/heparin'=4) ('44=truyen dich ringer lactat'=4)

('44=truyen dich( glucose 5%)'=4) ('44=truyen dich( glucose 5%)/99=bidogozin'=4) ('44=truyen dich( NaCl 0.9%)/99=tang cuong dinh duong'=4)

('44=truyen dich( NaCl)'=4) ('44=truyen dich( Nacl)/99=silymarin'=4) ('44=truyen dich(alversin)'=4)

('44=truyen dich(glucose 5%)/99=bidogozin'=4) ('44=truyen dich(morihepamin, glucose 5%)/99=bidogozin'=4)

('44=truyen dich(NaCl 0.9%)'=4) ('44=truyen dich(nephrosteril)/99=erythropoietin'=4) ('44=truyen dich/41=khang histamin'=4) ('44=truyen dich/99=biphenyl dimethyl dicarboxylate'=4) ('44=truyen dich/99=L-ornithine-L-Aspartate'=4)

('44=truyen dich/99=omeprazol, attapulgit'=4) ('44=truyen dich/99=toxacin'=4) ('44=truyen dich/99=uong nhieu nuoc'=4) ('44=truyen dichglucose 5%/99= L-ornithin - L-aspartat'=4) ('44=truyen dichglucose 5%/99=silymarin, Lornithin-Laspartat'=4)

('44=truyen dichNaCl 9%/99=etamsylate,acid transamin, terpin codein'=4) ('44=truyen dichNaCl 9%/99=L-ornithine-L-aspartate'=4) ('44=truyenhan nghii dichNaCl 9%/99=L-ornithine-L-aspartate'=4) ('45=thuoc boi ngoai gentrisone'=4) ('5=chay than'=5)

('6=chuyen vien'=6) ('6=chuyen vien/1=giam lieu PZA 50%'=1) ('6=chuyen vien/99=allopurinol'=6) ('6=chuyen vien/99=insulin'=6) ('6=chuyen vien/99=tanakan'=6) ('6=chuyen vien/99=VTM B6'=6) ('6=hướng dẫn nhập viện'=6) ('6=khám chuyên khoa mắt'=6)

('6=nhap vien PNT'=6) ('6=nhap vien PNT 5 ngay'=6) ('6=nhập viện theo dõi'=6) ('6=yêu cầu bệnh nhân nhập viện'=6) ('7=khong ro'=99) ('99'=99) ('99-diclofenac, omeprazol'=4) ('99='=99) ('99= Acetaminophen+Methionin+Magne B6'=4)

('99= acid tranexamique'=4) ('99= allopurinol'=4) ('99= an nhieu bua/ biphenyl dimethyl dicarboxylate'=4) ('99= aspirin'=4) ('99= B6'=4) ('99= Biphenyl dimethyl dicarbonxylat + 3B'=4) ('99= bromhexine hydrochloride'=4) ('99= Calci+Magne B6+Vit 3B'=4)

('99= calcium corbiere'=4) ('99= calcium-DL-3-methyl-2-oxo-valerate'=4) ('99= calcium-DL-3-methyl-2-oxo-valerate;uong nhieu nuoc'=4) ('99= celecoxib'=4) ('99= celecoxib + Acetaminophen'=4) ('99= Celecoxib + Acetaminophen'=4)

('99= Celecoxib + Acetaminophen+methionin'=4) ('99= celecoxib + Vit 3B'=4) ('99= celecoxib, mexcold'=4) ('99= celecoxib, paracetamol'=4) ('99= Celecoxib+Acetaminophen'=4) ('99= cinnarizine'=4) ('99= colchicin'=4) ('99= dang dieu tri tieu duong'=4)

('99= diclofenac sodium'=4) ('99= đo ECG,magie B6'=4) ('99= fexofenadine'=4) ('99= hoat huyet duong nao'=4) ('99= Ibuprofen+Acetaminnophen+Magne B6+Omeprazole'=4) ('99= ibuprofen+Acetaminnophen+Vit 3B'=4) ('99= Ibuprofen+Acetaminnophen+Vit 3B'=4)

('99= levothyroxin natri'=4) ('99= liverbil'=4) ('99= Liverbil (Actiso, Bìm Bìm, Biển Súc, Diệp Hạ Châu)'=4) ('99= Liverbil (Actiso, Bìm Bìm, Biển Súc, Diệp Hạ Châu)+Celecoxib + Acetaminophen'=4)

('99= Liverbil (Actiso, Bìm Bìm, Biển Súc, Diệp Hạ Châu)+Omeprazole'=4)

('99= livercom'=4) ('99= Magie B6'=4) ('99= Magie B6, omeprazol'=4) ('99= Magne B6'=4) ('99= magne B6, omeprazol, probio'=4) ('99= Magne B6+Omeprazole+Methionin'=4) ('99= meloxicam'=4)

('99= meloxicam; uong nhieu nuoc'=4) ('99= methionin, probio'=4) ('99= methionin, toprasin, probio'=4) ('99= mimosa'=4) ('99= Mimosa'=4) ('99= Mimosa (dược liệu) + Methionin'=4)

('99= Mimosa+Methionin+Toplexil'=4) ('99= Minosa (dược liệu) + Magie B6'=4) ('99= omepazol'=4) ('99= omeprazol'=4) ('99= omeprazol + Methionin'=4) ('99= omeprazol, megie B6'=4) ('99= omeprazol, methionin'=4) ('99= omeprazol, probio'=4)

('99= omeprazol, ranitidin'=4) ('99= Omeprazol; Magie B6'=4) ('99= omeprazole'=4) ('99= omeprazole+Acetaminophen'=4) ('99= oxomemazin hydroclorid, guaifenesin, Paracetamol, natri benzoat+calci+Magne B6'=4)

('99= oxomemazin hydroclorid, guaifenesin, Paracetamol, natri benzoat+Magne B6+Omeprazole+Liverbil (Actiso, Bìm Bìm, Biển Súc, Diệp Hạ Châu)'=4) ('99= paracetamol,ibuprofen'=4) ('99= potassium chlorid'=4) ('99= silymarin,diclofenac, VTM 3B'=4)

('99= tang cuong dinh duong'=4) ('99= theo doi'=4) ('99= trapadol, magie B6'=4) ('99= trapadol, magne B6'=4) ('99= trapadol, vit 3B, methionin'=4) ('99= tuan thu dieu tri'=4) ('99= VTM 3B'=4) ('99= xet nghiem gan, than'=4) ('99==VTM 3B'=4)

('99=acetyl cystein'=4) ('99=acetyl leucin, homtamin'=4) ('99=acid acetylsalicylic'=4) ('99=acid tranexamic'=4) ('99=acid tranexamic, trimetazidin'=4) ('99=Al(OH)3/Mg(OH)2/simethicon 30%'=4)

('99=allopurinlo'=4) ('99=allopurinol'=4) ('99=allopurinol, diclofenac'=4)

('99=Almitrin, Raubasin'=4) ('99=Almitrine+Raubasine'=4) ('99=alpha chymotrypsin;lansoprazol'=4) ('99=alphadeka'=4) ('99=alphadeka/meloxicam'=4) ('99=aluminum hydroxid,magie hydroxid'=4) ('99=aluminum hydroxide, magnesium hydroxide, simethicone'=4)

('99=aluminum hydroxide, magnesium hydroxide, Simethicone'=4) ('99=aluminum hydroxide/lasoprazol'=4) ('99=aluminumhydroxid/magie hydroxid/simethicon/ omeprazol'=4) ('99=amoxicilin; metronidazol;attapulgit'=4)

('99=an nhieu hoa qua, uong nhieu nuoc, VTM B6'=4) ('99=arginin'=4) ('99=aspirin'=4) ('99=atropin,attapulgite; omeprazol'=4) ('99=attapulgit'=4) ('99=attapulgit, omeprazol'=4)

('99=attapulgit/ omeprazol'=4) ('99=attapulgite, omeprazol'=4) ('99=autusin 1v 2 lan/ngay'=4) ('99=azithromycin'=4) ('99=azithromycin/nghi ngoi'=4) ('99=B6'=4) ('99=bcomlex'=4)

('99=biphenyl di methyl dicarboxylate'=4) ('99=biphenyl dimethl dicarboxylate'=4) ('99=biphenyl dimethyl dicarborxylate'=4) ('99=biphenyl dimethyl dicarboxylate'=4) ('99=bo bot'=4)

('99=bo gan'=4) ('99=boganic'=4) ('99=boganic; tang cuong dinh duong'=4) ('99=bromhexine hydrochloride'=4) ('99=cai thien che do an; VTM 3B'=4) ('99=calci D'=4)

('99=calcium'=4) ('99=Calcium-DL-3-methyl-2-oxo-valerat'=4) ('99=calcium-DL-3-methyl-2-oxo-valerate'=4) ('99=calcium-DL-3-methyl-2-oxo-valerate; erythropoietin'=4)

('99=cấp cứu ngừng tuần hoàn hô hấp'=6) ('99=cefmetazol'=4) ('99=ceftazidime, paracetamol'=4) ('99=celecoxib + paracetamol'=4) ('99=cerebrolysin/ diazepam/ amlodipin'=4)

('99=cetirizin'=4) ('99=cezil'=4) ('99=chlopheniramin'=4) ('99=cho BN nằm đầu cao, giảm ho (Terpin codein), 44: NaCl 0,9%/500ml'=4) ('99=chophyltol'=4) ('99=Cimetidin + Gastropulgit'=4) ('99=cinnarizin'=4)

('99=cinnarizine'=4) ('99=colchicin'=4) ('99=Colchicin'=4) ('99=colchicin + allopurinol'=4) ('99=cot thong linh'=4) ('99=cot thong linh/ meloxicam'=4) ('99=dau ca'=4) ('99=decontractyl'=4) ('99=Đên trạm y tế khám, dùng thuốc không rõ loại.'=6)

('99=dextromethorphan HBr'=4) ('99=diacerein,ketorolac tromethamin,paracetamol,ketoprofen'=4) ('99=diazepam'=4) ('99=diclofenac'=4) ('99=diclofenac, mephenesin'=4) ('99=diclofenac, omeprazol'=4) ('99=diclofenac, uong nhieu nuoc,omeprazol'=4)

('99=diclofenac; omeprazol'=4) ('99=diclofenac/ omeprazol'=4) ('99=diclophenac'=4) ('99=dieu tri trieu chung/99=metochlopramide'=4) ('99=digoxin'=4) ('99=Digoxin'=4) ('99=dizacetam+vitamin B6'=4) ('99=domperidom'=4) ('99=duxil'=4) ('99=Duxil'=4)

('99=efferangan'=4) ('99=erythropoietin'=4) ('99=erythropoietin; calcium-DL-3-methyl-2-oxo-valerate'=4) ('99=erythropoietin/ calcium-DL-3-methyl-2-oxo-valerate;'=4) ('99=erythropoietin/ calcium-DL-3-methyl-2-oxo-valerate;kham chuyen khoa'=6)

('99=etoricoxib'=4) ('99=fenoterol; ipratropium'=4) ('99=floctaphenine'=4) ('99=furosemid/kali oxid'=4) ('99=furosemid/kali oxid/calcium-DL-3-methyl-2-oxo-valerate'=4) ('99=gastophulgit'=4) ('99=gastropulgit'=4)

('99=gastropulgit + lactobacillus acidophilus'=4) ('99=giảm đau, theo dõi'=4) ('99=Gingo biloba'=4) ('99=ginkobiloba'=4) ('99=Ginkobiloba'=4) ('99=glucose'=4) ('99=glucose 5%; L-ornithine-L-Aspartate,ceftriaxon'=4) ('99=gumas'=4)

('99=gumas+lanzoprazol'=4) ('99=hepgaron'=4) ('99=hoat huyet duong nao'=4)

('99=hoi chan'=4) ('99=hồi sức tim phổi'=6) ('99=ibuprofen,mephenesine'=4) ('99=ibuprofen/vat ly tri lieu'=4) ('99=insulin'=4) ('99=kali clorid'=4) ('99=kali oxid'=4) ('99=kaliclorua'=4) ('99=kalioxid, furosemid'=4)

('99=kalioxid; furosemid'=4) ('99=kanamycin 1/2 lo, uong nhieu nuoc'=1) ('99=KCl'=4) ('99=kê đơn bổ gan (Fotex 2v/ngày)'=4) ('99=kê đơn bổ gan Fortex 2v/ngày'=4) ('99=kê đơn bổ gan. Forrtex 2v/ngày'=4) ('99=kê đơn Fortex 2v/ngày'=4)

('99=ketosteril'=4) ('99=khop tam binh'=4) ('99=L-ornithin L-aspartat, glucose 5%'=4) ('99=L-Ornithine-L-Aspartate/ NaCl 0,9%, dung thuoc lao hang II'=4)

('99=lactobacillus acidophilus'=4) ('99=lam xet nghiem ion do'=6) ('99=lan nuoc nong noi tiem'=4)

('99=lansoprazol'=4) ('99=lansoprazol + gumas'=4) ('99=lansopronol'=4) ('99=lanzopramid'=4) ('99=lanzoprazol'=4) ('99=lanzoprazol /vitamin B6'=4) ('99=lanzoprazol, alpha chymotrypsin'=4) ('99=lanzoprazol+doperidom'=4) ('99=lay mau xet nghiem'=6)

('99=liverbil'=4) ('99=livercom'=4) ('99=loperamid'=4) ('99=loperamid + oresol'=4) ('99=loratadin'=4) ('99=magie B6'=4) ('99=magne b6'=4) ('99=meloxicam'=4) ('99=meloxicam, omeprazol 20mg'=4) ('99=meloxicam, omeprazol'=4)

('99=meloxicam,omeprazol'=4) ('99=meloxicam; omeprazol'=4) ('99=meloxicam; uong nhieu nuoc'=4) ('99=meloxicam/ omeprazol'=4) ('99=meloxicam/ uong nhieu nuoc'=4) ('99=meloxicam/uong nhieu nuoc'=4)

('99=melxicam,colchicin'=4) ('99=men tieu hoa'=4) ('99=mephenesin, diclofenac'=4) ('99=mephenesin, diclofenac, 3B'=4) ('99=metformin'=4) ('99=metoclopramid'=4) ('99=Metoclopramid'=4) ('99=metoclopramid/ omeprazol'=4)

('99=metoclopramid/lanzoprazol'=4) ('99=metoclopramid/omeprazol'=4) ('99=metoclopramid+lanzoprazol'=4) ('99=metoclopramide'=4) ('99=mimosa'=4) ('99=NaCl 0.9%, xuc mieng buoi sang, trua, toi'=4) ('99=NaCl 0.9%/L-ornithine-L-Aspartate'=4)

('99=NaCl 9%/ acid tranesamic'=4) ('99=natri diclofenac'=4) ('99=natri diclofenac,allopurinol'=4) ('99=natri diclofenac,VTM 3B'=4) ('99=nephosteril'=4) ('99=nghi ngoi/ silymarin'=4) ('99=nghi ngoi/ tang cuong dinh duong/VTM B6/silymarin'=4)

('99=olanzapin'=4) ('99=olanzapin 5mg'=4) ('99=omeprazol'=4) ('99=omeprazol + gastropulgit'=4) ('99=omeprazol 20mg'=4) ('99=omeprazol, attapulgit'=4) ('99=omeprazol, attapulgite'=4) ('99=omeprazol, cetirizin'=4) ('99=omeprazol, cimetidin'=4)

('99=omeprazol, kremil S'=4) ('99=omeprazol, meloxicam'=4) ('99=omeprazol,aluminum hydroxid,magie hydroxid'=4) ('99=omeprazol,cinnarizine'=4) ('99=omeprazol/ attapulgit'=4) ('99=omeprazol/ attapulgite'=4) ('99=omeprazol/ gastropulgite'=4)

('99=omeprazol/ meloxicam'=4) ('99=omeprazol/attapulgit'=4) ('99=omeprazol/gastropulgit'=4) ('99=omeprazole'=4) ('99=paracetamol'=4) ('99=paracetamol,inbuprofen'=4) ('99=piracetam'=4) ('99=piracetam + duxil'=4) ('99=piracetam + vitamin B6'=4)

('99=piroxicam'=4) ('99=potassium chlorid'=4) ('99=Potassium chlorid'=4) ('99=prednisolon'=4) ('99=primeran'=4) ('99=primperam'=4) ('99=primperan'=4) ('99=promethazine'=4)

('99=protanol'=4) ('99=pyridroxin'=4) ('99=rotudin'=4) ('99=rotunda'=4) ('99=rotundin'=4) ('99=rotundine'=4) ('99=rutanda,VTM 3B'=4) ('99=seduxen'=4) ('99=silymarin'=4)

('99=Silymarin'=4) ('99=silymarin/ nghi ngoi tang cuong dinh duong'=4)

('99=silymarin/ VTM B6'=4) ('99=silymarin/ VTM B6/ tang cuong dinh duong'=4) ('99=silymarin/attapulgite/ L- ornithine L- Aspartate'=4) ('99=sucon-RB'=4) ('99=Symbicort'=4)

('99=tam sen'=4) ('99=tanakan'=4) ('99=tanakan, kham chuyen khoa'=6) ('99=tanakan, nghi ngoi'=4) ('99=tanakan,uong nhieu nuoc'=4) ('99=terbutaline'=4) ('99=terpin codein'=4)

('99=terpin codein/transamine 0.5g (2v/ng)'=4) ('99=thuoc chua da day'=4) ('99=thuoc giam dau'=4) ('99=toa thuoc'=4) ('99=tranexamic + 44=ringelactat, glucose'=4)

('99=tranexamique'=4) ('99=trang vi khang'=4) ('99=transamin'=4) ('99=trivitamin B(125,125,125)'=4) ('99=truyen khoi tieu cau'=6) ('99=tugeron'=4) ('99=uong giam dau'=4)

('99=uong nhieu nuoc, Calcium-DL-3-methyl-2-oxo-valerate'=4) ('99=uong nhieu nuoc;erythropoietin; calcium-DL-3-methyl-2-oxo-valerate/6=chuyen vien'=6)

('99=uong nhieu nuoc/ Calcium-DL-3-methyl-2-oxo-valerate'=4) ('99=uong nhieu nuoc/Calcium-DL-3-methyl-2-oxo-valerat'=4) ('99=uong sua/arginin'=4) ('99=varogel'=4)

('99=vat ly tri lieu'=4) ('99=vat ly tri lieu khop'=4) ('99=vitamin'=4) ('99=vitamin 3B'=4) ('99=Vitamin 3B'=4) ('99=vitamin A'=4) ('99=vitamin B1'=4) ('99=vitamin b6'=4)

('99=vitamin B6'=4) ('99=VTM 3B'=4) ('99=VTM B6'=4) ('99=VTM B6, tanakan'=4) ('99=VTM B6, uong nhieu nuoc'=4) ('99=VTM B6; tanakan'=4) ('99=VTM B6/i nghi ngoi'=4)

('99=VTM B6/tanakan/hoat huyet duong nao'=4) ('99meloxicam'=4) ('Al(OH)3 , Mg(OH)2,simethicon 30%'=4) ('Al(OH)3 , Mg(OH)2,simethicon 30%+metoclopramid+'=4)

('Al(OH)3/Mg(OH)2/simethicon 30%'=4) ('biphenyl dimethyl dicarboxylate'=4) ('biphenyl dimetyl dicarboxylate'=4) ('cezmeta'=4) ('Colchicin'=4) ('Diazepam'=4) ('elaua đặt hậu môn'=4)

('Gel Nhôm Hydroxyd; Magnesium hydroxide, Simethicone'=4) ('loratidin'=4) ('loreta'=4) ('Meloxicam 15mg, 2v/24h'=4) ('metoclopramid'=4) ('omeprazol'=4) ('omeprazol+ vitamin B6'=4)

('Omeprazole'=4) ('varogel+meyerazol+primperan'=4) ('varogel+omeprazol+pempreas'=4) ('Vitamin B1 0,01g,2ống; calciclorua 5ml'=4) ('dừng ethambutol0.4g cho bênhj nhân đi khám thị lực'=2)

('99= gia dinh van dong'=7) ('99=an bo sung'=7) ('99=an chuoi'=7) ('99=an chuoi tieu'=7) ('99=an chuoi tieu/cam'=7) ('99=an long, nhai ki'=7) ('99=an nhieu bua'=7)

('99=an nhieu bua/ uong nuoc hoa qua'=7) ('99=an nhieu lan'=7) ('99=an no truoc khi tiem'=7) ('99=benh nhan khong len lanh thuoc tu 08/12/2014 den nay'=7)

('99=cai thien che do an'=7) ('99=chuom'=7) ('99=chuom, thay doi vi tri'=7) ('99=chuom/ thay doi vi tri'=7) ('99=chụp x quang'=7) ('99=dieu chinh che do an'=7)

('99=do thinh luc'=6) ('99=HA:10/7cmhg'=7) ('99=khong dung qua lau/ ke chan goi khi nam'=7) ('99=không rõ'=7) ('99=kiem soat che do an'=7) ('99=kiem soat duong huyet'=7)

('99=kiem soat duong mau'=7) ('99=kiem tra huyet ap'=7) ('99=lien he voi gia dinh benh nhan'=7) ('99=nghi ngoi'=7) ('99=nghi ngoi, tang cuong dinh duong'=7)

('99=nghi ngoi, tang cuong suc khoe'=7) ('99=nghi ngoi,khong lao dong nang'=7) ('99=nghi ngoi/ tang cuong dinh dong'=7) ('99=nghi ngoi/ tang cuong dinh duong'=7)

('99=nghi ngoi/tang cuong dinh duong'=7) ('99=nhan tran'=7) ('99=tang cuong dinh duong'=7) ('99=tang cuong dinh duong, an nhieu hoa qua'=7) ('99=tang cuong dinh duong/nghi ngoi'=7) ('99=thay doi che do an'=7)

('99=thay the gio uong thuoc'=7) ('99=theo doi'=7) ('99=theo doi them'=7) ('99=theo dõi tiếp'=7) ('99=theo doi/xo giun'=7) ('99=tiếp tục theo dõi bệnh nhân'=7)

('99=tu van dinh duong'=7) ('99=tu van tam ly'=7) ('99=uoing nhieu nuoc'=7) ('99=uong nhieu nuoc'=7) ('99=uong nuoc tam sen'=7) ('99=uong nuoc, nghi ngoi'=7)

('99=uong tam sen'=7) ('991=nghi ngoi'=7) ('991=nghi ngoi, an uong nhieu hon'=7) ('991=theo doi'=7) ('991=uong nhieu nuoc'=7) ('99= chuyen vien nhung benh nhan khong nhap vien'=6)

('99=benh nhan khong dong y nhap vien'=6) ('99=BN được nhập viện để theo dõi'=6) ('99=chuyen vien nhung benh nhan khong nhap vien'=6) ('99=do dien nao do'=6)

('99=hương dẫn bn nhập viện theo dõi'=6) ('99=kham chuyen khoa'=6) ('99=kham noi tiet'=6) ('2=tam ngung/6=chuyen vien'=6) ('99=kham tam than'=6) ('99=nhap vien'=6) ('99=nhập viện'=6) ('99=soi day mat'=6)

('99=tang cuong dinh duong/ kham noi tiet'=6) ('99=tho oxy'=6) ('99=thở oxy 3l/ph 1h.'=6) ('99=doi thuoc'=3) ('99=khac'=99) ('99=tiếp tục theo dõi'=7) (else=99) into xutribc1a.

EXECUTE.

recode xutribc2 (CONVERT) ('0'=0) ('0=khong xu tri'=0) ('1=giam lieu'=1) ('1=giam lieu kanamycin'=1) ('1=giam lieu Km, PZA/99=silimarin'=1) ('2=tam ngung'=2)

('2=tam ngung Emb'=2) ('2=tam ngung kanamycin 1000mg'=2) ('2=tam ngung Lfx/Pto/Cs'=2) ('2=tam ngung PAS'=2) ('2=tam ngung PZA'=2) ('2=tam ngung thuoc lao hang II'=2)

('3=doi thuoc'=3) ('4=effelargan codein'=4) ('41; 42'=4) ('41=khang histamin'=4) ('41=khang histamin (Fexofenadine chlorhydrate)'=4) ('41=khang histamin (Lohatidin 10mg)'=4)

('41=khang histamin+44= truyen dịch'=4) ('44=ringer lactat'=4) ('44=ringer lactat + 99=trofurid'=4) ('44=truyen dich'=4) ('44=truyen dich + 99=bo gan'=4)

('44=truyen dich + 99=L-ornithin L-aspartat'=4) ('44=truyen dich gluciose 5%/99=L-ornithin L-aspartat'=4) ('44=truyen dich glucose 5%/99= L-ornithin L-aspartate'=4)

('44=truyen dich glucose 5%/99=L-ornithin L-aspartat'=4) ('44=truyen dich NaCl 0.9%/99=acid tranexamic 250mg'=4) ('44=truyen dich NaCl/99=L-ornithin L-aspartat'=4)

('44=truyen dich( NaCl 0.9%)/99=tang cuong dinh duong'=4) ('44=truyen dich(glucose 5%)/99=bidogozin'=4) ('44=truyen dich(NaCl 0,9%, acid tranexamic)'=4)

('44=truyen dich/99=etamsylate'=4) ('44=truyen dich/99=tho oxy'=4) ('44=truyen dich/99=tho oxy am,diaphilin 4.8%'=4) ('44=truyen dichNaCl 0.9%/99=levelamy'=4)

('44=truyen dichNaCl 9%/99=L-ornithine-L-aspartate'=4) ('99= celecoxib+Acetaminophen'=4) ('99= cimetidine'=4) ('99= magie B6'=4) ('99= omeprazol'=4) ('99= potassium chlorid'=4)

('99= tanakan'=4) ('99=3B'=4) ('99=acetaminophen/oxomemazin/guaifenesin'=4) ('99=acid tranexamic'=4) ('99=adrenoxyl'=4) ('99=allopurinol'=4) ('99=allopurinol, diclofenac'=4)

('99=Almitrine+Raubasine'=4) ('99=alpha chymotrypsin;lansoprazol'=4) ('99=alphadeka'=4) ('99=aluminum hydroxide, magnesium hydroxide, simethicone'=4) ('99=aluminum hydroxide, magnesium hydroxide, Simethicone'=4)

('99=aluminum hydroxide/lasoprazol'=4) ('99=aluminumhydroxid/magie hydroxid/simethicon/ omeprazol'=4) ('99=amiplex,cefpodoxim,azithromycin'=4) ('99=amoxicilin; metronidazol;attapulgit'=4) ('99=arginin'=4)

('99=arginin, clophenyramin'=4) ('99=atropin,attapulgite; omeprazol'=4) ('99=attapulgit'=4) ('99=attapulgit, omeprazol'=4) ('99=azithromycin'=4) ('99=B6'=4) ('99=bcomlex'=4)

('99=biphenyl dimethyl dicarboxylate'=4) ('99=boganic'=4) ('99=bromhexin hydroclorid'=4) ('99=cefmetazol'=4) ('99=cefotaxim'=4) ('99=cerebrolysin/ diazepam/ amlodipin'=4)

('99=Cimetidin + Gastropulgit'=4) ('99=cimetidine'=4) ('99=colchicin'=4) ('99=cycloferon,nghi ngoi'=4) ('99=diclofenac'=4) ('99=diclofenac, allopurinol'=4) ('99=diclofenac, omeprazol'=4)

('99=diclofenac; omeprazol'=4) ('99=Duxil'=4) ('99=duxil 40mg'=4) ('99=efferangan'=4) ('99=erythropoietin; calcium-DL-3-methyl-2-oxo-valerate'=4) ('99=gastropulgit'=4)

('99=ginko biloba'=4) ('99=ginkobiloba'=4) ('99=gumas'=4) ('99=hoat huyet duong nao'=4) ('99=insulin , dieu chinh che do an'=4) ('99=kaleorid'=4) ('99=kali clorid'=4) ('99=KCl'=4)

('99=L-ornithin L-aspartat'=4) ('99=levothyroxin'=4) ('99=loperamid'=4) ('99=loperamid + oresol'=4) ('99=meloxicam'=4) ('99=meloxicam, omeprazol'=4) ('99=meloxicam, uong nhieu nuoc'=4)

('99=meloxicam; omeprazol; uong nhieu nuoc'=4) ('99=meloxicam/ omeprazol'=4) ('99=meloxicam/attapulgite/ omeprazol'=4) ('99=meloxicam/omeprazol'=4) ('99=metoclopramid'=4)

('99=morphin;dimedrol; tho oxy; acid transamid; terpin codein'=4) ('99=NaCl 9%/ cycloserin 12.5%'=4) ('99=NaCl/acid tranesamic'=4) ('99=omeorazole'=4) ('99=omeprazol'=4)

('99=Omeprazol'=4) ('99=omeprazol + gastropulgit'=4) ('99=omeprazol + Gastropulgit'=4) ('99=omeprazol, cetirizin'=4) ('99=omeprazol/attapulgit'=4) ('99=omeprazol/gastopulgit'=4)

('99=omeprazol/gastropulgit'=4) ('99=omeprazole'=4) ('99=paracetamol'=4) ('99=paracetamol, ORS'=4) ('99=piracetam'=4) ('99=Piracetam + Gingo biloba'=4)

('99=potassium chlorid'=4) ('99=rotindin'=4) ('99=rotundin'=4) ('99=Rotundin'=4) ('99=silymarin'=4) ('99=sulpirid'=4) ('99=tanakan'=4) ('99=theo doi; silymarin'=4) ('99=tho oxy'=6)

('99=tho oxy am'=6) ('99=tho oxy tai nha'=4) ('99=thyroxin'=4) ('99=trang vi khang'=4) ('99=truyen dich; cam mau; tho oxy'=6) ('99=truyen khoi tieu cau'=6)

('99=uong nhieu nuoc, omeprazol,meloxicam'=4) ('99=uong nhieu nuoc/ Calcium-DL-3-methyl-2-oxo-valerate'=4) ('99=vat ly tri lieu'=4) ('99=vien an than'=4) ('99=Vitamin 3B'=4)

('99=vitamin b6'=4) ('99=vitamin B6'=4) ('99=VTM 3B/botecgan/L-orniithine L-aspartate'=4) ('99=VTM B6'=4) ('adrenalin'=4) ('arginin'=4) ('dimedrol'=4) ('glucose, levelamy'=4)

('omeprazol'=4) ('omeprazol+ vitamin B6'=4) ('silymarin'=4) ('vitamin B1'=4) ('vitamin B6'=4) ('5=chay than'=5) ('6=chuyen vien'=6) ('6=chuyen vien/99=tanakan'=6)

('99= chuyen vien nhung benh nhan khong nhap vien'=6) ('99= nhập viện điều trị'=6) ('99=benh nhan khong dong y nhap vien'=6) ('99=hướng dẫn bệnh nhân đi khám chuyên khoa'=6)

('99=hướng dẫn bệnh nhân khám mắt'=6) ('99=hướng đẫn bn nhập viện'=6) ('99=hướng dẫn nhập viện'=6) ('99=nhap vien'=6) ('99=yêu cầu bệnh nhân nhập viện'=6) ('7=khong ro'=99)

('99='=99) ('99= nghi ngoi/tang cuong dinh duong'=7) ('99=an chuoi tieu'=7) ('99=chuom'=7) ('99=chuom, thay doi vi tri'=7) ('99=chuom/ thay doi vi tri'=7) ('99=chuom/thay doi vi tri'=7)

('99=chuom/thay doi vi tri tiem'=7) ('99=chup cot song'=6) ('99=chuyen vien nhung benh nhan khong chap nhan va gia dinh khong co dieu kien'=6)

('99=chuyen vien nhung benh nhankhong nhap vien'=6) ('99=dieu chinh che do an'=7) ('99=khac'=7) ('99=kiem soat duong mau'=7) ('99=kiem tra huyet ap'=7) ('99=nghi ngoi'=7)

('99=nghi ngoi, tang cuong dinh duong'=7) ('99=nghi ngoi/ tang cuong dinh duong'=7) ('99=nghi ngoi/tang cuong dinh duong'=7) ('99=ngu bu vao ban ngay'=7) ('99=tam sen'=7)

('99=thay doi vi tri tiem'=7) ('99=theo doi'=7) ('99=theo doi them'=7) ('99=tu van tam ly'=7) ('99=uong nhieu nuoc'=7) ('991=hướng dẫn CBYT xã, người nhà theo dõi bn'=7)

('991=uong nhieu nuoc'=7) (else=99) into xutribc2a.

EXECUTE.

recode xutribc3 (CONVERT) ('0=khong xu tri'=0) ('1=giam lieu'=1) ('1=giam lieu kanamycin'=1) ('1=giam lieu Km, PZA/99=silimarin'=1) ('2=tam ngung'=2) ('2=tam ngung EMB'=2)

('2=tam ngung kanamycin/99=VTM B6/tanakan/6=chuyen vien'=2) ('2=tam ngung prothionamid'=2) ('2=tam ngung PZA'=2) ('3=doi thuoc'=3) ('41=khang histamin'=4)

('44=glucose + ringerlactat'=4) ('44=truyen dich'=4) ('44=truyen dich gluciose 5%/99=L-ornithin L-aspartat,nghi ngoi,tang cuong dinh duong'=4) ('44=truyen dich glucose 5%/99=L-ornithin L-aspartat'=4)

('44=truyen dich NaCl'=4) ('44=truyen dich/99=L-ornithine-L-Aspartate'=4) ('44=truyen dich/99=L-ornithine-L-Aspartate; amiflex'=4) ('44=truyen dich/bo gan'=4) ('45=thuoc boi ngoai xanh methylen'=4)

('99= calcium-DL-3-methyl-2-oxo-valerate'=4) ('99= calcium-DL-3-methyl-2-oxo-valerate;uong nhieu nuoc'=4) ('99= sulfamethoxazol/trimethoprim; cefixim'=4) ('99= terpin codein'=4)

('99=aluminum hydroxide/lasoprazol'=4) ('99=ambroxol'=4) ('99=amoxicilin/clarythromycin/alpha chymotrypsin'=4) ('99=arginin'=4) ('99=attapulgit, omeprazol'=4)

('99=azithromycin/ terpin codein'=4) ('99=biphenyl dimethyl dicarboxylate/uong nhieu nuoc'=4) ('99=bo sung calci'=4) ('99=boganic'=4) ('99=Calcium-DL-3-methyl-2-oxo-valerat'=4)

('99=calcium-DL-3-methyl-2-oxo-valerate'=4) ('99=calcium-DL-3-methyl-2-oxo-valerate; erythropoietin'=4) ('99=cefixim'=4) ('99=cefixim 0,5g'=4) ('99=cefixim 200mg/ clarithromycin 500mg'=4) ('99=cefotaxim'=4)

('99=Cefotaxim/Azilid'=4) ('99=ceftazidim/VTM 3B/hatabtrypsin'=4) ('99=colchicin'=4) ('99=diazepam'=4) ('99=erythropoietin'=4) ('99=erythropoietin/ calcium-DL-3-methyl-2-oxo-valerate;'=4) ('99=furosemid/kali oxid'=4)

('99=furosemid/kali oxid/calcium-DL-3-methyl-2-oxo-valerate'=4) ('99=gliclazid/ metformin/dieu chinh che do an'=4) ('99=ibuprofen/theo doi'=4) ('99=insulin'=4) ('99=kali clorid'=4) ('99=lactomin'=4)

('99=magnesium hydroxide, aluminum hydroxide'=4) ('99=meloxicam'=4) ('99=meloxicam/ omeprazol'=4) ('99=meloxicam/omeprazol'=4) ('99=meloxicam/uong nhieu nuoc'=4)

('99=metformin'=4) ('99=omeprazol'=4) ('99=omeprazol, cetirizin'=4) ('99=omeprazol/ attapulgit'=4) ('99=paracetamol'=4) ('99=paracetamol,uong nhieu nuoc chanh muoi'=4)

('99=piracetam; VTM B6'=4) ('99=rotundin'=4) ('99=silymarin'=4) ('99=symbicort'=4) ('99=tam sen'=7) ('99=tanakan'=4) ('99=tanakan/ VTM B6'=4) ('99=tho oxy'=6)

('99=uong nhieu nuoc;erythropoietin; calcium-DL-3-methyl-2-oxo-valerate/6=chuyen vien'=6) ('99=uong nhieu nuoc/Calcium-DL-3-methyl-2-oxo-valerat'=4) ('99=VTM 3B/ an chuoi'=4)

('99=VTM B6'=4) ('99=xet nghiem'=6) ('dimedrol'=4) ('meloxicam'=4) ('6=chuyen vien'=6) ('6=chuyen vien/99=dieu chinh che do an'=6) ('6=chuyen vien/99=nghi ngoi'=6)

('6=chuyen vien/99=tanakan'=6) ('7=khong ro'=99) ('99='=99) ('99= an chuoi tieu'=7) ('99= nhap vien'=6) ('99= uong nhieu nuoc'=7) ('99=an chuoi tieu'=7)

('99=an hoa qua/ tang cuong dinh duong'=7) ('99=an it ngot'=7) ('99=an nhieu bua, tang cuong dinh duong'=7) ('99=chuom'=7) ('99=chuom, thay doi vi tri'=7)

('99=chuom/ thay doi vi tri'=7) ('99=chuom/thay doi vi ri tiem'=7) ('99=chup cat lop/ kham tim to/kham tim mach'=6) ('99=dieu chinh che do an'=7)

('99=dieu chinh che do an, thay doi lieu insulin'=7) ('99=dong vien benh nhan'=7) ('99=khac'=99) ('99=kham noi tiet'=6) ('99=kiem soat duong huyet'=7) ('99=nghi ngoi'=7)

('99=nghi ngoi, tang cuong dinh duong'=7) ('99=nghi ngoi; uong nhieu nuoc'=7) ('99=nghi ngoi/ tang cuong dinh duong'=7) ('99=nghi ngoi/tang cuong dinh duong'=7) ('99=sieu am'=7)

('99=tang cuong dinh duong'=7) ('99=thay doi che do an'=7) ('99=thay doi vi tri/ chuom'=7) ('99=thay doi vi tri/chuom'=7) ('99=theo doi them'=7) ('99=tu van dinh duong'=7)

('99=uong nhieu nuoc'=7) ('99=van dong khop goi'=7) ('991=hướng dẫn CBYT xã, người nhà theo dõi bn'=7) ('991=theo doi them'=7) (else=99) into xutribc3a.

EXECUTE.

Recode xutribckhac (CONVERT) ('0=khong xu tri'=0) ('00'=0) ('2=tam ngung'=2) ('2=tam ngung prothionamid'=2) ('3=doi thuoc'=3) ('41=khang histamin'=4) ('44=truyen dich NaCl'=4)

('44=truyen dich(NaCl )'=4) ('52=da day nghi ngo'=4) ('6=chuyen vien'=6) ('7=khong ro'=99) ('99= calcium-DL-3-methyl-2-oxo-valerate'=4) ('99= calcium-DL-3-methyl-2-oxo-valerate, uong nhieu nuoc'=4)

('99= han che do ngot'=7) ('99=an chuoi tieu'=7) ('99=an nhieu bua'=7) ('99=an uong tang cuong dinh duong'=7) ('99=arginin'=4) ('99=azithromycin'=4)

('99=calcium/6=chuyen vien'=6) ('99=che do an'=7) ('99=chuom'=7) ('99=chuom, thay doi vi tri'=7) ('99=chuom/ thay doi vi tri'=7) ('99=dieu chinh che do an'=7)

('99=fluconazol'=4) ('99=giam lieu'=1) ('99=kaliclorid'=4) ('99=khac'=99) ('99=kham chuyen khoa'=6) ('99=kiem soat che do an'=7) ('99=kiem soat duong mau; acid tranexamid'=4)

('99=meloxicam'=4) ('99=nghi ngoi'=7) ('99=nghi ngoi/ tang cuong dinh duong'=7) ('99=omeprazol'=4) ('99=silymarin'=4) ('99=silymarin/ an nhieu bua'=4) ('99=tanakan'=4)

('99=tanakan/ nghi ngoi'=4) ('99=tang cuong mien dich'=4) ('99=terpin codein'=4) ('99=thay doi che do an'=7) ('99=thay doi vi tri/chuom'=7) ('99=theo doi them'=7)

('99=tho oxy'=6) ('99=uong nhieu nuoc'=7) ('99=vitamin'=4) ('paracetamol, ringerlactat'=4) ('uống nhiều nước'=7) (else=99) INTO xutribckhaca.

execute.

VALUE LABELS xutribc1a

0 'khong xu tri' 1 'giam lieu' 2 'tam ngung va hoac dung thuoc' 3 'doi thuoc' 4 'dung thuoc trieu chung' 5 'chay than' 6 'nhap vien'

7 'xu tri khac' 99 'khong co thong tin'.

EXECUTE.

VALUE LABELS xutribc2a

0 'khong xu tri' 1 'giam lieu' 2 'tam ngung va hoac du*ng thuoc' 3 'doi thuoc' 4 'dung thuoc trieu chung' 5 'chay than' 6 'nhap vien'

7 'xu tri khac' 99 'khong co thong tin'.

EXECUTE.

VALUE LABELS xutribc3a

0 'khong xu tri' 1 'giam lieu' 2 'tam ngung va hoac du*ng thuoc' 3 'doi thuoc' 4 'dung thuoc trieu chung' 5 'chay than' 6 'nhap vien'

7 'xu tri khac' 99 'khong co thong tin'.

EXECUTE.

Value labels xutribckhaca

0 'khong xu tri' 1 'giam lieu' 2 'tam ngung va hoac du*ng thuoc' 3 'doi thuoc' 4 'dung thuoc trieu chung' 5 'chay than' 6 'nhap vien' 7 'xu tri khac' 99 'khong co thong tin'.

EXECUTE.

***Xác định mức độ nghiêm trọng, gộp biến mức độ và biến xử trí ***

Do if mucdobc1a = 1.

compute nghiemtrong1=1.

else if mucdobc1a=2.

compute nghiemtrong1=2.

else if mucdobc1a=4.

compute nghiemtrong1=3.

else if mucdobc1a=3.

compute nghiemtrong1=4.

else if xutribc1a=6.

compute nghiemtrong1=5.

else if xutribc1a=2.

compute nghiemtrong1=6.

else if xutribc1a=3.

compute nghiemtrong1=7.

else if xutribc1a=1.

compute nghiemtrong1= 8.

else if xutribc1a=4.

compute nghiemtrong1=9.

else if xutribc1a=5.

compute nghiemtrong1=10.

else if xutribc1a=7.

compute nghiemtrong1=11.

else if mucdobc1a=0.

compute nghiemtrong1=12.

else if xutribc1a=0.

compute nghiemtrong1=13.

else if mucdobc1a=99 OR mucdobc1a=5.

compute nghiemtrong1=14.

end if.

Recode nghiemtrong1 (missing =99).

execute.

Value labels nghiemtrong1

1 'tu vong' 2 'tinh mang' 3 'tan tat' 4 'keo dai nam vien' 5 'chi dinh nhap vien' 6 'tam dung hoac dung thuoc' 7 'doi thuoc' 8 'giam lieu thuoc lao' 9 'dung thuoc tri trieu chung' 10 'chay than' 11 'xu tri khac'

12 'khong nghiem trong' 14 'chua phan loai' 13 'khong xu tri'.

EXECUTE.

Do if mucdobc2a = 1.

compute nghiemtrong2=1.

else if mucdobc2a=2.

compute nghiemtrong2=2.

else if mucdobc2a=4.

compute nghiemtrong2=3.

else if mucdobc2a=3.

compute nghiemtrong2=4.

else if xutribc2a=6.

compute nghiemtrong2=5.

else if xutribc2a=2.

compute nghiemtrong2=6.

else if xutribc2a=3.

compute nghiemtrong2=7.

else if xutribc2a=1.

compute nghiemtrong2= 8.

else if xutribc2a=4.

compute nghiemtrong2=9.

else if xutribc2a=5.

compute nghiemtrong2=10.

else if xutribc2a=7.

compute nghiemtrong2=11.

else if mucdobc2a=0.

compute nghiemtrong2=12.

else if xutribc2a=0.

compute nghiemtrong2=13.

else if mucdobc2a=99 OR mucdobc2a=5.

compute nghiemtrong2=14.

end if.

Recode nghiemtrong2 (missing = 99).

execute.

Value labels nghiemtrong2

1 'tu vong' 2 'tinh mang' 3 'tan tat' 4 'keo dai nam vien' 5 'chi dinh nhap vien' 6 'tam dung hoac dung thuoc' 7 'doi thuoc' 8 'giam lieu thuoc lao' 9 'dung thuoc tri trieu chung' 10 'chay than' 11 'xu tri khac'

12 'khong nghiem trong' 14 'chua phan loai' 13 'khong xu tri'.

EXECUTE.

Do if mucdobc3a = 1.

compute nghiemtrong3=1.

else if mucdobc3a=2.

compute nghiemtrong3=2.

else if mucdobc3a=4.

compute nghiemtrong3=3.

else if mucdobc3a=3.

compute nghiemtrong3=4.

else if xutribc3a=6.

compute nghiemtrong3=5.

else if xutribc3a=2.

compute nghiemtrong3=6.

else if xutribc3a=3.

compute nghiemtrong3=7.

else if xutribc3a=1.

compute nghiemtrong3= 8.

else if xutribc3a=4.

compute nghiemtrong3=9.

else if xutribc3a=5.

compute nghiemtrong3=10.

else if xutribc3a=7.

compute nghiemtrong3=11.

else if mucdobc3a=0.

compute nghiemtrong3=12.

else if xutribc3a=0.

compute nghiemtrong3=13.

else if mucdobc3a=99 OR mucdobc3a=5.

compute nghiemtrong3=14.

end if.

Recode nghiemtrong3 (missing = 99).

execute.

Value labels nghiemtrong3

1 'tu vong' 2 'tinh mang' 3 'tan tat' 4 'keo dai nam vien' 5 'chi dinh nhap vien' 6 'tam dung hoac dung thuoc' 7 'doi thuoc' 8 'giam lieu thuoc lao' 9 'dung thuoc tri trieu chung' 10 'chay than' 11 'xu tri khac'

12 'khong nghiem trong' 14 'chua phan loai' 13 'khong xu tri'.

EXECUTE.

Do if mucdobckhaca = 1.

compute nghiemtrongkhac=1.

else if mucdobckhaca=2.

compute nghiemtrongkhac=2.

else if mucdobckhaca=4.

compute nghiemtrongkhac=3.

else if mucdobckhaca=3.

compute nghiemtrongkhac=4.

else if xutribckhaca=6.

compute nghiemtrongkhac=5.

else if xutribckhaca=2.

compute nghiemtrongkhac=6.

else if xutribckhaca=3.

compute nghiemtrongkhac=7.

else if xutribckhaca=1.

compute nghiemtrongkhac= 8.

else if xutribckhaca=4.

compute nghiemtrongkhac=9.

else if xutribckhaca=5.

compute nghiemtrongkhac=10.

else if xutribckhaca=7.

compute nghiemtrongkhac=11.

else if mucdobckhaca=0.

compute nghiemtrongkhac=12.

else if xutribckhaca=0.

compute nghiemtrongkhac=13.

else if mucdobckhaca=99 OR mucdobckhaca=5.

compute nghiemtrongkhac=14.

end if.

Recode nghiemtrongkhac (missing = 99).

execute.

Value labels nghiemtrongkhac

1 'tu vong' 2 'tinh mang' 3 'tan tat' 4 'keo dai nam vien' 5 'chi dinh nhap vien' 6 'tam dung hoac dung thuoc' 7 'doi thuoc' 8 'giam lieu thuoc lao' 9 'dung thuoc tri trieu chung' 10 'chay than' 11 'xu tri khac'

12 'khong nghiem trong' 14 'chua phan loai' 13 'khong xu tri'.

EXECUTE.

*** Thiet lap thoi gian theo doi benh nhan***

SORT CASES BY Table1x.ID(A) ngay(A).

COMPUTE tte=(ngay-ngaydau)/86400.

EXECUTE.

FILTER OFF.

USE ALL.

SELECT IF (tte>=0 OR tte<1000).

EXECUTE.

do if (Table1x.ID=lag(Table1x.ID)).

compute interval=tte-lag(tte).

else.

compute interval=0.

end if.

***Thiet lap bien so bien co va muc do nghiem trong tuong ung***

***NON***

do if (bc1a=1).

compute non=1.

compute mdnon=nghiemtrong1.

else if (bc2a=1).

compute non=1.

compute mdnon=nghiemtrong2.

else if (bc3a=1).

compute non=1.

compute mdnon=nghiemtrong3.

else if (bckhaca=1).

compute non=1.

compute mdnon=nghiemtrongkhac.

ELSE.

Compute non=0.

compute mdnon=99.

end if.

Value labels mdnon

1 'tu vong' 2 'tinh mang' 3 'tan tat' 4 'keo dai nam vien' 5 'chi dinh nhap vien' 6 'tam dung hoac dung thuoc' 7 'doi thuoc' 8 'giam lieu thuoc lao' 9 'dung thuoc tri trieu chung' 10 'chay than' 11 'xu tri khac'

12 'khong nghiem trong' 14 'chua phan loai' 13 'khong xu tri'.

EXECUTE.

Do IF ($CASENUM=1).

compute nonkp=non.

compute mdnonkp=mdnon.

compute tnon=tte.

ELSE IF (Table1x.ID = LAG(Table1x.ID)) AND non=1 AND mdnon<lag(mdnonkp).

compute nonkp=non.

compute mdnonkp=mdnon.

compute tnon=tte.

ELSE IF (Table1x.ID=lag(Table1x.ID) AND non =1 AND mdnon>=lag(mdnonkp)).

COMPUTE nonkp=non.

compute mdnonkp=lag(mdnonkp).

compute tnon=lag(tnon).

else if (Table1x.ID=lag(Table1x.ID) AND non =0 AND lag(nonkp)=1).

compute nonkp=lag(nonkp).

compute mdnonkp=lag(mdnonkp).

compute tnon=lag(tnon).

ELSE.

compute nonkp=non.

compute mdnonkp=mdnon.

compute tnon=tte.

END IF.

if ($CASENUM=1) and nonkp=1 tnona=tnon.

if (Table1x.ID<>lag(Table1x.ID)) and nonkp=1 tnona=tnon.

if (Table1x.ID=lag(Table1x.ID)) and nonkp=1 and lag(nonkp)=0 tnona=tnon.

if (Table1x.ID=lag(Table1x.ID)) and nonkp=1 and lag(nonkp)=1 tnona=lag(tnona).

if tnona>0 tnon=tnona.

Value labels mdnonkp

1 'tu vong' 2 'tinh mang' 3 'tan tat' 4 'keo dai nam vien' 5 'chi dinh nhap vien' 6 'tam dung hoac dung thuoc' 7 'doi thuoc' 8 'giam lieu thuoc lao' 9 'dung thuoc tri trieu chung' 10 'chay than' 11 'xu tri khac'

12 'khong nghiem trong' 14 'chua phan loai' 13 'khong xu tri'.

EXECUTE.

***TIEU CHAY***

do if (bc1a=2).

compute tieuchay=1.

compute mdtieuchay=nghiemtrong1.

else if (bc2a=2).

compute tieuchay=1.

compute mdtieuchay=nghiemtrong2.

else if (bc3a=2).

compute tieuchay=1.

compute mdtieuchay=nghiemtrong3.

else if (bckhaca=2).

compute tieuchay=1.

compute mdtieuchay=nghiemtrongkhac.

ELSE.

Compute tieuchay=0.

compute mdtieuchay=99.

end if.

Value labels mdtieuchay

1 'tu vong' 2 'tinh mang' 3 'tan tat' 4 'keo dai nam vien' 5 'chi dinh nhap vien' 6 'tam dung hoac dung thuoc' 7 'doi thuoc' 8 'giam lieu thuoc lao' 9 'dung thuoc tri trieu chung' 10 'chay than' 11 'xu tri khac'

12 'khong nghiem trong' 14 'chua phan loai' 13 'khong xu tri'.

EXECUTE.

Do IF ($CASENUM=1).

compute tieuchaykp=tieuchay.

compute mdtieuchaykp=mdtieuchay.

compute ttieuchay=tte.

ELSE IF (Table1x.ID = LAG(Table1x.ID)) AND tieuchay=1 AND mdtieuchay<lag(mdtieuchaykp).

compute tieuchaykp=tieuchay.

compute mdtieuchaykp=mdtieuchay.

compute ttieuchay=tte.

ELSE IF (Table1x.ID=lag(Table1x.ID) AND tieuchay =1 AND mdtieuchay>=lag(mdtieuchaykp)).

COMPUTE tieuchaykp=tieuchay.

compute mdtieuchaykp=lag(mdtieuchaykp).

compute ttieuchay=lag(ttieuchay).

else if (Table1x.ID=lag(Table1x.ID) AND tieuchay =0 AND lag(tieuchaykp)=1).

compute tieuchaykp=lag(tieuchaykp).

compute mdtieuchaykp=lag(mdtieuchaykp).

compute ttieuchay=lag(ttieuchay).

ELSE.

compute tieuchaykp=tieuchay.

compute mdtieuchaykp=mdtieuchay.

compute ttieuchay=tte.

END IF.

if ($CASENUM=1) and tieuchaykp=1 ttieuchaya=ttieuchay.

if (Table1x.ID<>lag(Table1x.ID)) and tieuchaykp=1 ttieuchaya=ttieuchay.

if (Table1x.ID=lag(Table1x.ID)) and tieuchaykp=1 and lag(tieuchaykp)=0 ttieuchaya=ttieuchay.

if (Table1x.ID=lag(Table1x.ID)) and tieuchaykp=1 and lag(tieuchaykp)=1 ttieuchaya=lag(ttieuchaya).

if ttieuchaya>0 ttieuchay=ttieuchaya.

Value labels mdtieuchaykp

1 'tu vong' 2 'tinh mang' 3 'tan tat' 4 'keo dai nam vien' 5 'chi dinh nhap vien' 6 'tam dung hoac dung thuoc' 7 'doi thuoc' 8 'giam lieu thuoc lao' 9 'dung thuoc tri trieu chung' 10 'chay than' 11 'xu tri khac'

12 'khong nghiem trong' 14 'chua phan loai' 13 'khong xu tri'.

EXECUTE.

***DAU BUNG***

do if (bc1a=3).

compute daubung=1.

compute mddaubung=nghiemtrong1.

else if (bc2a=3).

compute daubung=1.

compute mddaubung=nghiemtrong2.

else if (bc3a=3).

compute daubung=1.

compute mddaubung=nghiemtrong3.

else if (bckhaca=3).

compute daubung=1.

compute mddaubung=nghiemtrongkhac.

ELSE.

Compute daubung=0.

compute mddaubung=99.

end if.

Value labels mddaubung

1 'tu vong' 2 'tinh mang' 3 'tan tat' 4 'keo dai nam vien' 5 'chi dinh nhap vien' 6 'tam dung hoac dung thuoc' 7 'doi thuoc' 8 'giam lieu thuoc lao' 9 'dung thuoc tri trieu chung' 10 'chay than' 11 'xu tri khac'

12 'khong nghiem trong' 14 'chua phan loai' 13 'khong xu tri'.

EXECUTE.

Do IF ($CASENUM=1).

compute daubungkp=daubung.

compute mddaubungkp=mddaubung.

compute tdaubung=tte.

ELSE IF (Table1x.ID = LAG(Table1x.ID)) AND daubung=1 AND mddaubung<lag(mddaubungkp).

compute daubungkp=daubung.

compute mddaubungkp=mddaubung.

compute tdaubung=tte.

ELSE IF (Table1x.ID=lag(Table1x.ID) AND daubung =1 AND mddaubung>=lag(mddaubungkp)).

COMPUTE daubungkp=daubung.

compute mddaubungkp=lag(mddaubungkp).

compute tdaubung=lag(tdaubung).

else if (Table1x.ID=lag(Table1x.ID) AND daubung =0 AND lag(daubungkp)=1).

compute daubungkp=lag(daubungkp).

compute mddaubungkp=lag(mddaubungkp).

compute tdaubung=lag(tdaubung).

ELSE.

compute daubungkp=daubung.

compute mddaubungkp=mddaubung.

compute tdaubung=tte.

END IF.

if ($CASENUM=1) and daubungkp=1 tdaubunga=tdaubung.

if (Table1x.ID<>lag(Table1x.ID)) and daubungkp=1 tdaubunga=tdaubung.

if (Table1x.ID=lag(Table1x.ID)) and daubungkp=1 and lag(daubungkp)=0 tdaubunga=tdaubung.

if (Table1x.ID=lag(Table1x.ID)) and daubungkp=1 and lag(daubungkp)=1 tdaubunga=lag(tdaubunga).

if tdaubunga>0 tdaubung=tdaubunga.

Value labels mddaubungkp

1 'tu vong' 2 'tinh mang' 3 'tan tat' 4 'keo dai nam vien' 5 'chi dinh nhap vien' 6 'tam dung hoac dung thuoc' 7 'doi thuoc' 8 'giam lieu thuoc lao' 9 'dung thuoc tri trieu chung' 10 'chay than' 11 'xu tri khac'

12 'khong nghiem trong' 14 'chua phan loai' 13 'khong xu tri'.

EXECUTE.

***CHAN ANH***

do if (bc1a=4).

compute chanan=1.

compute mdchanan=nghiemtrong1.

else if (bc2a=4).

compute chanan=1.

compute mdchanan=nghiemtrong2.

else if (bc3a=4).

compute chanan=1.

compute mdchanan=nghiemtrong3.

else if (bckhaca=4).

compute chanan=1.

compute mdchanan=nghiemtrongkhac.

ELSE.

Compute chanan=0.

compute mdchanan=99.

end if.

Value labels mdchanan

1 'tu vong' 2 'tinh mang' 3 'tan tat' 4 'keo dai nam vien' 5 'chi dinh nhap vien' 6 'tam dung hoac dung thuoc' 7 'doi thuoc' 8 'giam lieu thuoc lao' 9 'dung thuoc tri trieu chung' 10 'chay than' 11 'xu tri khac'

12 'khong nghiem trong' 14 'chua phan loai' 13 'khong xu tri'.

EXECUTE.

Do IF ($CASENUM=1).

compute chanankp=chanan.

compute mdchanankp=mdchanan.

compute tchanan=tte.

ELSE IF (Table1x.ID = LAG(Table1x.ID)) AND chanan=1 AND mdchanan<lag(mdchanankp).

compute chanankp=chanan.

compute mdchanankp=mdchanan.

compute tchanan=tte.

ELSE IF (Table1x.ID=lag(Table1x.ID) AND chanan =1 AND mdchanan>=lag(mdchanankp)).

COMPUTE chanankp=chanan.

compute mdchanankp=lag(mdchanankp).

compute tchanan=lag(tchanan).

else if (Table1x.ID=lag(Table1x.ID) AND chanan =0 AND lag(chanankp)=1).

compute chanankp=lag(chanankp).

compute mdchanankp=lag(mdchanankp).

compute tchanan=lag(tchanan).

ELSE.

compute chanankp=chanan.

compute mdchanankp=mdchanan.

compute tchanan=tte.

END IF.

if ($CASENUM=1) and chanankp=1 tchanana=tchanan.

if (Table1x.ID<>lag(Table1x.ID)) and chanankp=1 tchanana=tchanan.

if (Table1x.ID=lag(Table1x.ID)) and chanankp=1 and lag(chanankp)=0 tchanana=tchanan.

if (Table1x.ID=lag(Table1x.ID)) and chanankp=1 and lag(chanankp)=1 tchanana=lag(tchanana).

if tchanana>0 tchanan=tchanana.

Value labels mdchanankp

1 'tu vong' 2 'tinh mang' 3 'tan tat' 4 'keo dai nam vien' 5 'chi dinh nhap vien' 6 'tam dung hoac dung thuoc' 7 'doi thuoc' 8 'giam lieu thuoc lao' 9 'dung thuoc tri trieu chung' 10 'chay than' 11 'xu tri khac'

12 'khong nghiem trong' 14 'chua phan loai' 13 'khong xu tri'.

EXECUTE.

***DA DAY XAC DINH***

do if (bc1a=51).

compute dadayxd=1.

compute mddadayxd=nghiemtrong1.

else if (bc2a=51).

compute dadayxd=1.

compute mddadayxd=nghiemtrong2.

else if (bc3a=51).

compute dadayxd=1.

compute mddadayxd=nghiemtrong3.

else if (bckhaca=51).

compute dadayxd=1.

compute mddadayxd=nghiemtrongkhac.

ELSE.

Compute dadayxd=0.

compute mddadayxd=99.

end if.

Value labels mddadayxd

1 'tu vong' 2 'tinh mang' 3 'tan tat' 4 'keo dai nam vien' 5 'chi dinh nhap vien' 6 'tam dung hoac dung thuoc' 7 'doi thuoc' 8 'giam lieu thuoc lao' 9 'dung thuoc tri trieu chung' 10 'chay than' 11 'xu tri khac'

12 'khong nghiem trong' 14 'chua phan loai' 13 'khong xu tri'.

EXECUTE.

Do IF ($CASENUM=1).

compute dadayxdkp=dadayxd.

compute mddadayxdkp=mddadayxd.

compute tdadayxd=tte.

ELSE IF (Table1x.ID = LAG(Table1x.ID)) AND dadayxd=1 AND mddadayxd<lag(mddadayxdkp).

compute dadayxdkp=dadayxd.

compute mddadayxdkp=mddadayxd.

compute tdadayxd=tte.

ELSE IF (Table1x.ID=lag(Table1x.ID) AND dadayxd =1 AND mddadayxd>=lag(mddadayxdkp)).

COMPUTE dadayxdkp=dadayxd.

compute mddadayxdkp=lag(mddadayxdkp).

compute tdadayxd=lag(tdadayxd).

else if (Table1x.ID=lag(Table1x.ID) AND dadayxd =0 AND lag(dadayxdkp)=1).

compute dadayxdkp=lag(dadayxdkp).

compute mddadayxdkp=lag(mddadayxdkp).

compute tdadayxd=lag(tdadayxd).

ELSE.

compute dadayxdkp=dadayxd.

compute mddadayxdkp=mddadayxd.

compute tdadayxd=tte.

END IF.

if ($CASENUM=1) and dadayxdkp=1 tdadayxda=tdadayxd.

if (Table1x.ID<>lag(Table1x.ID)) and dadayxdkp=1 tdadayxda=tdadayxd.

if (Table1x.ID=lag(Table1x.ID)) and dadayxdkp=1 and lag(dadayxdkp)=0 tdadayxda=tdadayxd.

if (Table1x.ID=lag(Table1x.ID)) and dadayxdkp=1 and lag(dadayxdkp)=1 tdadayxda=lag(tdadayxda).

if tdadayxda>0 tdadayxd=tdadayxda.

Value labels mddadayxdkp

1 'tu vong' 2 'tinh mang' 3 'tan tat' 4 'keo dai nam vien' 5 'chi dinh nhap vien' 6 'tam dung hoac dung thuoc' 7 'doi thuoc' 8 'giam lieu thuoc lao' 9 'dung thuoc tri trieu chung' 10 'chay than' 11 'xu tri khac'

12 'khong nghiem trong' 14 'chua phan loai' 13 'khong xu tri'.

EXECUTE.

***DA DAY NGHI NGO***

do if (bc1a=52).

compute dadaynn=1.

compute mddadaynn=nghiemtrong1.

else if (bc2a=52).

compute dadaynn=1.

compute mddadaynn=nghiemtrong2.

else if (bc3a=52).

compute dadaynn=1.

compute mddadaynn=nghiemtrong3.

else if (bckhaca=52).

compute dadaynn=1.

compute mddadaynn=nghiemtrongkhac.

ELSE.

Compute dadaynn=0.

compute mddadaynn=99.

end if.

Value labels mddadaynn

1 'tu vong' 2 'tinh mang' 3 'tan tat' 4 'keo dai nam vien' 5 'chi dinh nhap vien' 6 'tam dung hoac dung thuoc' 7 'doi thuoc' 8 'giam lieu thuoc lao' 9 'dung thuoc tri trieu chung' 10 'chay than' 11 'xu tri khac'

12 'khong nghiem trong' 14 'chua phan loai' 13 'khong xu tri'.

EXECUTE.

Do IF ($CASENUM=1).

compute dadaynnkp=dadaynn.

compute mddadaynnkp=mddadaynn.

compute tdadaynn=tte.

ELSE IF (Table1x.ID = LAG(Table1x.ID)) AND dadaynn=1 AND mddadaynn<lag(mddadaynnkp).

compute dadaynnkp=dadaynn.

compute mddadaynnkp=mddadaynn.

compute tdadaynn=tte.

ELSE IF (Table1x.ID=lag(Table1x.ID) AND dadaynn =1 AND mddadaynn>=lag(mddadaynnkp)).

COMPUTE dadaynnkp=dadaynn.

compute mddadaynnkp=lag(mddadaynnkp).

compute tdadaynn=lag(tdadaynn).

else if (Table1x.ID=lag(Table1x.ID) AND dadaynn =0 AND lag(dadaynnkp)=1).

compute dadaynnkp=lag(dadaynnkp).

compute mddadaynnkp=lag(mddadaynnkp).

compute tdadaynn=lag(tdadaynn).

ELSE.

compute dadaynnkp=dadaynn.

compute mddadaynnkp=mddadaynn.

compute tdadaynn=tte.

END IF.

if ($CASENUM=1) and dadaynnkp=1 tdadaynna=tdadaynn.

if (Table1x.ID<>lag(Table1x.ID)) and dadaynnkp=1 tdadaynna=tdadaynn.

if (Table1x.ID=lag(Table1x.ID)) and dadaynnkp=1 and lag(dadaynnkp)=0 tdadaynna=tdadaynn.

if (Table1x.ID=lag(Table1x.ID)) and dadaynnkp=1 and lag(dadaynnkp)=1 tdadaynna=lag(tdadaynna).

if tdadaynna>0 tdadaynn=tdadaynna.

Value labels mddadaynnkp

1 'tu vong' 2 'tinh mang' 3 'tan tat' 4 'keo dai nam vien' 5 'chi dinh nhap vien' 6 'tam dung hoac dung thuoc' 7 'doi thuoc' 8 'giam lieu thuoc lao' 9 'dung thuoc tri trieu chung' 10 'chay than' 11 'xu tri khac'

12 'khong nghiem trong' 14 'chua phan loai' 13 'khong xu tri'.

EXECUTE.

***DOC GAN XAC DINH***

do if (bc1a=61).

compute docganxd=1.

compute mddocganxd=nghiemtrong1.

else if (bc2a=61).

compute docganxd=1.

compute mddocganxd=nghiemtrong2.

else if (bc3a=61).

compute docganxd=1.

compute mddocganxd=nghiemtrong3.

else if (bckhaca=61).

compute docganxd=1.

compute mddocganxd=nghiemtrongkhac.

ELSE.

Compute docganxd=0.

compute mddocganxd=99.

end if.

Value labels mddocganxd

1 'tu vong' 2 'tinh mang' 3 'tan tat' 4 'keo dai nam vien' 5 'chi dinh nhap vien' 6 'tam dung hoac dung thuoc' 7 'doi thuoc' 8 'giam lieu thuoc lao' 9 'dung thuoc tri trieu chung' 10 'chay than' 11 'xu tri khac'

12 'khong nghiem trong' 14 'chua phan loai' 13 'khong xu tri'.

EXECUTE.

Do IF ($CASENUM=1).

compute docganxdkp=docganxd.

compute mddocganxdkp=mddocganxd.

compute tdocganxd=tte.

ELSE IF (Table1x.ID = LAG(Table1x.ID)) AND docganxd=1 AND mddocganxd<lag(mddocganxdkp).

compute docganxdkp=docganxd.

compute mddocganxdkp=mddocganxd.

compute tdocganxd=tte.

ELSE IF (Table1x.ID=lag(Table1x.ID) AND docganxd =1 AND mddocganxd>=lag(mddocganxdkp)).

COMPUTE docganxdkp=docganxd.

compute mddocganxdkp=lag(mddocganxdkp).

compute tdocganxd=lag(tdocganxd).

else if (Table1x.ID=lag(Table1x.ID) AND docganxd =0 AND lag(docganxdkp)=1).

compute docganxdkp=lag(docganxdkp).

compute mddocganxdkp=lag(mddocganxdkp).

compute tdocganxd=lag(tdocganxd).

ELSE.

compute docganxdkp=docganxd.

compute mddocganxdkp=mddocganxd.

compute tdocganxd=tte.

END IF.

if ($CASENUM=1) and docganxdkp=1 tdocganxda=tdocganxd.

if (Table1x.ID<>lag(Table1x.ID)) and docganxdkp=1 tdocganxda=tdocganxd.

if (Table1x.ID=lag(Table1x.ID)) and docganxdkp=1 and lag(docganxdkp)=0 tdocganxda=tdocganxd.

if (Table1x.ID=lag(Table1x.ID)) and docganxdkp=1 and lag(docganxdkp)=1 tdocganxda=lag(tdocganxda).

if tdocganxda>0 tdocganxd=tdocganxda.

Value labels mddocganxdkp

1 'tu vong' 2 'tinh mang' 3 'tan tat' 4 'keo dai nam vien' 5 'chi dinh nhap vien' 6 'tam dung hoac dung thuoc' 7 'doi thuoc' 8 'giam lieu thuoc lao' 9 'dung thuoc tri trieu chung' 10 'chay than' 11 'xu tri khac'

12 'khong nghiem trong' 14 'chua phan loai' 13 'khong xu tri'.

EXECUTE.

***DOC GAN NGHI NGO***

do if (bc1a=62).

compute docgannn=1.

compute mddocgannn=nghiemtrong1.

else if (bc2a=62).

compute docgannn=1.

compute mddocgannn=nghiemtrong2.

else if (bc3a=62).

compute docgannn=1.

compute mddocgannn=nghiemtrong3.

else if (bckhaca=62).

compute docgannn=1.

compute mddocgannn=nghiemtrongkhac.

ELSE.

Compute docgannn=0.

compute mddocgannn=99.

end if.

Value labels mddocgannn

1 'tu vong' 2 'tinh mang' 3 'tan tat' 4 'keo dai nam vien' 5 'chi dinh nhap vien' 6 'tam dung hoac dung thuoc' 7 'doi thuoc' 8 'giam lieu thuoc lao' 9 'dung thuoc tri trieu chung' 10 'chay than' 11 'xu tri khac'

12 'khong nghiem trong' 14 'chua phan loai' 13 'khong xu tri'.

EXECUTE.

Do IF ($CASENUM=1).

compute docgannnkp=docgannn.

compute mddocgannnkp=mddocgannn.

compute tdocgannn=tte.

ELSE IF (Table1x.ID = LAG(Table1x.ID)) AND docgannn=1 AND mddocgannn<lag(mddocgannnkp).

compute docgannnkp=docgannn.

compute mddocgannnkp=mddocgannn.

compute tdocgannn=tte.

ELSE IF (Table1x.ID=lag(Table1x.ID) AND docgannn =1 AND mddocgannn>=lag(mddocgannnkp)).

COMPUTE docgannnkp=docgannn.

compute mddocgannnkp=lag(mddocgannnkp).

compute tdocgannn=lag(tdocgannn).

else if (Table1x.ID=lag(Table1x.ID) AND docgannn =0 AND lag(docgannnkp)=1).

compute docgannnkp=lag(docgannnkp).

compute mddocgannnkp=lag(mddocgannnkp).

compute tdocgannn=lag(tdocgannn).

ELSE.

compute docgannnkp=docgannn.

compute mddocgannnkp=mddocgannn.

compute tdocgannn=tte.

END IF.

if ($CASENUM=1) and docgannnkp=1 tdocgannna=tdocgannn.

if (Table1x.ID<>lag(Table1x.ID)) and docgannnkp=1 tdocgannna=tdocgannn.

if (Table1x.ID=lag(Table1x.ID)) and docgannnkp=1 and lag(docgannnkp)=0 tdocgannna=tdocgannn.

if (Table1x.ID=lag(Table1x.ID)) and docgannnkp=1 and lag(docgannnkp)=1 tdocgannna=lag(tdocgannna).

if tdocgannna>0 tdocgannn=tdocgannna.

Value labels mddocgannnkp

1 'tu vong' 2 'tinh mang' 3 'tan tat' 4 'keo dai nam vien' 5 'chi dinh nhap vien' 6 'tam dung hoac dung thuoc' 7 'doi thuoc' 8 'giam lieu thuoc lao' 9 'dung thuoc tri trieu chung' 10 'chay than' 11 'xu tri khac'

12 'khong nghiem trong' 14 'chua phan loai' 13 'khong xu tri'.

EXECUTE.

***DAU DAU***

do if (bc1a=7).

compute daudau=1.

compute mddaudau=nghiemtrong1.

else if (bc2a=7).

compute daudau=1.

compute mddaudau=nghiemtrong2.

else if (bc3a=7).

compute daudau=1.

compute mddaudau=nghiemtrong3.

else if (bckhaca=7).

compute daudau=1.

compute mddaudau=nghiemtrongkhac.

ELSE.

Compute daudau=0.

compute mddaudau=99.

end if.

Value labels mddaudau

1 'tu vong' 2 'tinh mang' 3 'tan tat' 4 'keo dai nam vien' 5 'chi dinh nhap vien' 6 'tam dung hoac dung thuoc' 7 'doi thuoc' 8 'giam lieu thuoc lao' 9 'dung thuoc tri trieu chung' 10 'chay than' 11 'xu tri khac'

12 'khong nghiem trong' 14 'chua phan loai' 13 'khong xu tri'.

EXECUTE.

Do IF ($CASENUM=1).

compute daudaukp=daudau.

compute mddaudaukp=mddaudau.

compute tdaudau=tte.

ELSE IF (Table1x.ID = LAG(Table1x.ID)) AND daudau=1 AND mddaudau<lag(mddaudaukp).

compute daudaukp=daudau.

compute mddaudaukp=mddaudau.

compute tdaudau=tte.

ELSE IF (Table1x.ID=lag(Table1x.ID) AND daudau =1 AND mddaudau>=lag(mddaudaukp)).

COMPUTE daudaukp=daudau.

compute mddaudaukp=lag(mddaudaukp).

compute tdaudau=lag(tdaudau).

else if (Table1x.ID=lag(Table1x.ID) AND daudau =0 AND lag(daudaukp)=1).

compute daudaukp=lag(daudaukp).

compute mddaudaukp=lag(mddaudaukp).

compute tdaudau=lag(tdaudau).

ELSE.

compute daudaukp=daudau.

compute mddaudaukp=mddaudau.

compute tdaudau=tte.

END IF.

if ($CASENUM=1) and daudaukp=1 tdaudaua=tdaudau.

if (Table1x.ID<>lag(Table1x.ID)) and daudaukp=1 tdaudaua=tdaudau.

if (Table1x.ID=lag(Table1x.ID)) and daudaukp=1 and lag(daudaukp)=0 tdaudaua=tdaudau.

if (Table1x.ID=lag(Table1x.ID)) and daudaukp=1 and lag(daudaukp)=1 tdaudaua=lag(tdaudaua).

if tdaudaua>0 tdaudau=tdaudaua.

Value labels mddaudaukp

1 'tu vong' 2 'tinh mang' 3 'tan tat' 4 'keo dai nam vien' 5 'chi dinh nhap vien' 6 'tam dung hoac dung thuoc' 7 'doi thuoc' 8 'giam lieu thuoc lao' 9 'dung thuoc tri trieu chung' 10 'chay than' 11 'xu tri khac'

12 'khong nghiem trong' 14 'chua phan loai' 13 'khong xu tri'.

EXECUTE.

***HOA MAT***

do if (bc1a=8).

compute hoamat=1.

compute mdhoamat=nghiemtrong1.

else if (bc2a=8).

compute hoamat=1.

compute mdhoamat=nghiemtrong2.

else if (bc3a=8).

compute hoamat=1.

compute mdhoamat=nghiemtrong3.

else if (bckhaca=8).

compute hoamat=1.

compute mdhoamat=nghiemtrongkhac.

ELSE.

Compute hoamat=0.

compute mdhoamat=99.

end if.

Value labels mdhoamat

1 'tu vong' 2 'tinh mang' 3 'tan tat' 4 'keo dai nam vien' 5 'chi dinh nhap vien' 6 'tam dung hoac dung thuoc' 7 'doi thuoc' 8 'giam lieu thuoc lao' 9 'dung thuoc tri trieu chung' 10 'chay than' 11 'xu tri khac'

12 'khong nghiem trong' 14 'chua phan loai' 13 'khong xu tri'.

EXECUTE.

Do IF ($CASENUM=1).

compute hoamatkp=hoamat.

compute mdhoamatkp=mdhoamat.

compute thoamat=tte.

ELSE IF (Table1x.ID = LAG(Table1x.ID)) AND hoamat=1 AND mdhoamat<lag(mdhoamatkp).

compute hoamatkp=hoamat.

compute mdhoamatkp=mdhoamat.

compute thoamat=tte.

ELSE IF (Table1x.ID=lag(Table1x.ID) AND hoamat =1 AND mdhoamat>=lag(mdhoamatkp)).

COMPUTE hoamatkp=hoamat.

compute mdhoamatkp=lag(mdhoamatkp).

compute thoamat=lag(thoamat).

else if (Table1x.ID=lag(Table1x.ID) AND hoamat =0 AND lag(hoamatkp)=1).

compute hoamatkp=lag(hoamatkp).

compute mdhoamatkp=lag(mdhoamatkp).

compute thoamat=lag(thoamat).

ELSE.

compute hoamatkp=hoamat.

compute mdhoamatkp=mdhoamat.

compute thoamat=tte.

END IF.

if ($CASENUM=1) and hoamatkp=1 thoamata=thoamat.

if (Table1x.ID<>lag(Table1x.ID)) and hoamatkp=1 thoamata=thoamat.

if (Table1x.ID=lag(Table1x.ID)) and hoamatkp=1 and lag(hoamatkp)=0 thoamata=thoamat.

if (Table1x.ID=lag(Table1x.ID)) and hoamatkp=1 and lag(hoamatkp)=1 thoamata=lag(thoamata).

if thoamata>0 thoamat=thoamata.

Value labels mdhoamatkp

1 'tu vong' 2 'tinh mang' 3 'tan tat' 4 'keo dai nam vien' 5 'chi dinh nhap vien' 6 'tam dung hoac dung thuoc' 7 'doi thuoc' 8 'giam lieu thuoc lao' 9 'dung thuoc tri trieu chung' 10 'chay than' 11 'xu tri khac'

12 'khong nghiem trong' 14 'chua phan loai' 13 'khong xu tri'.

EXECUTE.

***CO GIAT***

do if (bc1a=9).

compute cogiat=1.

compute mdcogiat=nghiemtrong1.

else if (bc2a=9).

compute cogiat=1.

compute cogiat=nghiemtrong2.

else if (bc3a=9).

compute cogiat=1.

compute mdcogiat=nghiemtrong3.

else if (bckhaca=9).

compute cogiat=1.

compute mdcogiat=nghiemtrongkhac.

ELSE.

Compute cogiat=0.

compute mdcogiat=99.

end if.

Value labels mdcogiat

1 'tu vong' 2 'tinh mang' 3 'tan tat' 4 'keo dai nam vien' 5 'chi dinh nhap vien' 6 'tam dung hoac dung thuoc' 7 'doi thuoc' 8 'giam lieu thuoc lao' 9 'dung thuoc tri trieu chung' 10 'chay than' 11 'xu tri khac'

12 'khong nghiem trong' 14 'chua phan loai' 13 'khong xu tri'.

EXECUTE.

Do IF ($CASENUM=1).

compute cogiatkp=cogiat.

compute mdcogiatkp=mdcogiat.

compute tcogiat=tte.

ELSE IF (Table1x.ID = LAG(Table1x.ID)) AND cogiat=1 AND mdcogiat<lag(mdcogiatkp).

compute cogiatkp=cogiat.

compute mdcogiatkp=mdcogiat.

compute tcogiat=tte.

ELSE IF (Table1x.ID=lag(Table1x.ID) AND cogiat =1 AND mdcogiat>=lag(mdcogiatkp)).

COMPUTE cogiatkp=cogiat.

compute mdcogiatkp=lag(mdcogiatkp).

compute tcogiat=lag(tcogiat).

else if (Table1x.ID=lag(Table1x.ID) AND cogiat =0 AND lag(cogiatkp)=1).

compute cogiatkp=lag(cogiatkp).

compute mdcogiatkp=lag(mdcogiatkp).

compute tcogiat=lag(tcogiat).

ELSE.

compute cogiatkp=cogiat.

compute mdcogiatkp=mdcogiat.

compute tcogiat=tte.

END IF.

if ($CASENUM=1) and cogiatkp=1 tcogiata=tcogiat.

if (Table1x.ID<>lag(Table1x.ID)) and cogiatkp=1 tcogiata=tcogiat.

if (Table1x.ID=lag(Table1x.ID)) and cogiatkp=1 and lag(cogiatkp)=0 tcogiata=tcogiat.

if (Table1x.ID=lag(Table1x.ID)) and cogiatkp=1 and lag(cogiatkp)=1 tcogiata=lag(tcogiata).

if tcogiata>0 tcogiat=tcogiata.

Value labels mdcogiatkp

1 'tu vong' 2 'tinh mang' 3 'tan tat' 4 'keo dai nam vien' 5 'chi dinh nhap vien' 6 'tam dung hoac dung thuoc' 7 'doi thuoc' 8 'giam lieu thuoc lao' 9 'dung thuoc tri trieu chung' 10 'chay than' 11 'xu tri khac'

12 'khong nghiem trong' 14 'chua phan loai' 13 'khong xu tri'.

EXECUTE.

*** ROI LOAN TAM THAN***

do if (bc1a=10).

compute tamthan=1.

compute mdtamthan=nghiemtrong1.

else if (bc2a=10).

compute tamthan=1.

compute mdtamthan=nghiemtrong2.

else if (bc3a=10).

compute tamthan=1.

compute mdtamthan=nghiemtrong3.

else if (bckhaca=10).

compute tamthan=1.

compute mdtamthan=nghiemtrongkhac.

ELSE.

Compute tamthan=0.

compute mdtamthan=99.

end if.

Value labels mdtamthan

1 'tu vong' 2 'tinh mang' 3 'tan tat' 4 'keo dai nam vien' 5 'chi dinh nhap vien' 6 'tam dung hoac dung thuoc' 7 'doi thuoc' 8 'giam lieu thuoc lao' 9 'dung thuoc tri trieu chung' 10 'chay than' 11 'xu tri khac'

12 'khong nghiem trong' 14 'chua phan loai' 13 'khong xu tri'.

EXECUTE.

Do IF ($CASENUM=1).

compute tamthankp=tamthan.

compute mdtamthankp=mdtamthan.

compute ttamthan=tte.

ELSE IF (Table1x.ID = LAG(Table1x.ID)) AND tamthan=1 AND mdtamthan<lag(mdtamthankp).

compute tamthankp=tamthan.

compute mdtamthankp=mdtamthan.

compute ttamthan=tte.

ELSE IF (Table1x.ID=lag(Table1x.ID) AND tamthan =1 AND mdtamthan>=lag(mdtamthankp)).

COMPUTE tamthankp=tamthan.

compute mdtamthankp=lag(mdtamthankp).

compute ttamthan=lag(ttamthan).

else if (Table1x.ID=lag(Table1x.ID) AND tamthan =0 AND lag(tamthankp)=1).

compute tamthankp=lag(tamthankp).

compute mdtamthankp=lag(mdtamthankp).

compute ttamthan=lag(ttamthan).

ELSE.

compute tamthankp=tamthan.

compute mdtamthankp=mdtamthan.

compute ttamthan=tte.

END IF.

if ($CASENUM=1) and tamthankp=1 ttamthana=ttamthan.

if (Table1x.ID<>lag(Table1x.ID)) and tamthankp=1 ttamthana=ttamthan.

if (Table1x.ID=lag(Table1x.ID)) and tamthankp=1 and lag(tamthankp)=0 ttamthana=ttamthan.

if (Table1x.ID=lag(Table1x.ID)) and tamthankp=1 and lag(tamthankp)=1 ttamthana=lag(ttamthana).

if ttamthana>0 ttamthan=ttamthana.

Value labels mdtamthankp

1 'tu vong' 2 'tinh mang' 3 'tan tat' 4 'keo dai nam vien' 5 'chi dinh nhap vien' 6 'tam dung hoac dung thuoc' 7 'doi thuoc' 8 'giam lieu thuoc lao' 9 'dung thuoc tri trieu chung' 10 'chay than' 11 'xu tri khac'

12 'khong nghiem trong' 14 'chua phan loai' 13 'khong xu tri'.

EXECUTE.

***THAN KINH NGOAI VI***

do if (bc1a=11).

compute tkngoaivi=1.

compute mdtkngoaivi=nghiemtrong1.

else if (bc2a=11).

compute tkngoaivi=1.

compute mdtkngoaivi=nghiemtrong2.

else if (bc3a=11).

compute tkngoaivi=1.

compute mdtkngoaivi=nghiemtrong3.

else if (bckhaca=11).

compute tkngoaivi=1.

compute mdtkngoaivi=nghiemtrongkhac.

ELSE.

Compute tkngoaivi=0.

compute mdtkngoaivi=99.

end if.

Value labels mdtkngoaivi

1 'tu vong' 2 'tinh mang' 3 'tan tat' 4 'keo dai nam vien' 5 'chi dinh nhap vien' 6 'tam dung hoac dung thuoc' 7 'doi thuoc' 8 'giam lieu thuoc lao' 9 'dung thuoc tri trieu chung' 10 'chay than' 11 'xu tri khac'

12 'khong nghiem trong' 14 'chua phan loai' 13 'khong xu tri'.

EXECUTE.

Do IF ($CASENUM=1).

compute tkngoaivikp=tkngoaivi.

compute mdtkngoaivikp=mdtkngoaivi.

compute ttkngoaivi=tte.

ELSE IF (Table1x.ID = LAG(Table1x.ID)) AND tkngoaivi=1 AND mdtkngoaivi<lag(mdtkngoaivikp).

compute tkngoaivikp=tkngoaivi.

compute mdtkngoaivikp=mdtkngoaivi.

compute ttkngoaivi=tte.

ELSE IF (Table1x.ID=lag(Table1x.ID) AND tkngoaivi =1 AND mdtkngoaivi>=lag(mdtkngoaivikp)).

COMPUTE tkngoaivikp=tkngoaivi.

compute mdtkngoaivikp=lag(mdtkngoaivikp).

compute ttkngoaivi=lag(ttkngoaivi).

else if (Table1x.ID=lag(Table1x.ID) AND tkngoaivi =0 AND lag(tkngoaivikp)=1).

compute tkngoaivikp=lag(tkngoaivikp).

compute mdtkngoaivikp=lag(mdtkngoaivikp).

compute ttkngoaivi=lag(ttkngoaivi).

ELSE.

compute tkngoaivikp=tkngoaivi.

compute mdtkngoaivikp=mdtkngoaivi.

compute ttkngoaivi=tte.

END IF.

if ($CASENUM=1) and tkngoaivikp=1 ttkngoaivia=ttkngoaivi.

if (Table1x.ID<>lag(Table1x.ID)) and tkngoaivikp=1 ttkngoaivia=ttkngoaivi.

if (Table1x.ID=lag(Table1x.ID)) and tkngoaivikp=1 and lag(tkngoaivikp)=0 ttkngoaivia=ttkngoaivi.

if (Table1x.ID=lag(Table1x.ID)) and tkngoaivikp=1 and lag(tkngoaivikp)=1 ttkngoaivia=lag(ttkngoaivia).

if ttkngoaivia>0 ttkngoaivi=ttkngoaivia.

Value labels mdtkngoaivikp

1 'tu vong' 2 'tinh mang' 3 'tan tat' 4 'keo dai nam vien' 5 'chi dinh nhap vien' 6 'tam dung hoac dung thuoc' 7 'doi thuoc' 8 'giam lieu thuoc lao' 9 'dung thuoc tri trieu chung' 10 'chay than' 11 'xu tri khac'

12 'khong nghiem trong' 14 'chua phan loai' 13 'khong xu tri'.

EXECUTE.

***DAU KHOP***

do if (bc1a=12).

compute daukhop=1.

compute mddaukhop=nghiemtrong1.

else if (bc2a=12).

compute daukhop=1.

compute mddaukhop=nghiemtrong2.

else if (bc3a=12).

compute daukhop=1.

compute mddaukhop=nghiemtrong3.

else if (bckhaca=12).

compute daukhop=1.

compute mddaukhop=nghiemtrongkhac.

ELSE.

Compute daukhop=0.

compute mddaukhop=99.

end if.

Value labels mddaukhop

1 'tu vong' 2 'tinh mang' 3 'tan tat' 4 'keo dai nam vien' 5 'chi dinh nhap vien' 6 'tam dung hoac dung thuoc' 7 'doi thuoc' 8 'giam lieu thuoc lao' 9 'dung thuoc tri trieu chung' 10 'chay than' 11 'xu tri khac'

12 'khong nghiem trong' 14 'chua phan loai' 13 'khong xu tri'.

EXECUTE.

Do IF ($CASENUM=1).

compute daukhopkp=daukhop.

compute mddaukhopkp=mddaukhop.

compute tdaukhop=tte.

ELSE IF (Table1x.ID = LAG(Table1x.ID)) AND daukhop=1 AND mddaukhop<lag(mddaukhopkp).

compute daukhopkp=daukhop.

compute mddaukhopkp=mddaukhop.

compute tdaukhop=tte.

ELSE IF (Table1x.ID=lag(Table1x.ID) AND daukhop =1 AND mddaukhop>=lag(mddaukhopkp)).

COMPUTE daukhopkp=daukhop.

compute mddaukhopkp=lag(mddaukhopkp).

compute tdaukhop=lag(tdaukhop).

else if (Table1x.ID=lag(Table1x.ID) AND daukhop =0 AND lag(daukhopkp)=1).

compute daukhopkp=lag(daukhopkp).

compute mddaukhopkp=lag(mddaukhopkp).

compute tdaukhop=lag(tdaukhop).

ELSE.

compute daukhopkp=daukhop.

compute mddaukhopkp=mddaukhop.

compute tdaukhop=tte.

END IF.

if ($CASENUM=1) and daukhopkp=1 tdaukhopa=tdaukhop.

if (Table1x.ID<>lag(Table1x.ID)) and daukhopkp=1 tdaukhopa=tdaukhop.

if (Table1x.ID=lag(Table1x.ID)) and daukhopkp=1 and lag(daukhopkp)=0 tdaukhopa=tdaukhop.

if (Table1x.ID=lag(Table1x.ID)) and daukhopkp=1 and lag(daukhopkp)=1 tdaukhopa=lag(tdaukhopa).

if tdaukhopa>0 tdaukhop=tdaukhopa.

Value labels mddaukhopkp

1 'tu vong' 2 'tinh mang' 3 'tan tat' 4 'keo dai nam vien' 5 'chi dinh nhap vien' 6 'tam dung hoac dung thuoc' 7 'doi thuoc' 8 'giam lieu thuoc lao' 9 'dung thuoc tri trieu chung' 10 'chay than' 11 'xu tri khac'

12 'khong nghiem trong' 14 'chua phan loai' 13 'khong xu tri'.

EXECUTE.

***QUA MAN***

do if (bc1a=13).

compute quaman=1.

compute mdquaman=nghiemtrong1.

else if (bc2a=13).

compute quaman=1.

compute mdquaman=nghiemtrong2.

else if (bc3a=13).

compute quaman=1.

compute mdquaman=nghiemtrong3.

else if (bckhaca=13).

compute quaman=1.

compute mdquaman=nghiemtrongkhac.

ELSE.

Compute quaman=0.

compute mdquaman=99.

end if.

Value labels mdquaman

1 'tu vong' 2 'tinh mang' 3 'tan tat' 4 'keo dai nam vien' 5 'chi dinh nhap vien' 6 'tam dung hoac dung thuoc' 7 'doi thuoc' 8 'giam lieu thuoc lao' 9 'dung thuoc tri trieu chung' 10 'chay than' 11 'xu tri khac'

12 'khong nghiem trong' 14 'chua phan loai' 13 'khong xu tri'.

EXECUTE.

Do IF ($CASENUM=1).

compute quamankp=quaman.

compute mdquamankp=mdquaman.

compute tquaman=tte.

ELSE IF (Table1x.ID = LAG(Table1x.ID)) AND quaman=1 AND mdquaman<lag(mdquamankp).

compute quamankp=quaman.

compute mdquamankp=mdquaman.

compute tquaman=tte.

ELSE IF (Table1x.ID=lag(Table1x.ID) AND quaman =1 AND mdquaman>=lag(mdquamankp)).

COMPUTE quamankp=quaman.

compute mdquamankp=lag(mdquamankp).

compute tquaman=lag(tquaman).

else if (Table1x.ID=lag(Table1x.ID) AND quaman =0 AND lag(quamankp)=1).

compute quamankp=lag(quamankp).

compute mdquamankp=lag(mdquamankp).

compute tquaman=lag(tquaman).

ELSE.

compute quamankp=quaman.

compute mdquamankp=mdquaman.

compute tquaman=tte.

END IF.

if ($CASENUM=1) and quamankp=1 tquamana=tquaman.

if (Table1x.ID<>lag(Table1x.ID)) and quamankp=1 tquamana=tquaman.

if (Table1x.ID=lag(Table1x.ID)) and quamankp=1 and lag(quamankp)=0 tquamana=tquaman.

if (Table1x.ID=lag(Table1x.ID)) and quamankp=1 and lag(quamankp)=1 tquamana=lag(tquamana).

if tquamana>0 tquaman=tquamana.

Value labels mdquamankp

1 'tu vong' 2 'tinh mang' 3 'tan tat' 4 'keo dai nam vien' 5 'chi dinh nhap vien' 6 'tam dung hoac dung thuoc' 7 'doi thuoc' 8 'giam lieu thuoc lao' 9 'dung thuoc tri trieu chung' 10 'chay than' 11 'xu tri khac'

12 'khong nghiem trong' 14 'chua phan loai' 13 'khong xu tri'.

EXECUTE.

***DOC THAN***

do if (bc1a=14).

compute docthan=1.

compute mddocthan=nghiemtrong1.

else if (bc2a=14).

compute docthan=1.

compute mddocthan=nghiemtrong2.

else if (bc3a=14).

compute docthan=1.

compute mddocthan=nghiemtrong3.

else if (bckhaca=14).

compute docthan=1.

compute mddocthan=nghiemtrongkhac.

ELSE.

Compute docthan=0.

compute mddocthan=99.

end if.

Value labels mddocthan

1 'tu vong' 2 'tinh mang' 3 'tan tat' 4 'keo dai nam vien' 5 'chi dinh nhap vien' 6 'tam dung hoac dung thuoc' 7 'doi thuoc' 8 'giam lieu thuoc lao' 9 'dung thuoc tri trieu chung' 10 'chay than' 11 'xu tri khac'

12 'khong nghiem trong' 14 'chua phan loai' 13 'khong xu tri'.

EXECUTE.

Do IF ($CASENUM=1).

compute docthankp=docthan.

compute mddocthankp=mddocthan.

compute tdocthan=tte.

ELSE IF (Table1x.ID = LAG(Table1x.ID)) AND docthan=1 AND mddocthan<lag(mddocthankp).

compute docthankp=docthan.

compute mddocthankp=mddocthan.

compute tdocthan=tte.

ELSE IF (Table1x.ID=lag(Table1x.ID) AND docthan =1 AND mddocthan>=lag(mddocthankp)).

COMPUTE docthankp=docthan.

compute mddocthankp=lag(mddocthankp).

compute tdocthan=lag(tdocthan).

else if (Table1x.ID=lag(Table1x.ID) AND docthan =0 AND lag(docthankp)=1).

compute docthankp=lag(docthankp).

compute mddocthankp=lag(mddocthankp).

compute tdocthan=lag(tdocthan).

ELSE.

compute docthankp=docthan.

compute mddocthankp=mddocthan.

compute tdocthan=tte.

END IF.

if ($CASENUM=1) and docthankp=1 tdocthana=tdocthan.

if (Table1x.ID<>lag(Table1x.ID)) and docthankp=1 tdocthana=tdocthan.

if (Table1x.ID=lag(Table1x.ID)) and docthankp=1 and lag(docthankp)=0 tdocthana=tdocthan.

if (Table1x.ID=lag(Table1x.ID)) and docthankp=1 and lag(docthankp)=1 tdocthana=lag(tdocthana).

if tdocthana>0 tdocthan=tdocthana.

Value labels mddocthankp

1 'tu vong' 2 'tinh mang' 3 'tan tat' 4 'keo dai nam vien' 5 'chi dinh nhap vien' 6 'tam dung hoac dung thuoc' 7 'doi thuoc' 8 'giam lieu thuoc lao' 9 'dung thuoc tri trieu chung' 10 'chay than' 11 'xu tri khac'

12 'khong nghiem trong' 14 'chua phan loai' 13 'khong xu tri'.

EXECUTE.

***RL TIEN DINH***

do if (bc1a=15).

compute tiendinh=1.

compute mdtiendinh=nghiemtrong1.

else if (bc2a=15).

compute tiendinh=1.

compute mdtiendinh=nghiemtrong2.

else if (bc3a=15).

compute tiendinh=1.

compute mdtiendinh=nghiemtrong3.

else if (bckhaca=15).

compute tiendinh=1.

compute mdtiendinh=nghiemtrongkhac.

ELSE.

Compute tiendinh=0.

compute mdtiendinh=99.

end if.

Value labels mdtiendinh

1 'tu vong' 2 'tinh mang' 3 'tan tat' 4 'keo dai nam vien' 5 'chi dinh nhap vien' 6 'tam dung hoac dung thuoc' 7 'doi thuoc' 8 'giam lieu thuoc lao' 9 'dung thuoc tri trieu chung' 10 'chay than' 11 'xu tri khac'

12 'khong nghiem trong' 14 'chua phan loai' 13 'khong xu tri'.

EXECUTE.

Do IF ($CASENUM=1).

compute tiendinhkp=tiendinh.

compute mdtiendinhkp=mdtiendinh.

compute ttiendinh=tte.

ELSE IF (Table1x.ID = LAG(Table1x.ID)) AND tiendinh=1 AND mdtiendinh<lag(mdtiendinhkp).

compute tiendinhkp=tiendinh.

compute mdtiendinhkp=mdtiendinh.

compute ttiendinh=tte.

ELSE IF (Table1x.ID=lag(Table1x.ID) AND tiendinh =1 AND mdtiendinh>=lag(mdtiendinhkp)).

COMPUTE tiendinhkp=tiendinh.

compute mdtiendinhkp=lag(mdtiendinhkp).

compute ttiendinh=lag(ttiendinh).

else if (Table1x.ID=lag(Table1x.ID) AND tiendinh =0 AND lag(tiendinhkp)=1).

compute tiendinhkp=lag(tiendinhkp).

compute mdtiendinhkp=lag(mdtiendinhkp).

compute ttiendinh=lag(ttiendinh).

ELSE.

compute tiendinhkp=tiendinh.

compute mdtiendinhkp=mdtiendinh.

compute ttiendinh=tte.

END IF.

if ($CASENUM=1) and tiendinhkp=1 ttiendinha=ttiendinh.

if (Table1x.ID<>lag(Table1x.ID)) and tiendinhkp=1 ttiendinha=ttiendinh.

if (Table1x.ID=lag(Table1x.ID)) and tiendinhkp=1 and lag(tiendinhkp)=0 ttiendinha=ttiendinh.

if (Table1x.ID=lag(Table1x.ID)) and tiendinhkp=1 and lag(tiendinhkp)=1 ttiendinha=lag(ttiendinha).

if ttiendinha>0 ttiendinh=ttiendinha.

Value labels mdtiendinhkp

1 'tu vong' 2 'tinh mang' 3 'tan tat' 4 'keo dai nam vien' 5 'chi dinh nhap vien' 6 'tam dung hoac dung thuoc' 7 'doi thuoc' 8 'giam lieu thuoc lao' 9 'dung thuoc tri trieu chung' 10 'chay than' 11 'xu tri khac'

12 'khong nghiem trong' 14 'chua phan loai' 13 'khong xu tri'.

EXECUTE.

***RL THI GIAC***

do if (bc1a=16).

compute thigiac=1.

compute mdthigiac=nghiemtrong1.

else if (bc2a=16).

compute thigiac=1.

compute mdthigiac=nghiemtrong2.

else if (bc3a=16).

compute thigiac=1.

compute mdthigiac=nghiemtrong3.

else if (bckhaca=16).

compute thigiac=1.

compute mdthigiac=nghiemtrongkhac.

ELSE.

Compute thigiac=0.

compute mdthigiac=99.

end if.

Value labels mdthigiac

1 'tu vong' 2 'tinh mang' 3 'tan tat' 4 'keo dai nam vien' 5 'chi dinh nhap vien' 6 'tam dung hoac dung thuoc' 7 'doi thuoc' 8 'giam lieu thuoc lao' 9 'dung thuoc tri trieu chung' 10 'chay than' 11 'xu tri khac'

12 'khong nghiem trong' 14 'chua phan loai' 13 'khong xu tri'.

EXECUTE.

Do IF ($CASENUM=1).

compute thigiackp=thigiac.

compute mdthigiackp=mdthigiac.

compute tthigiac=tte.

ELSE IF (Table1x.ID = LAG(Table1x.ID)) AND thigiac=1 AND mdthigiac<lag(mdthigiackp).

compute thigiackp=thigiac.

compute mdthigiackp=mdthigiac.

compute tthigiac=tte.

ELSE IF (Table1x.ID=lag(Table1x.ID) AND thigiac =1 AND mdthigiac>=lag(mdthigiackp)).

COMPUTE thigiackp=thigiac.

compute mdthigiackp=lag(mdthigiackp).

compute tthigiac=lag(tthigiac).

else if (Table1x.ID=lag(Table1x.ID) AND thigiac =0 AND lag(thigiackp)=1).

compute thigiackp=lag(thigiackp).

compute mdthigiackp=lag(mdthigiackp).

compute tthigiac=lag(tthigiac).

ELSE.

compute thigiackp=thigiac.

compute mdthigiackp=mdthigiac.

compute tthigiac=tte.

END IF.

if ($CASENUM=1) and thigiackp=1 tthigiaca=tthigiac.

if (Table1x.ID<>lag(Table1x.ID)) and thigiackp=1 tthigiaca=tthigiac.

if (Table1x.ID=lag(Table1x.ID)) and thigiackp=1 and lag(thigiackp)=0 tthigiaca=tthigiac.

if (Table1x.ID=lag(Table1x.ID)) and thigiackp=1 and lag(thigiackp)=1 tthigiaca=lag(tthigiaca).

if tthigiaca>0 tthigiac=tthigiaca.

Value labels mdthigiackp

1 'tu vong' 2 'tinh mang' 3 'tan tat' 4 'keo dai nam vien' 5 'chi dinh nhap vien' 6 'tam dung hoac dung thuoc' 7 'doi thuoc' 8 'giam lieu thuoc lao' 9 'dung thuoc tri trieu chung' 10 'chay than' 11 'xu tri khac'

12 'khong nghiem trong' 14 'chua phan loai' 13 'khong xu tri'.

EXECUTE.

***SUY GIAP***

do if (bc1a=17).

compute suygiap=1.

compute mdsuygiap=nghiemtrong1.

else if (bc2a=17).

compute suygiap=1.

compute mdsuygiap=nghiemtrong2.

else if (bc3a=17).

compute suygiap=1.

compute mdsuygiap=nghiemtrong3.

else if (bckhaca=17).

compute suygiap=1.

compute mdsuygiap=nghiemtrongkhac.

ELSE.

Compute suygiap=0.

compute mdsuygiap=99.

end if.

Value labels mdsuygiap

1 'tu vong' 2 'tinh mang' 3 'tan tat' 4 'keo dai nam vien' 5 'chi dinh nhap vien' 6 'tam dung hoac dung thuoc' 7 'doi thuoc' 8 'giam lieu thuoc lao' 9 'dung thuoc tri trieu chung' 10 'chay than' 11 'xu tri khac'

12 'khong nghiem trong' 14 'chua phan loai' 13 'khong xu tri'.

EXECUTE.

Do IF ($CASENUM=1).

compute suygiapkp=suygiap.

compute mdsuygiapkp=mdsuygiap.

compute tsuygiap=tte.

ELSE IF (Table1x.ID = LAG(Table1x.ID)) AND suygiap=1 AND mdsuygiap<lag(mdsuygiapkp).

compute suygiapkp=suygiap.

compute mdsuygiapkp=mdsuygiap.

compute tsuygiap=tte.

ELSE IF (Table1x.ID=lag(Table1x.ID) AND suygiap =1 AND mdsuygiap>=lag(mdsuygiapkp)).

COMPUTE suygiapkp=suygiap.

compute mdsuygiapkp=lag(mdsuygiapkp).

compute tsuygiap=lag(tsuygiap).

else if (Table1x.ID=lag(Table1x.ID) AND suygiap =0 AND lag(suygiapkp)=1).

compute suygiapkp=lag(suygiapkp).

compute mdsuygiapkp=lag(mdsuygiapkp).

compute tsuygiap=lag(tsuygiap).

ELSE.

compute suygiapkp=suygiap.

compute mdsuygiapkp=mdsuygiap.

compute tsuygiap=tte.

END IF.

if ($CASENUM=1) and suygiapkp=1 tsuygiapa=tsuygiap.

if (Table1x.ID<>lag(Table1x.ID)) and suygiapkp=1 tsuygiapa=tsuygiap.

if (Table1x.ID=lag(Table1x.ID)) and suygiapkp=1 and lag(suygiapkp)=0 tsuygiapa=tsuygiap.

if (Table1x.ID=lag(Table1x.ID)) and suygiapkp=1 and lag(suygiapkp)=1 tsuygiapa=lag(tsuygiapa).

if tsuygiapa>0 tsuygiap=tsuygiapa.

Value labels mdsuygiapkp

1 'tu vong' 2 'tinh mang' 3 'tan tat' 4 'keo dai nam vien' 5 'chi dinh nhap vien' 6 'tam dung hoac dung thuoc' 7 'doi thuoc' 8 'giam lieu thuoc lao' 9 'dung thuoc tri trieu chung' 10 'chay than' 11 'xu tri khac'

12 'khong nghiem trong' 14 'chua phan loai' 13 'khong xu tri'.

EXECUTE.

***HA KALI MAU***

do if (bc1a=18).

compute hakali=1.

compute mdhakali=nghiemtrong1.

else if (bc2a=18).

compute hakali=1.

compute mdhakali=nghiemtrong2.

else if (bc3a=18).

compute hakali=1.

compute mdhakali=nghiemtrong3.

else if (bckhaca=18).

compute hakali=1.

compute mdhakali=nghiemtrongkhac.

ELSE.

Compute hakali=0.

compute mdhakali=99.

end if.

Value labels mdhakali

1 'tu vong' 2 'tinh mang' 3 'tan tat' 4 'keo dai nam vien' 5 'chi dinh nhap vien' 6 'tam dung hoac dung thuoc' 7 'doi thuoc' 8 'giam lieu thuoc lao' 9 'dung thuoc tri trieu chung' 10 'chay than' 11 'xu tri khac'

12 'khong nghiem trong' 14 'chua phan loai' 13 'khong xu tri'.

EXECUTE.

Do IF ($CASENUM=1).

compute hakalikp=hakali.

compute mdhakalikp=mdhakali.

compute thakali=tte.

ELSE IF (Table1x.ID = LAG(Table1x.ID)) AND hakali=1 AND mdhakali<lag(mdhakalikp).

compute hakalikp=hakali.

compute mdhakalikp=mdhakali.

compute thakali=tte.

ELSE IF (Table1x.ID=lag(Table1x.ID) AND hakali =1 AND mdhakali>=lag(mdhakalikp)).

COMPUTE hakalikp=hakali.

compute mdhakalikp=lag(mdhakalikp).

compute thakali=lag(thakali).

else if (Table1x.ID=lag(Table1x.ID) AND hakali =0 AND lag(hakalikp)=1).

compute hakalikp=lag(hakalikp).

compute mdhakalikp=lag(mdhakalikp).

compute thakali=lag(thakali).

ELSE.

compute hakalikp=hakali.

compute mdhakalikp=mdhakali.

compute thakali=tte.

END IF.

if ($CASENUM=1) and hakalikp=1 thakalia=thakali.

if (Table1x.ID<>lag(Table1x.ID)) and hakalikp=1 thakalia=thakali.

if (Table1x.ID=lag(Table1x.ID)) and hakalikp=1 and lag(hakalikp)=0 thakalia=thakali.

if (Table1x.ID=lag(Table1x.ID)) and hakalikp=1 and lag(hakalikp)=1 thakalia=lag(thakalia).

if thakalia>0 thakali=thakalia.

Value labels mdhakalikp

1 'tu vong' 2 'tinh mang' 3 'tan tat' 4 'keo dai nam vien' 5 'chi dinh nhap vien' 6 'tam dung hoac dung thuoc' 7 'doi thuoc' 8 'giam lieu thuoc lao' 9 'dung thuoc tri trieu chung' 10 'chay than' 11 'xu tri khac'

12 'khong nghiem trong' 14 'chua phan loai' 13 'khong xu tri'.

EXECUTE.

***TANG ACID URIC***

do if (bc1a=19).

compute tanguric=1.

compute mdtanguric=nghiemtrong1.

else if (bc2a=19).

compute tanguric=1.

compute mdtanguric=nghiemtrong2.

else if (bc3a=19).

compute tanguric=1.

compute mdtanguric=nghiemtrong3.

else if (bckhaca=19).

compute tanguric=1.

compute mdtanguric=nghiemtrongkhac.

ELSE.

Compute tanguric=0.

compute mdtanguric=99.

end if.

Value labels mdtanguric

1 'tu vong' 2 'tinh mang' 3 'tan tat' 4 'keo dai nam vien' 5 'chi dinh nhap vien' 6 'tam dung hoac dung thuoc' 7 'doi thuoc' 8 'giam lieu thuoc lao' 9 'dung thuoc tri trieu chung' 10 'chay than' 11 'xu tri khac'

12 'khong nghiem trong' 14 'chua phan loai' 13 'khong xu tri'.

EXECUTE.

Do IF ($CASENUM=1).

compute tangurickp=tanguric.

compute mdtangurickp=mdtanguric.

compute ttanguric=tte.

ELSE IF (Table1x.ID = LAG(Table1x.ID)) AND tanguric=1 AND mdtanguric<lag(mdtangurickp).

compute tangurickp=tanguric.

compute mdtangurickp=mdtanguric.

compute ttanguric=tte.

ELSE IF (Table1x.ID=lag(Table1x.ID) AND tanguric =1 AND mdtanguric>=lag(mdtangurickp)).

COMPUTE tangurickp=tanguric.

compute mdtangurickp=lag(mdtangurickp).

compute ttanguric=lag(ttanguric).

else if (Table1x.ID=lag(Table1x.ID) AND tanguric =0 AND lag(tangurickp)=1).

compute tangurickp=lag(tangurickp).

compute mdtangurickp=lag(mdtangurickp).

compute ttanguric=lag(ttanguric).

ELSE.

compute tangurickp=tanguric.

compute mdtangurickp=mdtanguric.

compute ttanguric=tte.

END IF.

if ($CASENUM=1) and tangurickp=1 ttangurica=ttanguric.

if (Table1x.ID<>lag(Table1x.ID)) and tangurickp=1 ttangurica=ttanguric.

if (Table1x.ID=lag(Table1x.ID)) and tangurickp=1 and lag(tangurickp)=0 ttangurica=ttanguric.

if (Table1x.ID=lag(Table1x.ID)) and tangurickp=1 and lag(tangurickp)=1 ttangurica=lag(ttangurica).

if ttangurica>0 ttanguric=ttangurica.

Value labels mdtangurickp

1 'tu vong' 2 'tinh mang' 3 'tan tat' 4 'keo dai nam vien' 5 'chi dinh nhap vien' 6 'tam dung hoac dung thuoc' 7 'doi thuoc' 8 'giam lieu thuoc lao' 9 'dung thuoc tri trieu chung' 10 'chay than' 11 'xu tri khac'

12 'khong nghiem trong' 14 'chua phan loai' 13 'khong xu tri'.

EXECUTE.

***ROI LOAN HUYET HOC

do if (bc1a=20).

compute huyethoc=1.

compute mdhuyethoc=nghiemtrong1.

else if (bc2a=20).

compute huyethoc=1.

compute mdhuyethoc=nghiemtrong2.

else if (bc3a=20).

compute huyethoc=1.

compute mdhuyethoc=nghiemtrong3.

else if (bckhaca=20).

compute huyethoc=1.

compute mdhuyethoc=nghiemtrongkhac.

ELSE.

Compute huyethoc=0.

compute mdhuyethoc=99.

end if.

Value labels mdhuyethoc

1 'tu vong' 2 'tinh mang' 3 'tan tat' 4 'keo dai nam vien' 5 'chi dinh nhap vien' 6 'tam dung hoac dung thuoc' 7 'doi thuoc' 8 'giam lieu thuoc lao' 9 'dung thuoc tri trieu chung' 10 'chay than' 11 'xu tri khac'

12 'khong nghiem trong' 14 'chua phan loai' 13 'khong xu tri'.

EXECUTE.

Do IF ($CASENUM=1).

compute huyethockp=huyethoc.

compute mdhuyethockp=mdhuyethoc.

compute thuyethoc=tte.

ELSE IF (Table1x.ID = LAG(Table1x.ID)) AND huyethoc=1 AND mdhuyethoc<lag(mdhuyethockp).

compute huyethockp=huyethoc.

compute mdhuyethockp=mdhuyethoc.

compute thuyethoc=tte.

ELSE IF (Table1x.ID=lag(Table1x.ID) AND huyethoc =1 AND mdhuyethoc>=lag(mdhuyethockp)).

COMPUTE huyethockp=huyethoc.

compute mdhuyethockp=lag(mdhuyethockp).

compute thuyethoc=lag(thuyethoc).

else if (Table1x.ID=lag(Table1x.ID) AND huyethoc =0 AND lag(huyethockp)=1).

compute huyethockp=lag(huyethockp).

compute mdhuyethockp=lag(mdhuyethockp).

compute thuyethoc=lag(thuyethoc).

ELSE.

compute huyethockp=huyethoc.

compute mdhuyethockp=mdhuyethoc.

compute thuyethoc=tte.

END IF.

if ($CASENUM=1) and huyethockp=1 thuyethoca=thuyethoc.

if (Table1x.ID<>lag(Table1x.ID)) and huyethockp=1 thuyethoca=thuyethoc.

if (Table1x.ID=lag(Table1x.ID)) and huyethockp=1 and lag(huyethockp)=0 thuyethoca=thuyethoc.

if (Table1x.ID=lag(Table1x.ID)) and huyethockp=1 and lag(huyethockp)=1 thuyethoca=lag(thuyethoca).

if thuyethoca>0 thuyethoc=thuyethoca.

Value labels mdhuyethockp

1 'tu vong' 2 'tinh mang' 3 'tan tat' 4 'keo dai nam vien' 5 'chi dinh nhap vien' 6 'tam dung hoac dung thuoc' 7 'doi thuoc' 8 'giam lieu thuoc lao' 9 'dung thuoc tri trieu chung' 10 'chay than' 11 'xu tri khac'

12 'khong nghiem trong' 14 'chua phan loai' 13 'khong xu tri'.

EXECUTE.

***ROI LOAN NOI TIET***

do if (bc1a=21).

compute noitiet=1.

compute mdnoitiet=nghiemtrong1.

else if (bc2a=21).

compute noitiet=1.

compute mdnoitiet=nghiemtrong2.

else if (bc3a=21).

compute noitiet=1.

compute mdnoitiet=nghiemtrong3.

else if (bckhaca=21).

compute noitiet=1.

compute mdnoitiet=nghiemtrongkhac.

ELSE.

Compute noitiet=0.

compute mdnoitiet=99.

end if.

Value labels mdnoitiet

1 'tu vong' 2 'tinh mang' 3 'tan tat' 4 'keo dai nam vien' 5 'chi dinh nhap vien' 6 'tam dung hoac dung thuoc' 7 'doi thuoc' 8 'giam lieu thuoc lao' 9 'dung thuoc tri trieu chung' 10 'chay than' 11 'xu tri khac'

12 'khong nghiem trong' 14 'chua phan loai' 13 'khong xu tri'.

EXECUTE.

Do IF ($CASENUM=1).

compute noitietkp=noitiet.

compute mdnoitietkp=mdnoitiet.

compute tnoitiet=tte.

ELSE IF (Table1x.ID = LAG(Table1x.ID)) AND noitiet=1 AND mdnoitiet<lag(mdnoitietkp).

compute noitietkp=noitiet.

compute mdnoitietkp=mdnoitiet.

compute tnoitiet=tte.

ELSE IF (Table1x.ID=lag(Table1x.ID) AND noitiet =1 AND mdnoitiet>=lag(mdnoitietkp)).

COMPUTE noitietkp=noitiet.

compute mdnoitietkp=lag(mdnoitietkp).

compute tnoitiet=lag(tnoitiet).

else if (Table1x.ID=lag(Table1x.ID) AND noitiet =0 AND lag(noitietkp)=1).

compute noitietkp=lag(noitietkp).

compute mdnoitietkp=lag(mdnoitietkp).

compute tnoitiet=lag(tnoitiet).

ELSE.

compute noitietkp=noitiet.

compute mdnoitietkp=mdnoitiet.

compute tnoitiet=tte.

END IF.

if ($CASENUM=1) and noitietkp=1 tnoitieta=tnoitiet.

if (Table1x.ID<>lag(Table1x.ID)) and noitietkp=1 tnoitieta=tnoitiet.

if (Table1x.ID=lag(Table1x.ID)) and noitietkp=1 and lag(noitietkp)=0 tnoitieta=tnoitiet.

if (Table1x.ID=lag(Table1x.ID)) and noitietkp=1 and lag(noitietkp)=1 tnoitieta=lag(tnoitieta).

if tnoitieta>0 tnoitiet=tnoitieta.

Value labels mdnoitietkp

1 'tu vong' 2 'tinh mang' 3 'tan tat' 4 'keo dai nam vien' 5 'chi dinh nhap vien' 6 'tam dung hoac dung thuoc' 7 'doi thuoc' 8 'giam lieu thuoc lao' 9 'dung thuoc tri trieu chung' 10 'chay than' 11 'xu tri khac'

12 'khong nghiem trong' 14 'chua phan loai' 13 'khong xu tri'.

EXECUTE.

***PHU TAI CHO ***

do if (bc1a=22).

compute phu=1.

compute mdphu=nghiemtrong1.

else if (bc2a=22).

compute phu=1.

compute mdphu=nghiemtrong2.

else if (bc3a=22).

compute phu=1.

compute mdphu=nghiemtrong3.

else if (bckhaca=22).

compute phu=1.

compute mdphu=nghiemtrongkhac.

ELSE.

Compute phu=0.

compute mdphu=99.

end if.

Value labels mdphu

1 'tu vong' 2 'tinh mang' 3 'tan tat' 4 'keo dai nam vien' 5 'chi dinh nhap vien' 6 'tam dung hoac dung thuoc' 7 'doi thuoc' 8 'giam lieu thuoc lao' 9 'dung thuoc tri trieu chung' 10 'chay than' 11 'xu tri khac'

12 'khong nghiem trong' 14 'chua phan loai' 13 'khong xu tri'.

EXECUTE.

Do IF ($CASENUM=1).

compute phukp=phu.

compute mdphukp=mdphu.

compute tphu=tte.

ELSE IF (Table1x.ID = LAG(Table1x.ID)) AND phu=1 AND mdphu<lag(mdphukp).

compute phukp=phu.

compute mdphukp=mdphu.

compute tphu=tte.

ELSE IF (Table1x.ID=lag(Table1x.ID) AND phu =1 AND mdphu>=lag(mdphukp)).

COMPUTE phukp=phu.

compute mdphukp=lag(mdphukp).

compute tphu=lag(tphu).

else if (Table1x.ID=lag(Table1x.ID) AND phu =0 AND lag(phukp)=1).

compute phukp=lag(phukp).

compute mdphukp=lag(mdphukp).

compute tphu=lag(tphu).

ELSE.

compute phukp=phu.

compute mdphukp=mdphu.

compute tphu=tte.

END IF.

if ($CASENUM=1) and phukp=1 tphua=tphu.

if (Table1x.ID<>lag(Table1x.ID)) and phukp=1 tphua=tphu.

if (Table1x.ID=lag(Table1x.ID)) and phukp=1 and lag(phukp)=0 tphua=tphu.

if (Table1x.ID=lag(Table1x.ID)) and phukp=1 and lag(phukp)=1 tphua=lag(tphua).

if tphua>0 tphu=tphua.

Value labels mdphukp

1 'tu vong' 2 'tinh mang' 3 'tan tat' 4 'keo dai nam vien' 5 'chi dinh nhap vien' 6 'tam dung hoac dung thuoc' 7 'doi thuoc' 8 'giam lieu thuoc lao' 9 'dung thuoc tri trieu chung' 10 'chay than' 11 'xu tri khac'

12 'khong nghiem trong' 14 'chua phan loai' 13 'khong xu tri'.

EXECUTE.

***SHOCK***

do if (bc1a=23).

compute soc=1.

compute mdsoc=nghiemtrong1.

else if (bc2a=23).

compute soc=1.

compute mdsoc=nghiemtrong2.

else if (bc3a=23).

compute soc=1.

compute mdsoc=nghiemtrong3.

else if (bckhaca=23).

compute soc=1.

compute mdsoc=nghiemtrongkhac.

ELSE.

Compute soc=0.

compute mdsoc=99.

end if.

Value labels mdsoc

1 'tu vong' 2 'tinh mang' 3 'tan tat' 4 'keo dai nam vien' 5 'chi dinh nhap vien' 6 'tam dung hoac dung thuoc' 7 'doi thuoc' 8 'giam lieu thuoc lao' 9 'dung thuoc tri trieu chung' 10 'chay than' 11 'xu tri khac'

12 'khong nghiem trong' 14 'chua phan loai' 13 'khong xu tri'.

EXECUTE.

Do IF ($CASENUM=1).

compute sockp=soc.

compute mdsockp=mdsoc.

compute tsoc=tte.

ELSE IF (Table1x.ID = LAG(Table1x.ID)) AND soc=1 AND mdsoc<lag(mdsockp).

compute sockp=soc.

compute mdsockp=mdsoc.

compute tsoc=tte.

ELSE IF (Table1x.ID=lag(Table1x.ID) AND soc =1 AND mdsoc>=lag(mdsockp)).

COMPUTE sockp=soc.

compute mdsockp=lag(mdsockp).

compute tsoc=lag(tsoc).

else if (Table1x.ID=lag(Table1x.ID) AND soc =0 AND lag(sockp)=1).

compute sockp=lag(sockp).

compute mdsockp=lag(mdsockp).

compute tsoc=lag(tsoc).

ELSE.

compute sockp=soc.

compute mdsockp=mdsoc.

compute tsoc=tte.

END IF.

if ($CASENUM=1) and sockp=1 tsoca=tsoc.

if (Table1x.ID<>lag(Table1x.ID)) and sockp=1 tsoca=tsoc.

if (Table1x.ID=lag(Table1x.ID)) and sockp=1 and lag(sockp)=0 tsoca=tsoc.

if (Table1x.ID=lag(Table1x.ID)) and sockp=1 and lag(sockp)=1 tsoca=lag(tsoca).

if tsoca>0 tsoc=tsoca.

Value labels mdsockp

1 'tu vong' 2 'tinh mang' 3 'tan tat' 4 'keo dai nam vien' 5 'chi dinh nhap vien' 6 'tam dung hoac dung thuoc' 7 'doi thuoc' 8 'giam lieu thuoc lao' 9 'dung thuoc tri trieu chung' 10 'chay than' 11 'xu tri khac'

12 'khong nghiem trong' 14 'chua phan loai' 13 'khong xu tri'.

EXECUTE.

***MET MOI***

do if (bc1a=27).

compute met=1.

compute mdmet=nghiemtrong1.

else if (bc2a=27).

compute met=1.

compute mdmet=nghiemtrong2.

else if (bc3a=27).

compute met=1.

compute mdmet=nghiemtrong3.

else if (bckhaca=27).

compute met=1.

compute mdmet=nghiemtrongkhac.

ELSE.

Compute met=0.

compute mdmet=99.

end if.

Value labels mdmet

1 'tu vong' 2 'tinh mang' 3 'tan tat' 4 'keo dai nam vien' 5 'chi dinh nhap vien' 6 'tam dung hoac dung thuoc' 7 'doi thuoc' 8 'giam lieu thuoc lao' 9 'dung thuoc tri trieu chung' 10 'chay than' 11 'xu tri khac'

12 'khong nghiem trong' 14 'chua phan loai' 13 'khong xu tri'.

EXECUTE.

Do IF ($CASENUM=1).

compute metkp=met.

compute mdmetkp=mdmet.

compute tmet=tte.

ELSE IF (Table1x.ID = LAG(Table1x.ID)) AND met=1 AND mdmet<lag(mdmetkp).

compute metkp=met.

compute mdmetkp=mdmet.

compute tmet=tte.

ELSE IF (Table1x.ID=lag(Table1x.ID) AND met =1 AND mdmet>=lag(mdmetkp)).

COMPUTE metkp=met.

compute mdmetkp=lag(mdmetkp).

compute tmet=lag(tmet).

else if (Table1x.ID=lag(Table1x.ID) AND met =0 AND lag(metkp)=1).

compute metkp=lag(metkp).

compute mdmetkp=lag(mdmetkp).

compute tmet=lag(tmet).

ELSE.

compute metkp=met.

compute mdmetkp=mdmet.

compute tmet=tte.

END IF.

if ($CASENUM=1) and metkp=1 tmeta=tmet.

if (Table1x.ID<>lag(Table1x.ID)) and metkp=1 tmeta=tmet.

if (Table1x.ID=lag(Table1x.ID)) and metkp=1 and lag(metkp)=0 tmeta=tmet.

if (Table1x.ID=lag(Table1x.ID)) and metkp=1 and lag(metkp)=1 tmeta=lag(tmeta).

if tmeta>0 tmet=tmeta.

Value labels mdmetkp

1 'tu vong' 2 'tinh mang' 3 'tan tat' 4 'keo dai nam vien' 5 'chi dinh nhap vien' 6 'tam dung hoac dung thuoc' 7 'doi thuoc' 8 'giam lieu thuoc lao' 9 'dung thuoc tri trieu chung' 10 'chay than' 11 'xu tri khac'

12 'khong nghiem trong' 14 'chua phan loai' 13 'khong xu tri'.

EXECUTE.

***TANG DUONG HUYET***

do if (bc1a=32).

compute glucose=1.

compute mdglucose=nghiemtrong1.

else if (bc2a=32).

compute glucose=1.

compute mdglucose=nghiemtrong2.

else if (bc3a=32).

compute glucose=1.

compute mdglucose=nghiemtrong3.

else if (bckhaca=32).

compute glucose=1.

compute mdglucose=nghiemtrongkhac.

ELSE.

Compute glucose=0.

compute mdglucose=99.

end if.

Value labels mdglucose

1 'tu vong' 2 'tinh mang' 3 'tan tat' 4 'keo dai nam vien' 5 'chi dinh nhap vien' 6 'tam dung hoac dung thuoc' 7 'doi thuoc' 8 'giam lieu thuoc lao' 9 'dung thuoc tri trieu chung' 10 'chay than' 11 'xu tri khac'

12 'khong nghiem trong' 14 'chua phan loai' 13 'khong xu tri'.

EXECUTE.

Do IF ($CASENUM=1).

compute glucosekp=glucose.

compute mdglucosekp=mdglucose.

compute tglucose=tte.

ELSE IF (Table1x.ID = LAG(Table1x.ID)) AND glucose=1 AND mdglucose<lag(mdglucosekp).

compute glucosekp=glucose.

compute mdglucosekp=mdglucose.

compute tglucose=tte.

ELSE IF (Table1x.ID=lag(Table1x.ID) AND glucose =1 AND mdglucose>=lag(mdglucosekp)).

COMPUTE glucosekp=glucose.

compute mdglucosekp=lag(mdglucosekp).

compute tglucose=lag(tglucose).

else if (Table1x.ID=lag(Table1x.ID) AND glucose =0 AND lag(glucosekp)=1).

compute glucosekp=lag(glucosekp).

compute mdglucosekp=lag(mdglucosekp).

compute tglucose=lag(tglucose).

ELSE.

compute glucosekp=glucose.

compute mdglucosekp=mdglucose.

compute tglucose=tte.

END IF.

if ($CASENUM=1) and glucosekp=1 tglucosea=tglucose.

if (Table1x.ID<>lag(Table1x.ID)) and glucosekp=1 tglucosea=tglucose.

if (Table1x.ID=lag(Table1x.ID)) and glucosekp=1 and lag(glucosekp)=0 tglucosea=tglucose.

if (Table1x.ID=lag(Table1x.ID)) and glucosekp=1 and lag(glucosekp)=1 tglucosea=lag(tglucosea).

if tglucosea>0 tglucose=tglucosea.

Value labels mdglucosekp

1 'tu vong' 2 'tinh mang' 3 'tan tat' 4 'keo dai nam vien' 5 'chi dinh nhap vien' 6 'tam dung hoac dung thuoc' 7 'doi thuoc' 8 'giam lieu thuoc lao' 9 'dung thuoc tri trieu chung' 10 'chay than' 11 'xu tri khac'

12 'khong nghiem trong' 14 'chua phan loai' 13 'khong xu tri'.

EXECUTE.

***Doc gan***

Do if (docganxd=1 OR docgannn=1).

compute docgan=1.

ELSE.

Compute docgan=0.

end if.

Do IF ($CASENUM=1).

compute docgankp=docgan.

compute tdocgan=tte.

ELSE IF (Table1x.ID <> LAG(Table1x.ID)).

compute docgankp=docgan.

compute tdocgan=tte.

ELSE IF (Table1x.ID = LAG(Table1x.ID)).

compute docgankp=docgan.

compute tdocgan=tte.

end if.

recode docgankp (missing=0).

recode tdocgan (missing=0).

if (Table1x.ID=lag(Table1x.ID) AND lag(docgankp)=1 AND docgankp=0) docgankp=lag(docgankp).

if (Table1x.ID=lag(Table1x.ID) AND lag(docgankp)=1 AND docgankp=1) tdocgan=lag(tdocgan).

*Viemdd***

Do if (dadayxd=1 OR dadaynn=1).

compute viemdd=1.

ELSE.

Compute viemdd=0.

end if.

Do IF ($CASENUM=1).

compute viemddkp=viemdd.

compute tviemdd=tte.

ELSE IF (Table1x.ID <> LAG(Table1x.ID)).

compute viemddkp=viemdd.

compute tviemdd=tte.

ELSE IF (Table1x.ID = LAG(Table1x.ID)).

compute viemddkp=viemdd.

compute tviemdd=tte.

end if.

recode viemddkp (missing=0).

recode tviemdd (missing=0).

if (Table1x.ID=lag(Table1x.ID) AND lag(viemddkp)=1 AND viemddkp=0) viemddkp=lag(viemddkp).

if (Table1x.ID=lag(Table1x.ID) AND lag(viemddkp)=1 AND viemddkp=1) tviemdd=lag(tviemdd).

compute id=Table1x.ID.

*DOC GAN (3 lan gioi han tren+TCLS vaf treen 5 lan gioi han tren)

if docganxd=1 docgana=1.

recode motabc1 ('66;ASAT=256.6;ALAT=228.5'=1) ('66;ASAT=232;ALAT=157.3;6.7;TP=20;TD=6'=1) ('66;ASAT=229.7;ALAT=145.8'=1) ('66;ASAT=189.9;ALAT=101'=1) ('AST = 438, ALT = 227'=1) ('AST = 296, ALT = 119'=1) ('61,62,64,66,67 (AST 199.9; ALT 112.5; Bil tp 41.6; Bil tự do 17.9)'=1) ('AST = 187, ALT = 182'=1) ('Ast/ALT=124,8/195,6'=1) ('AST/ALT=352,4/290,9'=1) ('AST/ALT=190,2/133,9'=1) ('AST/Alt=247,1/201'=1) ('AST/ALT=173,9/174,6'=1) ('AST/ALT=279,3/176,5'=1) ('AST/ALT=243,2/259,9'=1) ('AST/ALT=178,4/101,3'=1) ('AST/ALT=358,4/227,7'=1) ('61;62;64;66;67 (asat 114.2;alat 112;Bil tp 50.7;Bil tự do 13.6)'=1) ('bilirubin tt=5,2/ bilirubin tp=18,6;GOT/GPt=338,3/384,4'=1) ('GOT/GPT=197,8/47,5; glucose =2,2/ K+=5,13'=1) ('GOT/GPT=263,9/68,8'=1) ('GPT=200,7'=1) ('GOT=213,3'=1) ('AST/ALT=76/222'=1) ('GOT/GPT=209,6/74,8/GGT=346,9/albumin=28,4'=1) ('66;ASAT=397;ALAT=285.4'=1) ('66;ASAt=493.9;ALAT=253.6'=1) ('ASAT=241,1; ALAT=112.8'=1)

('6.6;ASAT=176.7;ALAT=160.4'=1) ('66;ASAT=70.1;ALAT=69;6.7;TD=5.2'=1) into docgana.

recode motabc2 ('6.6;ASAT=143.1;ALAT=272.5;6.7;TP=19.2;TD=6.9'=1) ('AST = 165, ALT = 170'=1) into docgana.

if docganxd=1 or docgannn=1 or docgana=1 docganchung=1.

recode docgana (missing=0).

do if docgana=1.

compute kpdocgana=1.

ELSE .

compute kpdocgana=0.

end if.

if id=lag(id) and lag(kpdocgana)=1 kpdocgana=lag(kpdocgana).

Do if ((id=lag(id)) and (kpdocgana=0)).

compute tgdocgana=tte.

else if (id=lag(id) and kpdocgana=1 and lag(kpdocgana)=0).

compute tgdocgana=tte.

else if (id=lag(id) and kpdocgana=1 and lag(kpdocgana)=1).

compute tgdocgana=lag(tgdocgana).

ELSE.

compute tgdocgana=tte.

end if.

* Roi loan he tieu hoa

if nonkp=1 or dadaynnkp=1 or dadayxdkp=1 or daubungkp=1 or tieuchaykp=1 rloanthoakp=1.

recode rloanthoakp (missing=0).

if mdnon=5 or mddadaynn=5 or mddadayxd=5 or mddaubung=5 or mdtieuchay=5 mdrloanthoa=5.

if mdnon=4 or mddadaynn=4 or mddadayxd=4 or mddaubung=4 or mdtieuchay=4 mdrloanthoa=4.

if mdnon=3 or mddadaynn=3 or mddadayxd=3 or mddaubung=3 or mdtieuchay=3 mdrloanthoa=3.

if mdnon=2 or mddadaynn=2 or mddadayxd=2 or mddaubung=2 or mdtieuchay=2 mdrloanthoa=2.

if mdnon=1 or mddadaynn=1 or mddadayxd=1 or mddaubung=1 or mdtieuchay=1 mdrloanthoa=1.

Value labels mdrloanthoa

1 'tu vong' 2 'tinh mang' 3 'tan tat' 4 'keo dai nam vien' 5 'chi dinh nhap vien'.

EXECUTE.

AGGREGATE

/OUTFILE=* MODE=ADDVARIABLES

/BREAK=Table1x.ID

/mdrloanthoa_min=MIN(mdrloanthoa).

if mdrloanthoa_min>0 rloanthoa_sae_kp=rloanthoakp.

* Roi loan than kinh trung uong

if hoamatkp=1 or daudaukp=1 or cogiatkp=1 rltktrunguongkp=1.

recode rltktrunguongkp (missing=0).

if mdhoamat=5 or mddaudau=5 or mdcogiat=5 mdrltktrunguong=5.

if mdhoamat=4 or mddaudau=4 or mdcogiat=4 mdrltktrunguong=4.

if mdhoamat=3 or mddaudau=3 or mdcogiat=3 mdrltktrunguong=3.

if mdhoamat=2 or mddaudau=2 or mdcogiat=2 mdrltktrunguong=2.

if mdhoamat=1 or mddaudau=1 or mdcogiat=1 mdrltktrunguong=1.

Value labels mdrltktrunguong

1 'tu vong' 2 'tinh mang' 3 'tan tat' 4 'keo dai nam vien' 5 'chi dinh nhap vien'.

EXECUTE.

AGGREGATE

/OUTFILE=* MODE=ADDVARIABLES

/BREAK=Table1x.ID

/mdrltktrunguong_min=MIN(mdrltktrunguong).

if mdrltktrunguong_min>0 rltktrunguong_sae_kp=rltktrunguongkp.

* roi loan tam than

if chanankp=1 or tamthankp=1 rltamthankp=1.

recode rltamthankp (missing=0).

if id=lag(id) and lag(rltamthankp)=1 rltamthankp=lag(rltamthankp).

Do if ((id=lag(id)) and (rltamthankp=0)).

compute tgrltamthan=tte.

else if (id=lag(id) and rltamthankp=1 and lag(rltamthankp)=0).

compute tgrltamthan=tte.

else if (id=lag(id) and rltamthankp=1 and lag(rltamthankp)=1).

compute tgrltamthan=lag(tgrltamthan).

ELSE.

compute tgrltamthan=tte.

end if.

if mdtamthan=5 or mdchanan=5 mdrltamthan=5.

if mdtamthan=4 or mdchanan=4 mdrltamthan=4.

if mdtamthan=3 or mdchanan=3 mdrltamthan=3.

if mdtamthan=2 or mdchanan=2 mdrltamthan=2.

if mdtamthan=1 or mdchanan=1 mdrltamthan=1.

Value labels mdrltamthan

1 'tu vong' 2 'tinh mang' 3 'tan tat' 4 'keo dai nam vien' 5 'chi dinh nhap vien'.

EXECUTE.

AGGREGATE

/OUTFILE=* MODE=ADDVARIABLES

/BREAK=Table1x.ID

/mdrltamthan_min=MIN(mdrltamthan).

if mdrltamthan_min>0 rltamthan_sae_kp=rltamthankp.

* Roi loan than kinh ngoai vi

if mdtkngoaivikp=1 or mdtkngoaivikp=2 or mdtkngoaivikp=3 or mdtkngoaivikp=4 or mdtkngoaivikp=5 tkngoaivi_sae_kp=mdtkngoaivikp.

* Roi loan thinh giac-tien dinh

if mdtiendinhkp=1 or mdtiendinhkp=2 or mdtiendinhkp=3 or mdtiendinhkp=4 or mdtiendinhkp=5 tiendinh_sae_kp=mdtiendinhkp.

* huyet hoc

if mdhuyethockp=1 or mdhuyethockp=2 or mdhuyethockp=3 or mdhuyethockp=4 or mdhuyethockp=5 huyethoc_sae_kp=mdhuyethockp.

* gan

if (mddocganxd=5 or mddocgannn=5) and kpdocgana=1 mdgocgan=5.

if (mddocganxd=4 or mddocgannn=4) and kpdocgana=1 mdgocgan=4.

if (mddocganxd=3 or mddocgannn=3) and kpdocgana=1 mdgocgan=3.

if (mddocganxd=2 or mddocgannn=2) and kpdocgana=1 mdgocgan=2.

if (mddocganxd=1 or mddocgannn=1) and kpdocgana=1 mdgocgan=1.

Value labels mdgocgan

1 'tu vong' 2 'tinh mang' 3 'tan tat' 4 'keo dai nam vien' 5 'chi dinh nhap vien'.

EXECUTE.

AGGREGATE

/OUTFILE=* MODE=ADDVARIABLES

/BREAK=Table1x.ID

/mdgocgan_min=MIN(mdgocgan).

if mdgocgan_min>0 docgan_sae_kp=kpdocgana.

*qua man

if mdquamankp=1 or mdquamankp=2 or mdquamankp=3 or mdquamankp=4 or mdquamankp=5 quanman_sae_kp=mdquamankp.

* doc than

RECODE bc1 ('14=doc than'=1) ('14=doc than nghi ngo'=1) ('142=doc than nghi ngo'=1) ('142=nghi ngo doc than'=1) into aethan1.

recode motabc1 ('159'=159) ('116'=116) ('149'=149) ('138'=138) ('125'=125) ('147'=147) ('203'=203) ('136'=136) ('134'=134) ('131'=131) ('196'=196) ('172'=172)

('124'=124) ('157'=157) ('139'=139) ('182'=182) ('140'=140) ('121'=121) ('126'=126) ('120'=120) ('383'=383) ('158'=158) ('152'=152) ('122'=122) ('145'=145) ('135'=135)

('143'=143) ('142'=142) ('117'=117) ('223'=223) ('166.2'=166.2) ('151'=151) ('155.2'=155.2) ('123.8'=123.8) ('175'=175) ('148'=148) ('132'=132) ('130.9'=130.9)

('180.4'=180.4) ('120.7'=120.7) ('119.1'=119.1) ('108.5'=108.5) ('248.5'=248.5) ('203.2'=203.2) ('185.8'=185.8) ('121.3'=121.3) ('109.7'=109.7) ('104.7'=104.7)

('129.5'=129.5) ('109.2'=109.2) ('216'=216) ('150.1'=150.1) ('170.5'=170.5) ('110.3'=110.3) ('152.6'=152.6) ('131.7'=131.7) ('141.5'=141.5) ('134.7'=134.7)

('190.8'=190.8) ('215.5'=215.5) ('105.7'=105.7) ('114.7'=114.7) ('115.8'=115.8) ('123.6'=123.6) ('188.4'=188.4) ('150.5'=150.5) ('112.8'=112.8) ('112.3'=112.3)

('111.5'=111.5) ('138.2'=138.2) ('139.5'=139.5) ('193.7'=193.7) ('192.3'=192.3) ('176'=176) ('231.1'=231.1) ('237.2'=237.2) ('112'=112) ('115.9'=115.9)

('149.4'=149.4) ('205.4'=205.4) ('142.1'=142.1) ('123.9'=123.9) ('169.2'=169.2) ('155.8'=155.8) ('193.9'=193.9) ('158.5'=158.5) ('153.2'=153.2) ('126.9'=126.9)

('263.1'=263.1) ('130.8'=130.8) ('216.4'=216.4) ('143'=143) ('145.2'=145.2) ('132.9'=132.9) ('185.4'=185.4) ('144'=144) ('172.2'=172.2) ('171.5'=171.5) ('125.3'=125.3)

('143.9'=143.9) ('204.8'=204.8) ('209.9'=209.9) ('114.8'=114.8) ('111.2'=111.2) ('105.4'=105.4) ('131.3'=131.3) ('120.1'=120.1) ('128'=128) ('119.2'=119.2)

('130.5'=130.5) ('132.5'=132.5) ('145'=145) ('150'=150) ('136.4'=136.4) ('149.8'=149.8) ('136.6'=136.6) ('143.0'=143) ('143.00'=143) ('148.50'=148.5) into creatinin1.

RECODE bc2 ('14=doc than'=1) ('14=doc than nghi ngo'=1) ('142=doc than nghi ngo'=1) ('142=nghi ngo doc than'=1) into aethan2.

recode motabc2 ('202'=202) ('200.4'=200.4) ('169'=169) ('144'=144) ('173.7'=173.7) ('164.8'=164.8) ('145'=145) ('129.5'=129.5) ('123.6'=123.6) ('134'=134) ('111.4'=111.4) ('119.2'=119.2)

('178.7'=178.7) ('148.50'=148.5) into creatinin2.

RECODE bc3 ('14=doc than'=1) ('14=doc than nghi ngo'=1) ('142=doc than nghi ngo'=1) ('142=nghi ngo doc than'=1) into aethan3.

recode motabc3 ('166'=166) ('170'=170) ('144'=144) ('129.5'=129.5) ('109.7'=109.7) ('123.7'=123.7) ('172.6'=172.6) ('216'=216) ('150.1'=150.1) ('120.8'=120.8) ('116'=116) ('190.2'=190.2)

('14.4'=14.4) ('144.3'=144.3) ('101.6'=101.6) ('114.7'=114.7) ('105'=105) ('120.2'=120.2) ('111.2'=111.2) ('104.7'=104.7) ('136'=136)

('143, 144'=144) ('126.4'=126.4) ('119.2'=119.2) into creatinin3.

RECODE bckhac ('14=doc than'=1) ('14=doc than nghi ngo'=1) ('142=doc than nghi ngo'=1) ('142=nghi ngo doc than'=1) into aethankhac.

recode motabckhac ('166.2'=166.2) ('129.5'=129.5) ('145'=145) ('143.9'=143.9) ('144.6'=144.6) ('150'=150) into creatininkhac.

compute aethan=max(aethan1,aethan2,aethan3,aethankhac).

compute creatinins=max(creatinin1,creatinin2,creatinin3,creatininkhac).

if gioia=0 crcrs=(140-tuoi)*nang/(72/88.4*creatinins)*0.85.

if gioia=1 crcrs=(140-tuoi)*nang/(72/88.4*creatinins).

recode nangbd (missing=47).

if gioia=0 crcr0=(140-tuoi)*nangbd/(72/88.4*cr0)*0.85.

if gioia=1 crcr0=(140-tuoi)*nangbd/(72/88.4*cr0).

compute giamgfr=(crcr0-crcrs)/crcr0*100.

compute tangcre=(creatinins-cr0)/cr0.

if tangcre>=1.5 or giamgfr>=25 dauhieudt=1.

if docthan=1 and dauhieudt=1 aki=1.

if creatinins=144 and Table1x.ID=116 aki=1.

if creatinins=144 and Table1x.ID=116 mdaki=1.

sort cases Table1x.ID(A) ngay(A) docthankp(D).

if (aki=1 and ((tangcre>=1.5 and tangcre<2) or (giamgfr>=25 and giamgfr<50))) mdaki=1.

if (aki=1 and ((tangcre>=2 and tangcre<3) or (giamgfr>=50 and giamgfr<75))) mdaki=2.

if (aki=1 and ((tangcre>=3) or (giamgfr>=75) or (creatinins>=353.6 and tangcre>44.2))) mdaki=3.

recode aki (missing=0).

recode mdaki (missing=0).

recode aethan (missing=0).

if docthan=1 and aethan=0 x=1.

compute akikp=aki.

if Table1x.ID=lag(Table1x.ID) and lag(akikp)=1 akikp=lag(akikp).

Do if ((akikp=0)).

compute taki=tte.

else if (Table1x.ID=lag(Table1x.ID) and akikp=1 and lag(akikp)=0).

compute taki=tte.

else if (Table1x.ID=lag(Table1x.ID) and akikp=1 and lag(akikp)=1).

compute taki=lag(taki).

ELSE.

compute taki=tte.

end if.

AGGREGATE

/OUTFILE=* MODE=ADDVARIABLES

/BREAK=Table1x.ID

/mdakikp=MAX(mdaki).

VALUE LABELS mdaki

1 'Nguy cơ' 2 'Tổn thương' 3 'Suy thận'.

VALUE LABELS mdakikp

1 'Nguy cơ' 2 'Tổn thương' 3 'Suy thận'.

if docthan=1 and aki=1 and (mddocthan=1 or mddocthan=2 or mddocthan=3 or mddocthan=4 or mddocthan=5) mdaki=mddocthan.

AGGREGATE

/OUTFILE=* MODE=ADDVARIABLES

/BREAK=Table1x.ID

/mdaki_min=MIN(mdaki).

if mdaki_min>0 aki_sae_kp=1.

*tang uric

if mdtangurickp=1 or mdtangurickp=2 or mdtangurickp=3 or mdtangurickp=4 or mdtangurickp=5 uric_sae_kp=mdtangurickp.

* roi loan tam nhin

if mdthigiackp=1 or mdthigiackp=2 or mdthigiackp=3 or mdthigiackp=4 or mdthigiackp=5 tamnhin_sae_kp=mdthigiackp.

*ha kali

if mdhakalikp=1 or mdhakalikp=2 or mdhakalikp=3 or mdhakalikp=4 or mdhakalikp=5 hakali_sae_kp=mdhakalikp.

*Suy giap

if mdsuygiapkp=1 or mdsuygiapkp=2 or mdsuygiapkp=3 or mdsuygiapkp=4 or mdsuygiapkp=5 suygiap_sae_kp=mdsuygiapkp.

*dau khop

if mddaukhopkp=1 or mddaukhopkp=2 or mddaukhopkp=3 or mddaukhopkp=4 or mddaukhopkp=5 daukhop_sae_kp=mddaukhopkp.

*roi loan glucose

if mdglucosekp=1 or mdglucosekp=2 or mdglucosekp=3 or mdglucosekp=4 or mdglucosekp=5 glucose_sae_kp=mdglucosekp.

* phan ve

if mdsockp=1 or mdsockp=2 or mdsockp=3 or mdsockp=4 or mdsockp=5 soc_sae_kp=mdsockp.

*AE chung

sort cases Table1x.ID(A) ngay(A).

if rloanthoakp=1 or rltktrunguongkp=1 or rltamthankp=1 or tkngoaivikp=1 or tiendinhkp=1 or huyethockp=1 or kpdocgana=1 or

quamankp=1 or akikp=1 or tangurickp=1 or thigiackp=1 or hakalikp=1 or suygiapkp=1 or daukhopkp=1 or glucosekp=1 or sockp aechungkp=1.

recode aechungkp (missing=0).

Do if ((id=lag(id)) and (aechungkp=0)).

compute tgaechung=tte.

else if (id=lag(id) and aechungkp=1 and lag(aechungkp)=0).

compute tgaechung=tte.

else if (id=lag(id) and aechungkp=1 and lag(aechungkp)=1).

compute tgaechung=lag(tgaechung).

ELSE.

compute tgaechung=tte.

end if.

*SAE chung

if rloanthoa_sae_kp>=1 or rltktrunguong_sae_kp>=1 or rltamthan_sae_kp>=1 or tkngoaivi_sae_kp>=1 or

tiendinh_sae_kp>=1 or huyethoc_sae_kp>=1 or docgan_sae_kp>=1 or quanman_sae_kp>=1 or aki_sae_kp>=1 or uric_sae_kp>=1 or tamnhin_sae_kp>=1 or

hakali_sae_kp>=1 or suygiap_sae_kp>=1 or daukhop_sae_kp>=1 or glucose_sae_kp>=1 or soc_sae_kp saekp=1.

recode saekp (missing=0).

Do if ((id=lag(id)) and (saekp=0)).

compute tgsaechung=tte.

else if (id=lag(id) and saekp=1 and lag(saekp)=0).

compute tgsaechung=tte.

else if (id=lag(id) and saekp=1 and lag(saekp)=1).

compute tgsaechung=lag(tgsaechung).

ELSE.

compute tgsaechung=tte.

end if.

**LIEU KM GAN NHAT TRUOC KHI XUAT HIEN AE, neu khong co AE lay lieu cao nhat**

sort cases Table1x.ID(A) ngay(A) docthankp(D).

if (Table1x.ID = LAG(Table1x.ID)) AND docthankp=0 and lag(docthankp)=1 docthankp=lag(docthankp).

compute lieukmw=lieukma/nanga.

compute lieukmw_docthan=lieukmw.

do if (Table1x.ID = LAG(Table1x.ID)) AND lieukmw_docthan>lag(lieukmw_docthan).

compute lieukmw_docthan=lieukmw_docthan.

ELSE IF (Table1x.ID=lag(Table1x.ID) AND lieukmw_docthan<=lag(lieukmw_docthan)).

COMPUTE lieukmw_docthan=lag(lieukmw_docthan).

end if.

IF (Table1x.ID = LAG(Table1x.ID)) AND docthankp=1 and lag(docthankp)=0 lieukmw_docthan=lag(lieukmw_docthan).

if (Table1x.ID = LAG(Table1x.ID)) AND docthankp=1 and lag(docthankp)=1 lieukmw_docthan=lag(lieukmw_docthan).

do if lieukmw_docthan>0.

compute km_docthan=1.

else if lieukmw_docthan=0.

compute km_docthan=0.

end if.

EXECUTE.

RECODE lieukmw_docthan (0=SYSMIS).

EXECUTE.

* tong lieu tich luy km chia thoi gian va can nang

if $CASENUM=1 lieutichluy_km=lieukma*tte.

if (Table1x.ID <> LAG(Table1x.ID)) lieutichluy_km=lieukma*tte.

if (Table1x.ID = LAG(Table1x.ID)) lieutichluy_km=lag(lieukma)*interval+lag(lieutichluy_km).

compute lieukmtbw=lieutichluy_km/tte/nanga.

compute lieukmtbw_docthan=lieukmtbw.

if (Table1x.ID = LAG(Table1x.ID)) AND docthankp=1 and lag(docthankp)=1 lieukmtbw_docthan=lag(lieukmtbw_docthan).

EXECUTE.

recode lieukmtbw_docthan (0=SYSMIS).

EXECUTE.

**LIEU cm GAN NHAT TRUOC KHI XUAT HIEN AE, neu khong co AE lay lieu cao nhat**

compute lieucmw=lieucma/nanga.

compute lieucmw_docthan=lieucmw.

do if (Table1x.ID = LAG(Table1x.ID)) AND lieucmw_docthan>lag(lieucmw_docthan).

compute lieucmw_docthan=lieucmw_docthan.

ELSE IF (Table1x.ID=lag(Table1x.ID) AND lieucmw_docthan<=lag(lieucmw_docthan)).

COMPUTE lieucmw_docthan=lag(lieucmw_docthan).

end if.

IF (Table1x.ID = LAG(Table1x.ID)) AND docthankp=1 and lag(docthankp)=0 lieucmw_docthan=lag(lieucmw_docthan).

if (Table1x.ID = LAG(Table1x.ID)) AND docthankp=1 and lag(docthankp)=1 lieucmw_docthan=lag(lieucmw_docthan).

do if lieucmw_docthan>0.

compute cm_docthan=1.

else if lieucmw_docthan=0.

compute cm_docthan=0.

end if.

EXECUTE.

RECODE lieucmw_docthan (0=SYSMIS).

EXECUTE.

* tong lieu tich luy cm chia thoi gian va can nang

if $CASENUM=1 lieutichluy_cm=lieucma*tte.

if (Table1x.ID <> LAG(Table1x.ID)) lieutichluy_cm=lieucma*tte.

if (Table1x.ID = LAG(Table1x.ID)) lieutichluy_cm=lag(lieucma)*interval+lag(lieutichluy_cm).

compute lieucmtbw=lieutichluy_cm/tte/nanga.

compute lieucmtbw_docthan=lieucmtbw.

if (Table1x.ID = LAG(Table1x.ID)) AND docthankp=1 and lag(docthankp)=1 lieucmtbw_docthan=lag(lieucmtbw_docthan).

EXECUTE.

recode lieucmtbw_docthan (0=SYSMIS).

EXECUTE.

**LIEU am GAN NHAT TRUOC KHI XUAT HIEN AE, neu khong co AE lay lieu cao nhat**

compute lieuamw=lieuama/nanga.

compute lieuamw_docthan=lieuamw.

do if (Table1x.ID = LAG(Table1x.ID)) AND lieuamw_docthan>lag(lieuamw_docthan).

compute lieuamw_docthan=lieuamw_docthan.

ELSE IF (Table1x.ID=lag(Table1x.ID) AND lieuamw_docthan<=lag(lieuamw_docthan)).

COMPUTE lieuamw_docthan=lag(lieuamw_docthan).

end if.

IF (Table1x.ID = LAG(Table1x.ID)) AND docthankp=1 and lag(docthankp)=0 lieuamw_docthan=lag(lieuamw_docthan).

if (Table1x.ID = LAG(Table1x.ID)) AND docthankp=1 and lag(docthankp)=1 lieuamw_docthan=lag(lieuamw_docthan).

do if lieuamw_docthan>0.

compute am_docthan=1.

else if lieuamw_docthan=0.

compute am_docthan=0.

end if.

EXECUTE.

RECODE lieuamw_docthan (0=SYSMIS).

EXECUTE.

* tong lieu tich luy am chia thoi gian va can nang

if $CASENUM=1 lieutichluy_am=lieuama*tte.

if (Table1x.ID <> LAG(Table1x.ID)) lieutichluy_am=lieuama*tte.

if (Table1x.ID = LAG(Table1x.ID)) lieutichluy_am=lag(lieuama)*interval+lag(lieutichluy_am).

compute lieuamtbw=lieutichluy_am/tte/nanga.

compute lieuamtbw_docthan=lieuamtbw.

if (Table1x.ID = LAG(Table1x.ID)) AND docthankp=1 and lag(docthankp)=1 lieuamtbw_docthan=lag(lieuamtbw_docthan).

EXECUTE.

recode lieuamtbw_docthan (0=SYSMIS).

EXECUTE.

**LIEU thuoctiem GAN NHAT TRUOC KHI XUAT HIEN AE, neu khong co AE lay lieu cao nhat**

compute lieuthuoctiema=lieukma+lieucma+lieuama.

compute lieuthuoctiemw=lieuthuoctiema/nanga.

compute lieuthuoctiemw_docthan=lieuthuoctiemw.

do if (Table1x.ID = LAG(Table1x.ID)) AND lieuthuoctiemw_docthan>lag(lieuthuoctiemw_docthan).

compute lieuthuoctiemw_docthan=lieuthuoctiemw_docthan.

ELSE IF (Table1x.ID=lag(Table1x.ID) AND lieuthuoctiemw_docthan<=lag(lieuthuoctiemw_docthan)).

COMPUTE lieuthuoctiemw_docthan=lag(lieuthuoctiemw_docthan).

end if.

IF (Table1x.ID = LAG(Table1x.ID)) AND docthankp=1 and lag(docthankp)=0 lieuthuoctiemw_docthan=lag(lieuthuoctiemw_docthan).

if (Table1x.ID = LAG(Table1x.ID)) AND docthankp=1 and lag(docthankp)=1 lieuthuoctiemw_docthan=lag(lieuthuoctiemw_docthan).

do if lieuthuoctiemw_docthan>0.

compute thuoctiem_docthan=1.

else if lieuthuoctiemw_docthan=0.

compute thuoctiem_docthan=0.

end if.

EXECUTE.

RECODE lieuthuoctiemw_docthan (0=SYSMIS).

EXECUTE.

* tong lieu tich luy thuoctiem chia thoi gian va can nang

if $CASENUM=1 lieutichluy_thuoctiem=lieuthuoctiema*tte.

if (Table1x.ID <> LAG(Table1x.ID)) lieutichluy_thuoctiem=lieuthuoctiema*tte.

if (Table1x.ID = LAG(Table1x.ID)) lieutichluy_thuoctiem=lag(lieuthuoctiema)*interval+lag(lieutichluy_thuoctiem).

compute lieuthuoctiemtbw=lieutichluy_thuoctiem/tte/nanga.

compute lieuthuoctiemtbw_docthan=lieuthuoctiemtbw.

if (Table1x.ID = LAG(Table1x.ID)) AND docthankp=1 and lag(docthankp)=1 lieuthuoctiemtbw_docthan=lag(lieuthuoctiemtbw_docthan).

EXECUTE.

recode lieuthuoctiemtbw_docthan (0=SYSMIS).

EXECUTE.

*xu tri

if (xutribc1a=1 or xutribc2a=1 or xutribc3a=1 or xutribckhaca=1) giamlieu=1.

if (xutribc1a=2 or xutribc2a=2 or xutribc3a=2 or xutribckhaca=2) ngunggiam=1.

if (xutribc1a=3 or xutribc2a=3 or xutribc3a=3 or xutribckhaca=3) doithuoc=1.

if giamlieu=1 or ngunggiam=1 or doithuoc=1 tdphacdo=1.

AGGREGATE

/OUTFILE=* MODE=ADDVARIABLES

/BREAK=Table1x.ID

/tdphacdo_max=MAX(tdphacdo).

AGGREGATE

/OUTFILE=* MODE=ADDVARIABLES

/BREAK=Table1x.ID

/doithuoc_max=MAX(doithuoc).

AGGREGATE

/OUTFILE=* MODE=ADDVARIABLES

/BREAK=Table1x.ID

/ngunggiam_max=MAX(ngunggiam).

AGGREGATE

/OUTFILE=* MODE=ADDVARIABLES

/BREAK=Table1x.ID

/giamlieu_max=MAX(giamlieu).

*tach file kaplan

sort cases Table1x.ID(A) ngay(A).

CREATE Table1x.IDlead=LEAD(Table1x.ID,1).

recode Table1x.IDlead (misssing=0).

DATASET COPY km12.

DATASET ACTIVATE km12.

FILTER OFF.

USE ALL.

SELECT IF (Table1x.ID<>Table1x.IDlead).

EXECUTE.

DATASET ACTIVATE DataSet1.
