# Supplementary material for: Active surveillance for adverse events in patients on longer treatment regimens for multidrug-resistant tuberculosis in Viet Nam
Source: PLoS One. 2021 Sep 7;16(9):e0255357. doi: 10.1371/journal.pone.0255357 (PMC8423256; doi:10.1371/journal.pone.0255357)
Supplement: S4 File — (DOCX) [file pone.0255357.s007.docx]

setwd("E:/Dropbox/b6.3/Xuli lai dang bao/Manuscript/Plos one")

library(dplyr)

library(readr)

data <- read_csv("S2 File.csv")

View(data)

#Lable varibales

library(expss)

data = apply_labels(data,

                    id = "ID",

                    ngaydau = "Start date of treatment",

                    tuoi = "Age",cao = "Height",bmi = "BMI",nangbd = "Weight Baseline", ngay = "Date of visit",

                    gioia = "Gender", donvia = "Province",

                    mackemtha = "Hypertension",mackemdaithaoduong = "Diabetes mellitus",

                    mackembenhsuythan = "Renal dysfunction",mackembenhgan = "Hepatic disorders",

                    mackembenhduongtieuhoa = "Gastrointestinal disorders",mackembenhthinhgiac = "Hearing loss",

                    mackembenhcoxuongkhop = "Arthralgia",tinhtrangsuykiet = "Fatigue",tinhtrangnghienruou = "Alcohol dependence",

                    tinhtrangnhiemhiv = "HIV infection",loaibn = "Outcome of previous treatment",

                    phacdoa = "Initial MDR-TB Treatment Regimens",tg = "Duration of treatment in months",

                    kqua = "Follow-up outcomes",tkngoaivikp = "AE Peripheral neuropathy",daukhopkp = "AE Arthralgia",

                    quamankp = "AE Dermatologic reactions",tiendinhkp = "AE Hearing loss or vestibular disorders",

                    thigiackp = "AE Visual impairment",hakalikp = "AE Hypokalaemia",tangurickp = "AE Hyperuricemia",

                    huyethockp = "AE Hematologic disorders",kpdocgana = "AE Nephrotoxicity",sockp = "AE Anaphylactic reactions",

                    glucosekp = "AE Glucose metabolism disorders",suygiapkp = "AE Hypothyroidism",

                    rltamthankp = "AE Psychiatric disorders",rltktrunguongkp = "AE Central nervous system disorders",

                    rloanthoakp = "AE Gastrointestinal disorders",aechungkp = "AE",akikp = "AE Nephrotoxicity",

                    taki = "Time to Nephrotoxicity",rloanthoa_sae_kp = "SAE Gastrointestinal disorders",

                    rltktrunguong_sae_kp = "SAE Central nervous system disorders",

                    rltamthan_sae_kp = "SAE Psychiatric disorders",tkngoaivi_sae_kp = "SAE Peripheral neuropathy",

                    tiendinh_sae_kp = "SAE Hearing loss or vestibular disorders",huyethoc_sae_kp = "SAE Hematologic disorders",

                    docgan_sae_kp = "SAE Hepatoxicity",quanman_sae_kp = "SAE Dermatologic reactions",

                    uric_sae_kp = "SAE Hyperuricemia",tamnhin_sae_kp = "SAE Visual impairment",

                    hakali_sae_kp = "SAE Hypokalaemia",suygiap_sae_kp = "SAE Hypothyroidism",

                    daukhop_sae_kp = "SAE Arthralgia",glucose_sae_kp = "SAE Glucose metabolism disorders",

                    aki_sae_kp = "SAE Nephrotoxicity",soc_sae_kp = "SAE Anaphylactic reactions",saekp = "SAE",

                    tichluythuoctiema_aki = "Dose of injectable drugs",giamlieu_max = "Dose reduction",

                    ngunggiam_max = "Permanent discontinuation",doithuoc_max = "Withdrawal and replacement",

                    tdphacdo_max = "TB therapeutic intervention",thuoctc_max = "Other medical intervention",

                    ddtm_max = "Life-threatening",ttvv_max = "Permanent disability",

                    nvnv_max = "Hospitalization or prolongation of hospitalization")

val_lab(data$gioia) = num_lab("

            1 Male

            0 Female")

data$gioia <- as.character(data$gioia)

val_lab(data$donvia) = num_lab("

            1 Binh Dinh

            2 Binh Thuan

            3 74 Trung Uong

            4 Quang Nam

            5 Thanh Hoa

            6 Can Tho

            7 Ha Noi

            8 TPHCM

            9 Nam Dinh

")

data$donvia <- as.factor(data$donvia)

val_lab(data$loaibn) = num_lab("

            1 No history of TB treatment

            2 Completed

            3 Treatment failure

            4 Lost to follow-up

            99 No information

")

data$loaibn <- as.character(data$loaibn)

val_lab(data$phacdoa) = num_lab("

            1 IVa

            2 IVb

            3 Individual")

data$phacdoa <- as.character(data$phacdoa)

val_lab(data$kqua) = num_lab("

            1 Cure/completion

            2 All-cause death

            3 Not evaluated

            4 Lost to follow-up

            5 Failure

")

data$kqua <- as.character(data$kqua)

#Description

library(summarytools)

view(dfSummary(data))

library(DescTools)

Desc(data$tuoi)

Desc(data$nangbd)

Desc(data$tg)

#Cox regression

library(survival)

library(survminer)

surv.t=data$taki

cens=data$akikp

cox=coxph(Surv(surv.t, cens)~ tuoi+gioia+bmi+mackemdaithaoduong+mackembenhsuythan+

            tinhtrangnghienruou+tichluythuoctiema_aki, data=data)

summary(cox)
